# Simple Bagged Movement Models for Telemetry Data

**DOI:** 10.1002/ece3.72060

**Published:** 2025-09-07

**Authors:** Andrew B. Whetten, Trevor J. Hefley, David A. Haukos, Dustin E. Brewer

**Affiliations:** ^1^ Department of Statistics Kansas State University Manhattan Kansas USA; ^2^ Division of Biology Kansas State University Manhattan Kansas USA; ^3^ Avian Population Studies, Cornell Lab of Ornithology Cornell University Ithaca New York USA; ^4^ U.S. Geological Survey, Kansas Cooperative Fish and Wildlife Research Unit Kansas State University Manhattan Kansas USA; ^5^ School of Natural Resources Lake Superior State University Sault Sainte Marie Michigan USA

**Keywords:** animal movement, animal movement models, bagging, bootstrap, ensemble, king rail, machine learning, movement ecology, mule deer, telemetry data

## Abstract

Determining which statistical methods are appropriate for data is both user and data dependent and prone to change as new methodology becomes available. This process encompasses model ideation, model selection, and determining appropriate use of statistical methods. Literature on models for animal movement emerging in the past two decades has yielded a rich collection of statistical methods garnering much deserved positive attention. Among such efforts, there is limited investigation of the broader place for simple machine learning methodology in animal movement modeling. We propose a bagged (i.e., bootstrap aggregated) animal movement model using simple, off‐the‐shelf machine learning algorithms. The model is intuitive, retains statistical inference about characteristics of animal movement (i.e., estimated from model‐based summary statistics), and only requires knowledge of elementary statistical and machine learning analysis to understand. We show by simulation that our model can provide unbiased estimates of pertinent characteristics of animal movement (e.g., daily displacement) in the presence of large and realistic location error. We believe that increasing accessible literature on simple machine learning animal movement models provides valuable pedagogical and practical support for researchers using statistical models to study animal movement.

## Graphical abstract

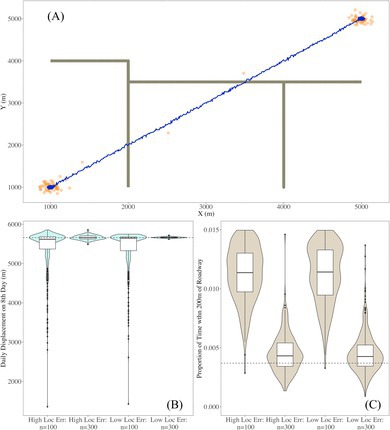

## 1 Introduction

Movement ecology in the past two decades has experienced revolutionary change. Efforts include the unification of organismal movement research and preparation for new research frontiers as movement ecology emerges as a big data discipline (Nathan et al. 2008, 2022; Kays et al. 2015; Hooten et al. 2017; Farley et al. 2018; Thums et al. 2018). However, there is a continued need to overcome challenges that limit the applied relevance of research in movement ecology across disciplines (Katzner and Arlettaz 2020). One challenge is the democratization of model‐based analysis of animal movement data to support researchers in disciplines beyond ecology, conservation, and wildlife management, such as the environmental sciences, engineering, and agriculture. By democratization, we refer to efforts to match potentially diverse user experience and background with available statistical methodology for animal movement modeling.

The last two decades of research have yielded a rich collection of statistical methods to analyze animal telemetry data (i.e., recorded locations of an animal over time), a common type of data used to study the movements of animals (Hooten et al. 2017). Within this collection, mechanistic movement models (e.g., state‐space and Bayesian hierarchical movement models) have provided a statistically principled approach to model animal movement with bespoke structure that characterizes location error and underlying stochastic processes and behavioral states governing movement. However, these models generally require a handful of traditional model‐based and computational assumptions. There may be many instances where the needs of researchers may be better suited to more flexible models with fewer traditional and computational assumptions. Initially, a primary need of research involving telemetry data is knowledge discovery, and the gaining of such knowledge from data can support research spanning basic animal ecology and conservation science (Frawley et al. 1992; Domingos 1999). However, beyond knowledge discovery, telemetry data have the potential to support pressing practical needs faced by scientists and engineers such as infrastructure design and agricultural practices (Marques et al. 2014; Van Doren et al. 2021; Swinton et al. 2007; DeClerck et al. 2016; Decker and Chase 1997; Dressel et al. 2018).

As we consider the broad objectives of researchers spanning knowledge discovery and practical application, we believe that animal movement modeling could be more accessible to those with more general quantitative training but lack specialized training in movement ecology. This may require expositions on more utilitarian statistical models that can meet fundamental needs of a wide audience of researchers. A familiar and fundamental tenet of modern science is to appropriately consider model choice and prioritize simpler models when appropriate (Domingos 1999). Some of the simplest machine learning algorithms, such as nearest neighbor regression and regression trees, have the potential flexibility to model complex animal movement. These models are free of traditional model‐based assumptions (e.g., distribution of residuals, specification of sub‐models of a Bayesian hierarchical model) and computational assumptions (e.g., Markov chain Monte Carlo convergence, mixing rate, ergodicity). Algorithms such as nearest neighbor regression and regression trees use local information to make predictions, which allows for abrupt or complex changes in the process to be modeled with relatively strong computational stability. However, until recently, investigation of the value of machine learning in animal movement modeling literature is limited (Wijeyakulasuriya et al. 2020; Rieber et al. 2024).

Valid statistical inference about animal location at any time from telemetry data is foundational to describing with evidence how an animal interacts with its environment and other animals (Whetten et al. 2024). However, perhaps more important, an animal movement model should have desirable frequentist properties such as minimal bias and nominal (actual) coverage probabilities for estimates of derived quantities (i.e., model‐based summary statistics describing a movement path). Obtaining estimates of common derived quantities (e.g., daily displacement, time spent near infrastructure) is a primary objective because such descriptors can support knowledge discovery and practical decision‐making pertaining to a species. If simple machine learning algorithms can provide ease of use and valid statistical inference about animal movement with desirable frequentist properties, then such methods should be considered as a possible alternative to specialized approaches that require unique user training (Wijeyakulasuriya et al. 2020; Rieber et al. 2024).

The specialized language and training required to implement and translate statistical animal movement models across applications may hinder more widespread use of animal telemetry data by disciplines such as environmental sciences, engineering, and agriculture (Hooten et al. 2017). As examples, there are continuing needs to connect analysis of animal movement to research in infrastructure design (Beben 2016), environmental sustainability (Cozzi et al. 2015), and agricultural production (Mathews 2010). Statistical literature on animal movement modeling has supported movement ecologist education and application of statistical models for telemetry data (Hooten et al. 2017). As needs to analyze telemetry data increase across several disciplines, we believe that providing statistical learning methodology for animal movement modeling will increase the accessibility of the field for a broader audience (Popovic et al. 2024).

How can researchers obtain valid statistical inference about animal movement from telemetry data using simple machine learning algorithms? An exceptional place to begin such a discussion is bagging (i.e., bootstrap aggregation) models of simple machine learning algorithms (Breiman 1996; James et al. 2013). Bagging is a versatile modeling technique that is relatively easy to translate across applications, which makes it an appealing option for those engaging in interdisciplinary research. There has been an exponential increase in the use of standard machine learning algorithms over the past 30 years, which is indicative of their wide utility and accessibility and global efforts to train upcoming researchers in machine learning (Breiman et al. 1984; Breiman 1996; Breiman 2001; Hastie et al. 2009; James et al. 2013). Besides bagging, other potential starting places can include constructing animal movement models using generalized additive models and smoothing splines (Wood 2017; Ramsay and Silverman 2005; Whetten 2021). We have chosen to focus on bagged machine learning algorithms due to their popularity, flexibility, and potential reduction of traditional assumptions placed on the model.

We propose a bagged machine learning animal movement model that uses simple, off‐the‐shelf machine learning algorithms (e.g., nearest neighbor regression or regression trees) to learn the relationship between time and animal location from telemetry data (e.g., from GPS) to estimate derived quantities of interest such as daily displacement or time spent near infrastructure (Hastie et al. 2009). We illustrate how bagging supports valid statistical inference about the location of an animal at any time if telemetry data have negligible location error (Fleming et al. 2020). We assessed bias and coverage probabilities in the estimation of some common derived quantities from animal movement models when location error increased substantially.

From a pedagogical perspective, our proposed animal movement model is valuable in classroom settings for scientists and engineers who want to learn how to fit and utilize animal movement models. Our proposed animal movement model was efficient to code using standard statistical computing software, such as R or Python, under the assumption that the user has an introductory familiarity with regression analysis, bootstrap sampling (i.e., sampling observations with replacement), and machine learning. From a research perspective, our model illustrates deliberate democratization of animal movement modeling through bagged simple machine learning algorithms.

## 2 Materials and Methods

Our framework for bagged machine learning animal movement models requires an introductory knowledge of linear regression (chapter 3 in James et al. 2013), bootstrap sampling (chapter 5 in James et al. 2013), and ensemble machine learning (chapter 8.2 in James et al. 2013). As our framework exemplifies the use of simple machine learning algorithms, we mention upfront that readers should not expect our framework to be appropriate for all telemetry data (Wang 2019; Wijeyakulasuriya et al. 2020). This framework is appropriate for a number of applications relevant to scientists and engineers, requires minimal adjustments by the user, and provides a more accessible entry into animal movement modeling for a broader audience. We provide a brief tutorial in Appendix S1 that supports readers through some fundamentals of bagging machine learning algorithms and tractably builds from simple one‐dimensional telemetry data to real animal telemetry data with thousands of recorded locations.

### 2.1 Bootstrapping Animal Telemetry Data

Bagging is a widely used ensemble machine learning algorithm known to improve stability and predictive performance for statistical classification and regression (Breiman 1996; Zhang and Ma 2012). Bagging is referred to as an ensemble because the algorithm collectively uses one or many machine learning algorithms (e.g., linear regression, *k*‐nearest neighbor regression, regression trees) to learn relationships and make predictions using bootstrap‐sampled realizations of a dataset (chapter 8.2.1 in James et al. 2013). Bagging is the foundation for some of the most widely cited machine learning algorithms in ecology, such as random forests (Breiman 2001; Cutler et al. 2007; Elith et al. 2008).

Bootstrap sampling of data is a simple procedure. For animal telemetry data, a bootstrap sample of data might only refer to a random sample with replacement of recorded locations for a monitored animal. Some recorded locations are repeatedly sampled and others are left unsampled. Any recorded locations that are present within a bootstrap sample are referred to as “in‐bag” recorded locations. Any recorded locations that are not present within a simple bootstrap sample are referred to as “out‐of‐bag” (OOB) recorded locations.

Consider a collection of recorded locations of an animal stored in a matrix, $S=\left[t_{i},s\left(t_{i}\right)\right]$, where i=1,…,n and $s\left(t_{i}\right)={\left(s_{1}\left(t_{i}\right)\;,\;s_{2}\left(t_{i}\right)\right)}^{\prime }$. Generally, S is an n×3 matrix where the scalars $s_{1}\left(t_{i}\right)$ and $s_{2}\left(t_{i}\right)$ are recorded coordinates (e.g., latitude and longitude) that identify an animal's location at time $t_{i}$. Time is treated as a continuous variable over which locations may be recorded irregularly. The matrix of recorded locations S can be thought of as training data. From training data S, we seek to learn a relationship between time and animal location.

We will draw many bootstrap samples from S, and refer to *b*th bootstrap sample from S as $S^{\left(b\right)}$, where b=1,…,B. A simple bootstrap sample $S^{\left(b\right)}$ will have the exact same structure and dimensions as S. However, as mentioned before, some recorded locations will be present one or more times while others are OOB. The conventional bootstrap procedures randomly samples recorded locations with replacement. Given there is an expected dependence between recorded locations, it may be advisable to consider one of many available bootstrap sampling procedures for dependent data (Carlstein 1986; Hall and Jing 1996; Politis et al. 1999). In brief, these procedures utilize various partitioning strategies of the feature space (i.e., times of recorded location in the data) prior to randomly sampling locations with replacement. We have used a subsampling procedure from Politis et al. (1999) in this work, but we leave investigation of bootstrap sampling procedure selection to future work.

### 2.2 A Bagged Machine Learning Animal Movement Model

**FIGURE 1:**
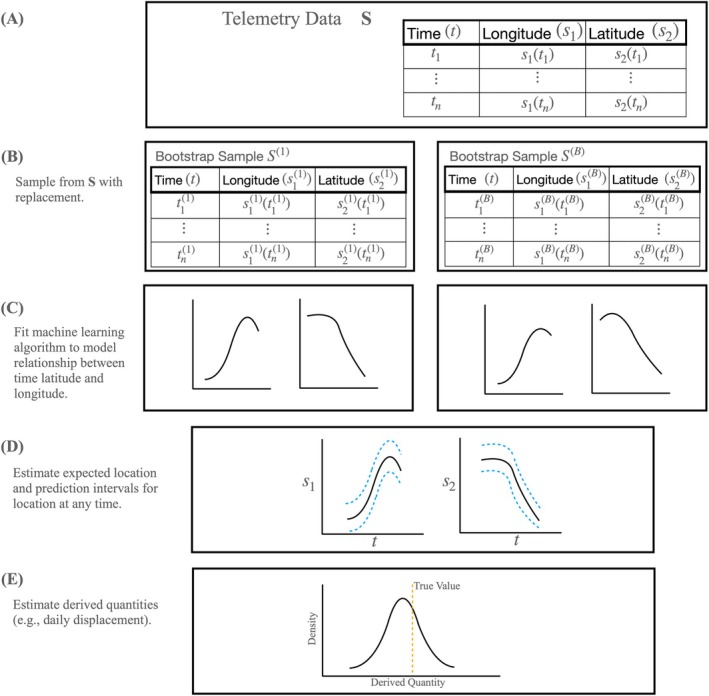
A framework for simple bagged machine learning animal movement models. Panel (A) provides a common representation of animal telemetry data, S. We will draw many bootstrap samples from S. Panel (B) illustrates the process of repeatedly bootstrap sampling telemetry data, where $S^{\left(b\right)}$ represents one of the many generated bootstrap‐sampled realizations of the telemetry data with b=1,…,B reordered with respect to time. Some recorded locations will be present one or more times in $S^{\left(b\right)}$ while others are left unsampled. Panel (C) illustrates using a machine learning algorithm to learn a relationship among time, latitude, and longitude for the *b*th bootstrap sample. Panel (D) represents the estimation of the expected location (black line) and predictive distribution (orange lines) for the location of an animal at any time. Panel (E) illustrates estimation of summary statistics (i.e., derived quantities) from the continuous movement path, which generally provide the greatest insights about animal movement. Examples of derived quantities include daily displacement and amount of time spent near infrastructure.

For each bootstrap sample $S^{\left(b\right)}$, we use a simple machine learning algorithm to learn the relationship between time and location (Figure 1A–C). The animal movement model aggregates information about this time–location relationship across all bootstrap samples. With this animal movement model, we can accomplish the following tasks:

1. Obtain a distribution for the expected location of an animal, $E\left(s\left(t\right)\right)$, at any time. We can obtain this distribution empirically using estimates of animal location from each $S^{\left(b\right)}$.
2. Estimate the expected value of animal location, $E\left(s\left(t\right)\right)=\hat{s}\left(t\right)=\left({\hat{s}}_{1}\left(t\right),{\hat{s}}_{2}\left(t\right)\right)$, where the index notation (*t*) denotes the location at time *t* (Figure 1C).
3. Obtain a predictive distribution for location $s\left(t\right)$ at any time $t\in \left(t_{1},t_{n}\right)$, and construct valid prediction intervals (Figure 1D).
4. Generate realizations of potential paths using the predictive distribution.
5. Summarize realizations of potential paths from the predictive distribution using derived quantities (Figure 1E).

For task (2), we use a machine learning algorithm to estimate a continuous movement path for each $S^{\left(b\right)}$, denoted by $\left({\hat{s}}_{1}^{\left(b\right)}\left(t\right),{\hat{s}}_{2}^{\left(b\right)}\left(t\right)\right)$. The expected value of animal location, $\left({\hat{s}}_{\mathrm{bag},1}^{\left(b\right)}\left(t\right),{\hat{s}}_{\mathrm{bag},2}^{\left(b\right)}\left(t\right)\right)$, is the average of B estimated movement paths. To obtain a bagged estimate of the expected value of animal location at any time, we generate predicted locations from our model using a fine time grid $T=\left\{t_{j}|j=1,\ldots ,m;t_{j}-t_{j-1}<\epsilon |t_{j}\in \left[t_{1},t_{m}\right]\right\}$. With a sufficiently dense time grid T, predicted locations can be used to represent animal movement at a finer resolution than the times of recorded locations in S.

For tasks (3) and (4), we can obtain a predictive distribution and estimate prediction regions for the location of an animal at any time using the distribution of prediction error from OOB data (Zhang et al. 2019). Briefly, for a bootstrap sample of the data, the machine learning algorithm used to learn the relationship between time and location does not use OOB recorded locations. Collectively, the bagged machine learning animal movement model will have many OOB predictions for each recorded location. Because of this, we can construct a prediction distribution for animal location at any time using the distribution of the residuals for the OOB data.

In a bagged machine learning animal movement model, we specify the machine learning algorithm used to learn the relationship between time and location. We can use a single machine learning algorithm such as *k*‐nearest neighbor regression (KNNR). Using KNNR requires the user to only specify one hyper‐parameter k (i.e., the number of nearest neighbors) to use for local predictions of location from time. With minimal adjustments to code, the user could allow the choice of machine learning algorithm to vary randomly (e.g., KNNR, regression trees, support vector regression). Such models emerged in early meta‐learning research and have gained popularity in recent decades (Wolpert 1992; Vilalta and Drissi 2002).

In our work, we focus on KNNR, which is a machine learning algorithm that uses only local information available from a feature space (e.g., time) to make predictions of some response (e.g., location). Predictions in each region are made using simple averages of nearest recorded locations with respect to time (Hastie et al. 2009). The KNNR algorithm is a fast, flexible algorithm that allows for abrupt (e.g., nearly discontinuous) transitions in a modeled continuous process.

### 2.3 A Focus on Derived Quantities

As shown in Figure 1D, estimation of a continuous animal path does not explicitly facilitate knowledge discovery because we are interested in characteristics of movement other than position. A continuous animal movement path needs summary statistics describing important attributes of animal movement (Johnson et al. 2011; Hobbs and Hooten 2015). We refer to these summary statistics as derived quantities. Derived quantities provide information that can expand our understanding of animal movement and have potential to address an array of research questions (Rieber et al. 2024). Simple examples of derived quantities could include daily displacement of an animal or relative frequency of road crossings during the monitoring period. These derived quantities represent attributes of a movement path that have an unknown, true value. It is important to assess frequentist properties of any estimates of derived quantities from our model. For example, we can estimate daily displacement from an animal movement model. Through simulation, we would like to understand the potential magnitude of bias present in our estimates of daily displacement. Further, we would like to know if coverage of a confidence interval for daily displacement can be expected to cover the true daily displacement with close to the correct confidence level, α.

### 2.4 Determining Appropriate Use of the Model

A bagged machine learning animal movement model provides an accessible option for researchers seeking to analyze telemetry data without specialized training in existing statistical models for animal movement. Further, our model requires less experience in model specification, adjusting code to re‐specify the model using a different machine learning algorithm, and model implementation. However, switching from KNNR to other machine learning algorithm does require that users have a working knowledge of hyper‐parameters used in these models and potential values that they take. KNNR is one of the simplest machine learning algorithms for regression, only requiring one hyper‐parameter. For a user to switch from KNNR to regression trees, it would be beneficial for the user to have familiarity with regression tree hyper‐parameters such as maximum depth (i.e., the maximum number of levels in the tree), minimum split criterion (i.e., the minimum number of observational units required to split a node within the tree), minimum terminal node size (i.e., the minimum number of observations allowed in a terminal node), and the complexity parameter (i.e., a parameter that controls the trade‐off between tree complexity and predictive accuracy).

Our major concern in the use of this model pertains to the magnitude of tracking device location error and its relation to research questions of interest. Ignoring device location error in many instances is too strong an assumption, as this has been shown to yield results that are inappropriate or misguided (Brost et al. 2015; McClintock et al. 2014; Gerber et al. 2018). However, there are scenarios where assuming location error is negligible suffices (Fleming et al. 2020). Within our model, the machine learning algorithm estimates a function characterizing animal movement. Deviations from this function are stochastic and a mixture of location error and actual movement resulting from underfitting of the machine learning algorithm. It is not always evident that accounting for location error in a more complicated model will render improvements to estimates of any quantities pertaining to stated research objectives (Fleming et al. 2020). We expand upon this discussion through our simulation study, where we assess frequentist properties of estimates of derived quantities from our bagged machine learning movement model in the presence of substantial location error. As currently presented, our approach does not illustrate how inference can be obtained about resource selection (Brost et al. 2015). Modeling the relationship between spatially referenced predictor variables and animal location may be practically obtained with this approach, but this is left to future work.

### 2.5 Simulation Study

We conducted a simulation study to assess frequentist properties (i.e., bias and coverage probability [CP]) for estimates of common derived quantities from our bagged machine learning animal movement model. We generated data for our study from a true movement path of a hypothetical animal, $z\left(t\right)=\left(z_{1}\left(t\right),z_{2}\left(t\right)\right)$. We consider two functions each representing distinct animal movement patterns:

1. Abrupt stepwise movement: This movement could represent an animal that abruptly switches from a stationary state to an extended state of directed higher velocity movement. Examples of this behavior can be exhibited by prairie grouse (e.g., *Tympanuchus* sp.) or rail (*Rallidae*) species as they move among fragmented regions of habitat. This is sometimes referred to as dispersal behavior (Clobert et al. 2001; Zollner and Lima 2005). Abrupt movement can sometimes occur in short bursts, and such movement events are critical to characterizing movements of some species. However, telemetry data may be deficient during these events. We use the following equations to represent abrupt stepwise movement in a two‐dimensional space for 15 days, with time measured in minutes:
$$z_{1}\left(t\right)=\left\{\begin{array}{cc} a+\epsilon_{z1}\left(t\right) & t\leq 10,600\\ \mathrm{bt}-c+\epsilon_{z1}\left(t\right) & 10,600\leq t\leq 11,000\\ a+\epsilon_{z1}\left(t\right) & 11,000\leq t\leq 21,600 \end{array}\right.$$
$$z_{2}\left(t\right)=\left\{\begin{array}{cc} a+\epsilon_{z2}\left(t\right) & t\leq 10,600\\ \mathrm{bt}-c+\epsilon_{z2}\left(t\right) & 10,600\leq t\leq 11,000\\ a+\epsilon_{z2}\left(t\right) & 11,000\leq t\leq 21,600 \end{array}\right.$$
  The choice of constants are intended to represent movement of an animal (on the scale of meters), where the movement of an animal is virtually stationary with a sudden dispersal movement to another location. The constants a and d represent the locations where the animal exhibits stationary behavior. The constants b and c describe linear path of the animal during the abrupt movement between locations determined by a and d. The terms $\epsilon_{z1}\left(t\right)$ and $\epsilon_{z2}\left(t\right)$ are normally distributed random variables representing the stochastic component of the animal's path that is not location error. For this simulation $\epsilon_{z1}\left(t\right),\epsilon_{z2}\left(t\right)∼N\left(0,12\right).$
2. Drifting circles movement: Using sine and cosine functions, we can generate drifting circular movement, which could represent a common flight pattern for some vulture (e.g., Cathartidae) species. We use the following equations to represent drifting circular movement in a two‐dimensional space for a 25‐min period with time measured in seconds. We also allow the radius of the circular path to increase and decrease linearly.
$$z_{1}\left(t\right)=r\left(t\right)\sin\left(\phi t\right)+t+\epsilon_{z1}\left(t\right)\;0\leq t\leq 1500$$
$$z_{2}\left(t\right)=r\left(t\right)\cos\left(\phi t\right)+t+\epsilon_{z2}\left(t\right)\;0\leq t\leq 1500$$
  where $r\left(t\right)=\left\{\begin{array}{cc} \mathrm{\alpha t}+\beta & t\leq 750\\ \gamma -\mathrm{\alpha t} & 750\leq t\leq 1500 \end{array}\right.$ and $\epsilon_{z1},\epsilon_{z1}∼N\left(0,12\right).$
  As before, the choice of constants are intended to represent movement of an animal on the scale of meters, where $r\left(t\right)$ is a function that continuously controls the radius of the drifting circles. The constant ϕ controls the periodicity of the drifting circles. Constants α, β, and γ force the radius of the drifting circles to increase and decrease linearly.

With $z\left(t\right)=\left(z_{1}\left(t\right),z_{2}\left(t\right)\right)$ in hand, we can generate telemetry data by randomly sampling some true locations along $z\left(t\right)$ and adding location error to each sampled true location. We allow location error to be nonidentically distributed. For randomly sampled collection of true locations denoted by $\left[z\left(t_{1}\right),\ldots ,z\left(t_{n}\right)\right]$, generated telemetry data can be represented as follows:

$$\begin{array}{c} s_{1}\left(t_{i}\right)=z_{1}\left(t_{i}\right)+\epsilon_{e1}\left(t_{i}\right)\\ s_{2}\left(t_{i}\right)=z_{2}\left(t_{i}\right)+\epsilon_{e2}\left(t_{i}\right) \end{array}$$

where $\epsilon_{e1}\left(t_{i}\right),\epsilon_{2}\left(t_{i}\right)∼\left\{\begin{array}{c} N\left(0,\sigma \right)\;w.p1⁄3\\ \text{Unif}\left(-\xi ,\xi \right)\;w.p1⁄3\\ \mathrm{Tri}\left(-\eta ,\eta \right)\;w.p1⁄3 \end{array}\right.$.

For each true movement path, we considered four scenarios corresponding to combinations of location error (i.e., high and low) and density of recorded locations (i.e., high and low). For each scenario, we generated 500 telemetry datasets, which were then used to assess bias and CPs of estimates of derived quantities from our model.

Density of recorded locations over time is similar to sample size, where having few recorded locations per unit of time or per the monitoring period would indicate smaller sample size telemetry data. For the high location error scenarios, *σ* = 100 m, *ξ* = 120 m, and *η* = 150 m. For the low location error scenarios, *σ* = 10 m, *ξ* = 12 m, and *η* = 15 m. The times $t_{i}$ where i=1,…,n are randomly sampled times from the fine grid of times t used to represent true continuous movement paths, $z\left(t\right)$. As shown above, we allowed the distribution of induced location error to vary randomly among normal, uniform, and triangular distributions. We considered location error to be low when locations are within 30 m of the true location approximately 99% of the time. We considered location error to be high when locations are within 300 m of the true location approximately 99% of the time. More details about location error specification for our simulation can be found in Appendix S2.

For all scenarios with telemetry data generated from the abrupt stepwise movement study, we assessed bias and CPs for estimates of (1) daily displacement, $d_{j}$, on the *j*th day and (2) the proportion of time, $p_{r}$ spent within a radius, r, of roadways. The subscript j denotes the day when abrupt behavior occurred, and r denotes the threshold distance used to estimate proportion of time. For all scenarios with telemetry data generated from the drifting circles movement study, we assessed bias and CPs for estimates of (1) average minutely displacement, $\bar{d}$, over the 25‐min monitoring period and (2) the proportion of time, $p_{r}$, spent within a radius, r, of wind turbines. The true values for all mentioned quantities can be directly calculated from the true movement path.

For each scenario from the abrupt stepwise movement study, we fit a bagged KNNR animal movement model to each of the 500 simulated datasets and estimate $d_{j}$ and $p_{r}$. For each scenario from the drifting circles movement study, we fit a bagged KNNR animal movement model to each of the 500 simulated datasets and estimate $\bar{d}$ and $p_{r}$. For each simulated dataset, we calculate 95% confidence intervals to determine CPs. Details are provided in Appendix S2.

### 2.6 Data Examples

The data examples used in this work are intended to provide an illustration of the implementation of our bagged animal movement model on telemetry data. We considered two data examples in this work: (1) telemetry data collected on a king rail ( *Rallus elegans* ) in Michigan, USA, and (2) telemetry data collected on a mule deer ( *Odocoileus hemionus* ) in Kansas, USA (Combe et al. 2021; Brewer et al. 2023). The king rail data used in Data Example 1 have 37 locations recorded approximately once per day during the 2021 breeding season. This data example illustrates estimation of similar derived quantities to the simulation study on small telemetry data. In Data Example 1, we fit a bagging movement model and estimated the proportion of time an individual spent 15 m or less from wetland edge, which provides pertinent space use information related to predation and human interaction susceptibility. The mule deer data used in Data Example 2 has 6242 locations recorded approximately every hour from March 22, 2020 to October 31, 2020 and every 30 min from November 1, 2020 to December 6, 2020. Data Example 2 illustrates fitting bagged animal movement models for larger telemetry data (e.g., thousands of recorded locations). We provide tutorial code in Appendix S1, to replicate all results in this section.

## 3 Results

### 3.1 Simulation Results

**FIGURE 2:**
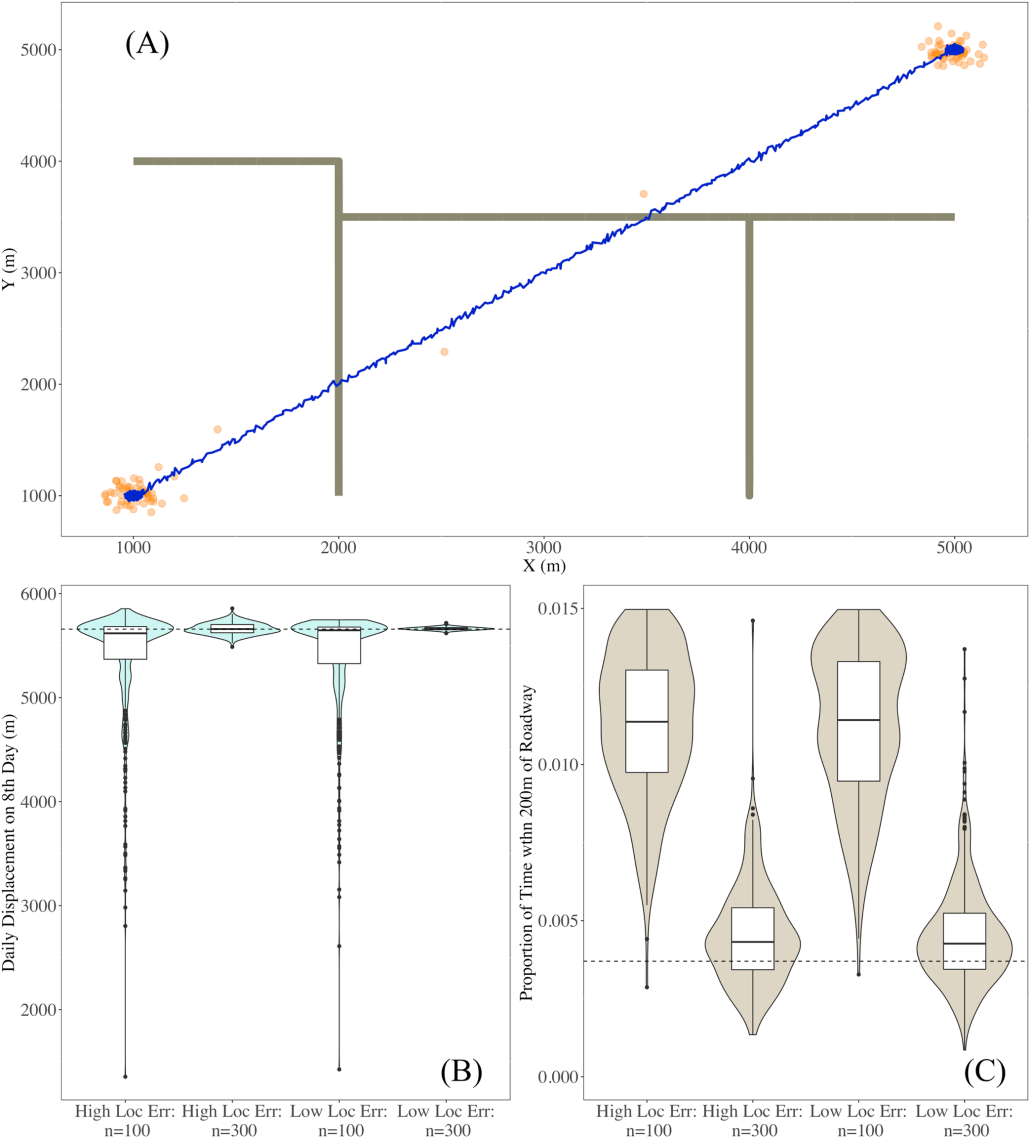
Our visual summary of abrupt stepwise movement simulation study illustrates a hypothetical animal is stationary for most of the experiment except for a brief window of time, where the animal moves rapidly to new location crossing multiple roadways. The blue line in panel (A) illustrates the true continuous movement path of an animal over a 15‐day period where a sudden dispersal movement occurs over a 4‐h period on the eighth day. The orange circles represent one of the simulated telemetry datasets generated from this movement path with high location error induced into the recorded locations. The gray lines represent exact location of roadways in the region. Panels (B) and (C) illustrate the distribution of point estimates for daily displacement on the eighth day, $d_{8}$, and proportion of time spent within 200 m of roadways, $p_{200}$, using our bagged *k*‐nearest neighbor regression animal movement model across 500 simulated telemetry datasets. The black dashed lines in panels (B) and (C) represent the true value for $d_{8}$ and $p_{200}$, respectively.

In our abrupt stepwise simulation study, we generated telemetry data from a known movement path where the animal is stationary for approximately 7 days. On the eighth day, the animal abruptly begins to move with a constant velocity for approximately 7 h until reaching another location. Stationary behavior is resumed at the new location (Figure 2). On the eighth day, the true daily displacement was 5658.5 m (i.e., $d_{8}=5658.5$). Over the course of the 15‐day monitoring period, the proportion of time spent within 200 m of a roadway was 0.0037 (i.e., $p_{200}=0.0037$).

In Table 1, we report the bias and CPs for estimates of both quantities. Using our bagged KNNR movement model, estimates for daily displacement on the eighth day appear to be unbiased for n=300 and bias in estimates increased as the sample size decreased to n=100 (Figure 2B,C). With n=100, there were instances where no recorded locations occurred during the extreme movement, which makes estimates of displacement on this day subject to greater variability and increasing negative bias. For proportion of time spent within 200 m of roadways, estimates have positive bias that increased for lower n. In this simulation study, proportion of time spent near roadways was extremely small (i.e., 0.37% of the monitoring period), occurring during the abrupt movement.

**TABLE 1:** Results from simulation experiment for an abrupt stepwise movement path of a hypothetical animal. The animal is stationary for most of the experiment except for a brief window of time, where the animal moves rapidly to new location crossing multiple roadways. For daily displacement, we focused on bias and coverage probability (CP) for displacement on the eighth day $d_{8}$, which is the day when the animal abruptly switched from stationary to fast directed movement. We estimated the proportion of time spent within 200 m of roadways, $p_{200}$, over the entire 15‐day monitoring period. The sample size, n, is the number of recorded locations in each simulated dataset where n=100 is an average of 6.67 recorded locations per day and n=300 is an average of 20 recorded locations per day. The true daily displacement on the eighth day is *d*_8_ = 5658.5 m. The true proportion of time within 200 m of roadways is $p_{200}=0.0037$. $E\left(\hat{d_{8}}\right)$ represents the expected value of our estimates of daily displacement on the eighth day, $|d_{8}-E\left(\hat{d_{8}}\right)|$ is the bias associated with our estimates, and CP denotes the coverage probability of the true value for daily displacement for a 95% confidence interval. $E\left({\hat{p}}_{200}\right)$ represent the expected value of our estimates for the proportion of time spent within 200 m of roadways, and bias and coverage probability are represent in the same manner of column heading for $d_{8}$.

	Location err.	n	$E\left({\hat{d}}_{8}\right)$	$|d_{8}-E\left({\hat{d}}_{8}\right)|$	CP	$E\left({\hat{p}}_{200}\right)$	$|p_{200}-E\left({\hat{p}}_{200}\right)|$	CP
1	High	300	5666.2	4.0	0.987	0.0044	0.0007	0.974
2	High	100	5406.9	251.5	0.870	0.0147	0.0110	0.546
3	Low	300	5663.4	4.9	0.994	0.0045	0.0008	0.990
4	Low	100	5410.8	247.7	0.873	0.0145	0.0109	0.524

In our drifting circles simulation study, we generated telemetry data from a known movement path where the animal flies through a cluster of wind turbines over a 25‐min period. The radius of the drifting circles increases and then decreases linearly. The average minutely displacement was 96.2 m (i.e., $\bar{d}=96.2$). Over the course of the 25‐min monitoring period, the proportion of time spent within 100 m of a wind turbine was 0.0353 (i.e., $p_{100}=0.0353$). In Table 2, we report the bias and CPs for estimates of both quantities.

**TABLE 2:** Results from simulation experiment for a drifting circles movement path for a hypothetical animal that flies through a region with wind turbines. For average minutely displacement, we summarized bias and coverage probability (CP) for all minutes in the monitoring period. We estimated the proportion of time spent within 100 m of wind turbines, $p_{100}$, over the entire 25‐min monitoring period. The sample size, n, is the number of recorded locations in each simulated dataset where n=30 is an average of 1.2 recorded locations per minute and n=150 is an average of six recorded locations per minute. The true average minutely displacement on the eighth day is $\bar{d}$ = 96.2m. The true proportion of time within 100 m of wind turbines is $p_{100}=0.0353$. $E\left(\hat{\bar{d}}\right)$ represents the expected value of our estimates of average minutely displacement, $|\bar{d}-E\left(\hat{\bar{d}}\right)|$ is the bias associated with our estimates, and CP denotes the coverage probability of the true value for daily displacement for a 95% confidence interval. $E\left({\hat{p}}_{100}\right)$ represent the expected value of our estimates for the proportion of time spent within 100 m of wind turbines, and bias and coverage probability are represent in the same manner of column heading for $\bar{d}$.

	Location err.	n	$E\left(\hat{\bar{d}}\right)$	$|\bar{d}-E\left(\hat{\bar{d}}\right)|$	CP	$E\left({\hat{p}}_{100}\right)$	$|p_{100}-E\left({\hat{p}}_{100}\right)|$	CP
1	High	150	184.1	87.9	0.968	0.061	0.025	0.980
2	High	30	108.4	12.2	0.997	0.068	0.033	0.999
3	Low	150	169.6	73.5	0.977	0.063	0.028	0.950
4	Low	30	122.6	26.5	0.654	0.072	0.037	0.986

**FIGURE 3:**
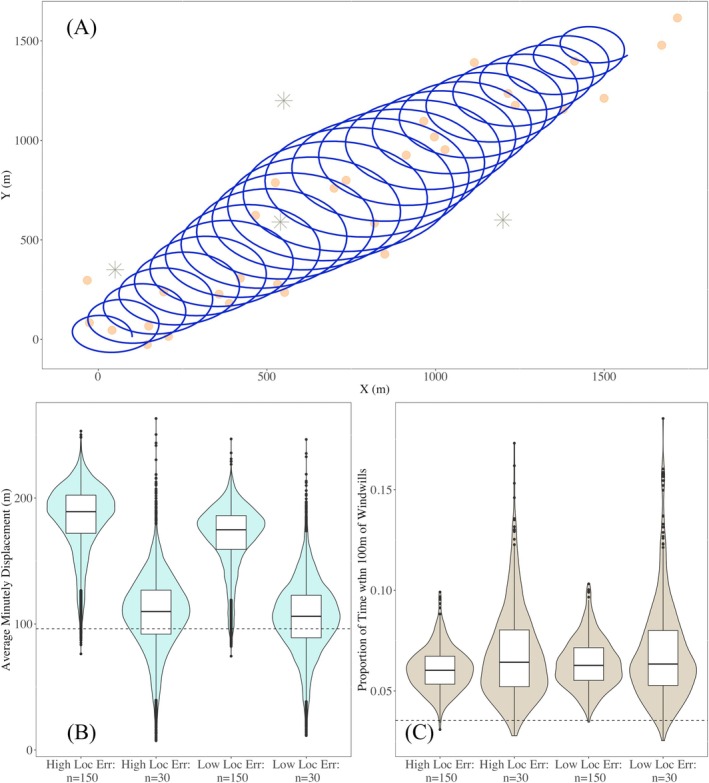
Our visual summary of drifting circle movement simulation study illustrates a hypothetical animal flying through a region with wind turbines. The blue line in panel (A) illustrates the true continuous movement path of an animal presumed to be flying over a 25‐min period. The orange circles represent one of the simulated telemetry datasets generated from this movement path with high location error induced into the recorded locations. The gray asterisk symbols represent the exact locations of wind turbines. Panels (B) and (C) illustrate the distribution of point estimates for average minutely displacement, $\bar{d}$, and proportion of time spent within 100 m of wind turbines, $p_{100}$, using our bagged KNNR animal movement model across 500 simulated telemetry datasets. The black dashed lines in panels (B) and (C) represent the true value for $\bar{d}$ and $p_{100}$, respectively.

We used our bagged KNNR movement model but replaced the standard KNNR algorithm with a weighted KNNR algorithm, which generally provides a smoother representation of complex movement. Weighted KNNR makes predictions using the average of the nearest k recorded locations similarly to standard KNNR where proximity of the neighbors is used to upweight the value of nearer neighbors in the averaging process. The process of switching to another machine learning algorithm only involves adjusting a few lines of code in the functions used to fit the model. We provide more discussion about switching between machine learning algorithms in our discussion section. Across all scenarios, there was a trend toward positive bias and conservative CPs for estimates of both average minutely displacement and proportion of time spent within 100 m of wind turbines (Figure 3B,C).

### 3.2 Data Examples 1 and 2

**FIGURE 4:**
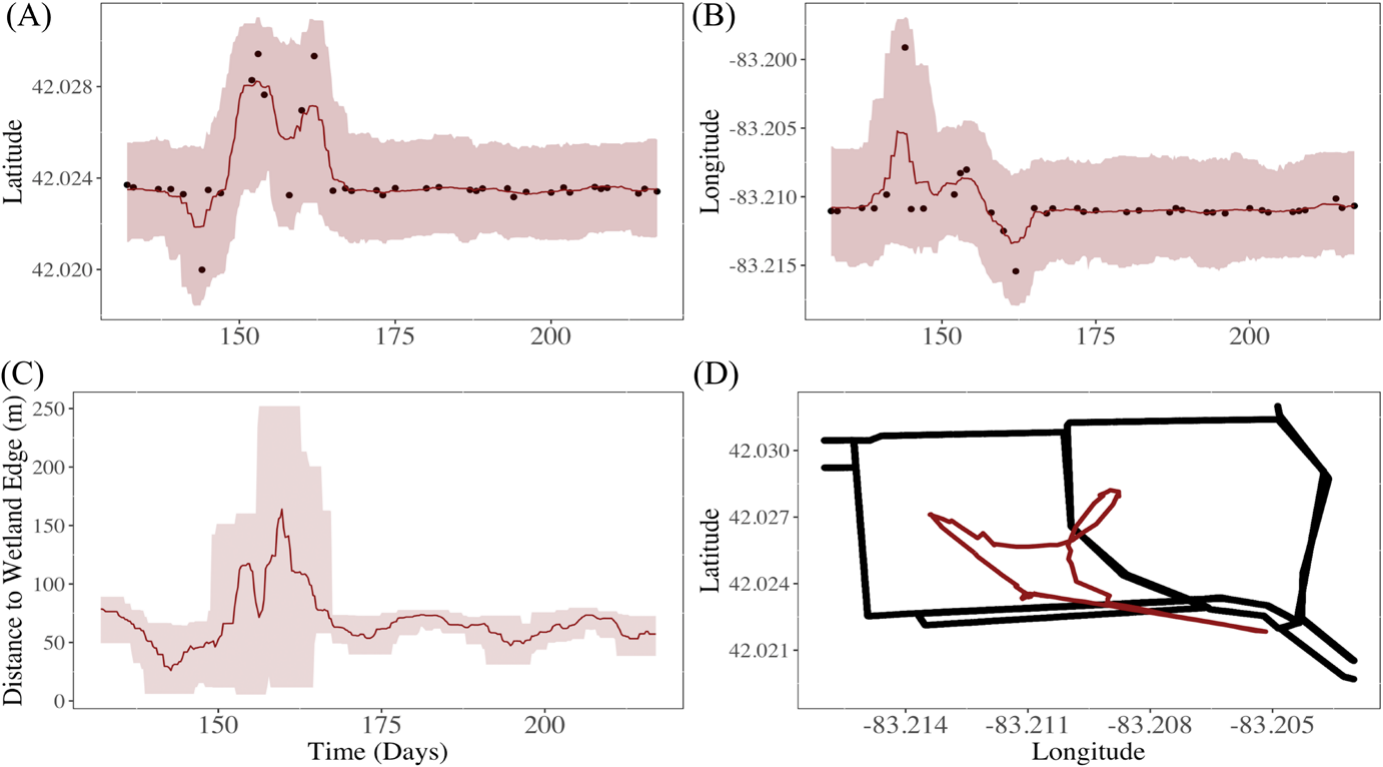
We illustrate a bagging *k*‐nearest neighbor regression animal movement model for king rail ( *Rallus elegans* ) telemetry data for the breeding season. In panels (A) and (B), the black dots represent telemetry data, the expected location of the king rail is depicted as dark red lines and shaded areas represent the predictive distribution for location at any time. Panel (C) illustrates the estimation of the king rail's distance to wetland edge at any time, where the 15 m threshold is represented by a dashed black line. The wetland edge is depicted as dark black lines in panel (D) with the expected location (from panels A and B) overlaid as a dark red line. Distance to wetland edge is used to estimate the proportion of time spent within 15 m of wetland edge.

In Figure 4, we illustrate a bagged KNNR movement model fit to a single king rail's telemetry data and the process of estimating the proportion of time spent within 15 m of the wetland edge. Figure 4A,B depict the 95% prediction intervals for the location of the focal animal at any time with respect to latitude and longitude. In Figure 4C, we show the estimated distance from wetland edge at any time during the monitoring period. The shaded area illustrates the prediction interval from the predictive distribution of distance from wetland edge over time. The predictive distribution of distance from wetland edge is a transformation of the distribution of location using a wetland edge shape‐file to determine the shortest distance from location of an animal to the wetland edge at any specified time. The dotted horizontal line in Figure 4C represents the 15‐m threshold used to estimate the proportion of time spent within 15 m or less of the wetland edge. The proportion of time spent within 15 m or less of wetland edge has a distribution resulting from an indicator function transformation of the distribution for the distance from wetland edge (Horne et al. 2007; Whetten et al. 2024). The estimated proportion of time spent 15 m from wetland edge is 0.039 with a 95% prediction interval estimate of $\left(0.000,0.125\right)$.

**FIGURE 5:**
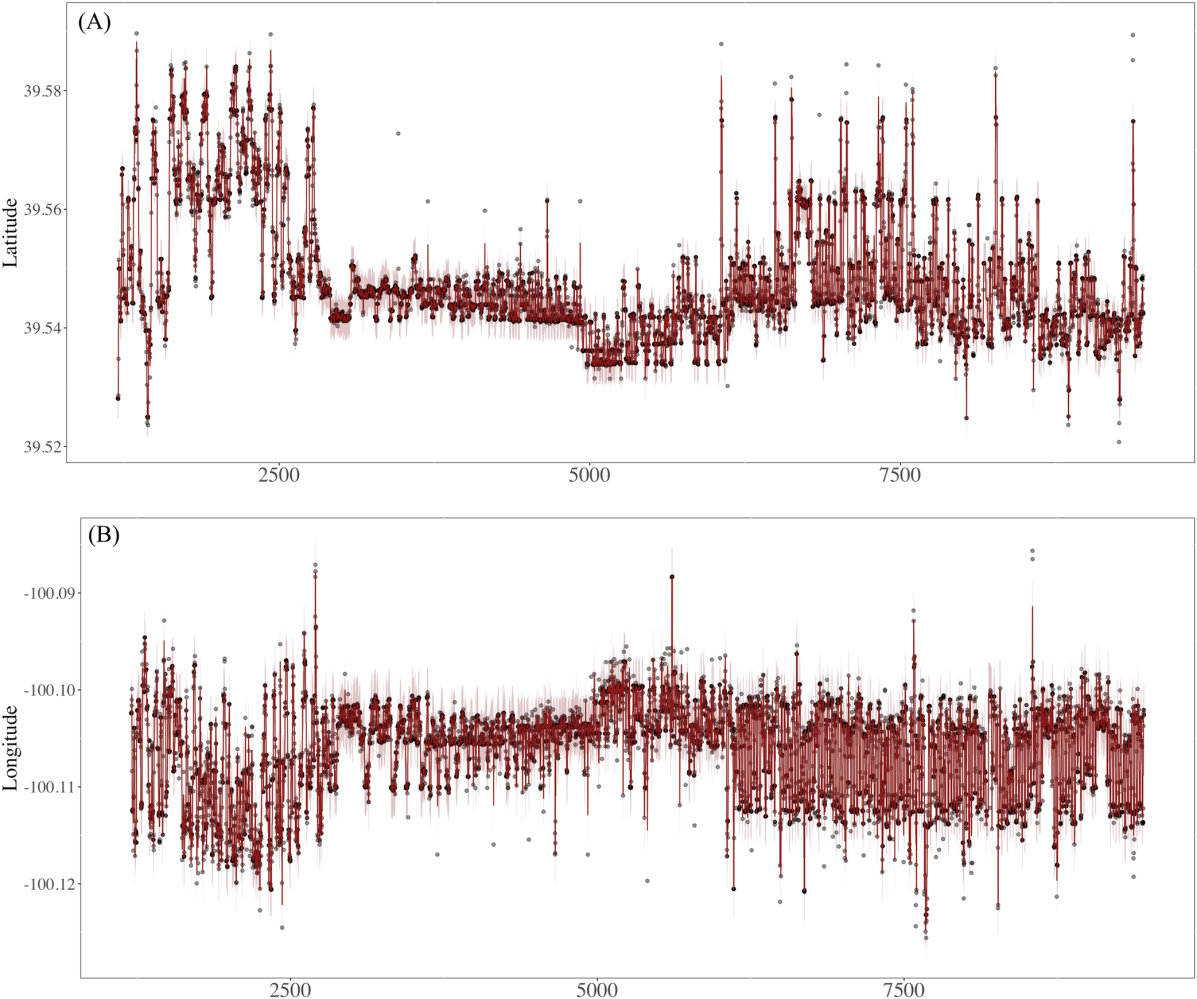
We illustrate a bagged *k*‐nearest neighbor regression animal movement model for mule deer ( *Odocoileus hemionus* ) telemetry data for most of 2020. In panels (A) and (B), the black dots represent telemetry data, the expected location of the mule deer is depicted as a dark red line, and shaded areas represent the predictive distribution for location at any time. This mule deer's telemetry data has 6242 recorded locations. This data example is intended to illustrate the fitting a bagging machine learning movement model to larger telemetry data.

In Figure 5, we illustrate a bagged KNNR movement model fit to a mule deer's telemetry data. Figure 5 depicts the 95% prediction intervals for the location of an animal at any time with respect to latitude and longitude. This example illustrates the use of our model for telemetry data with thousands of recorded locations and hourly recorded locations. This is a substantial increase in size and resolution of telemetry data relative to Data Example 1, which illustrated the use of our model with less than 100 daily recorded locations.

## 4 Discussion

Efforts to improve quality of insights gained from data are hinged on model conceptualization (i.e., ideation of a model before fitting to data) and implementation (James et al. 2013). Model ideation and implementation are inherently application‐dependent and subjective to user training, available resources, and prioritization of various model characteristics. However, there are other practical model characteristics, such as ease of use (relating to model respecification and reprogramming), that may also influence model ideation and implementation.

Our framework for bagging simple machine learning animal movement models builds upon methods from a mainstream discipline with a large potential audience (James et al. 2013). This framework reduces effort required by users in the model specification process. This can allow researchers to focus their energy on formulation and estimation of important derived quantities (Rieber et al. 2024). With our focus turned to derived quantities, we urge readers to carefully consider the use of our framework based on the telemetry data and derived quantities of interest. There are certainly instances where a bespoke Bayesian hierarchical animal movement model will be more ideal (e.g., Brost et al. 2015). We encourage readers to consider the large volume of existing literature on bespoke mechanistic models for animal movement data. Most, if not all, of these choices will require an increasingly complex model with more traditional and computational assumptions.

In our first simulation study, we have shown that it is possible to acquire apparently unbiased estimates of some common derived quantities relating to abrupt and rare movement events, even in the presence of high location error. We expect small sample size bias when the density of recorded locations is decreased substantially. In our second simulation study, we show that there are challenges in acquiring unbiased estimates of similar derived quantities when the sample size of telemetry data are too small relative to the scale and complexity of movement that we are interested in modeling (e.g., Figure 3A). However, in this scenario, CPs for confidence intervals from our model are exceptionally conservative in these less ideal circumstances. In most scenarios in the second study, the true value for each derived quantity is covered 95%–99.9% of the time by a 95% confidence interval (Table 2).

In summary, there are a number of qualitative advantages and disadvantages of our bagged KNN movement relative to general attributes of mechanistic movement models (e.g., state‐space and Bayesian hierarchical movement models). Generally, state‐space models are constructed using several assumptions tied to attributes and autocorrelation of hidden state processes within the model. For Bayesian hierarchical models, there are also several assumptions relating to the data, process, and parameter models. For models fit with MCMC routines, there are further assumptions (e.g., chain convergence, mixing rate, ergodicity) that need to be considered. Generally speaking, our bagged KNN animal movement model alleviates these challenges. There are no traditional model assumptions, and KNN is a computationally stable machine learning algorithm that primarily requires that distances between observations in the data can be computed. Our bagged KNN movement model is flexible enough to model complex and potentially abrupt animal movement patterns. Further, it is convenient to switch between an array of machine learning algorithms within the bagged movement model.

However, it is important to note the limitations of this approach. There are likely some derived quantities that are better estimated using a Bayesian hierarchical animal movement model. There are likely scenarios where an animal movement model that explicitly specifies the location error structure of recorded locations will result in more accurate inference. There may be instances where directly estimating a parameter of interest within a Bayesian hierarchical model is a better choice than estimating a derived quantity. Additionally, although switching between machine learning algorithms is relatively convenient, more complicated machine learning algorithms can inherently increase assumptions and model fitting challenges. As examples, regression trees have stricter assumptions regarding sufficient sample size and node identifiability, and support vector regression has an array of assumptions (e.g., linear separability, margin maximization, influence of outliers on the decision boundary, kernel function choice). Our bagged KNN movement model arguably minimizes model assumptions within this framework of bagged movement models. Overall, we believe that the limitations of this approach will not outweigh the advantages for many researchers.

We believe that bagging machine learning models is an exceptional starting place for model selection and there may be instances where temporal resolution of the data, structure and magnitude of location error may require bespoke mechanistic animal movement models (Fleming et al. 2020). Expanding upon this, our simulation study provides evidence that even in the presence of high location error, our model may be appropriate for estimating some derived quantities (e.g., displacement per unit of time and proportion of time spent in a state). Based on our simulation study, it appears that the bagged KNNR movement model has reasonable frequentist properties even when grossly misspecified with respect to the presence of location error. While there is no theoretical guarantee that this property will hold for all cases, it appears to in our study. We do, however, recommend further simulation experiments. For coarser derived quantities, such as daily displacement or proportion of time spent in region X, it appears that a bagged KNNR movement model provides good estimates. Derived quantities such as proportion of time can be particularly useful for agricultural and engineering applications where the magnitude of wildlife interaction (i.e., usage) of a region provides critical information for ethical anthropogenic alterations to the landscape. Some derived quantities that are more spatially refined descriptions of movement may be better estimated by mechanistic movement models. If location error is determined to be nonneglible, more is known about the error structure and tracking device, and derived quantities of interest have not been assessed through simulation (as shown in this work), we encourage readers to follow current guidelines for model selection (Fleming et al. 2020).

## 5 Conclusion

Our deliberate use of simple bagged models, such as KNNR, is an effort to enable scientists and engineers to obtain statistical inference from telemetry data. There are few adjustments required by the user (e.g., the number of nearest neighbors parameter). Users can program most of the model in a few lines of code. Conceptually, bagged machine learning models only require users to have an introductory knowledge of simple machine learning and bootstrap sampling procedures. Bagged machine learning animal movement models can model complex relationships between time and location and support valid statistical inference about the distribution of animal location at any time. Given the broad interdisciplinary acceptance and utilization of machine learning, we believe that our effort to democratize animal movement modeling provides increased support to researchers with a need to analyze telemetry data.

## Author Contributions

**Andrew B. Whetten:** conceptualization (lead), formal analysis (lead), investigation (lead), methodology (lead), project administration (equal), resources (lead), software (lead), validation (lead), visualization (lead), writing – original draft (lead), writing – review and editing (equal). **Trevor J. Hefley:** conceptualization (supporting), methodology (supporting), supervision (equal), validation (supporting), writing – original draft (supporting), writing – review and editing (equal). **David A. Haukos:** data curation (lead), funding acquisition (lead), project administration (lead), supervision (equal), writing – review and editing (equal). **Dustin E. Brewer:** data curation (lead), writing – review and editing (supporting).

## Conflicts of Interest

The authors declare no conflicts of interest.

## Supporting information


**Appendices S1–S13:** ece372060‐sup‐0001‐AppendicesS1‐S13.zip.

## Data Availability

Mule deer and king rail data used in the data examples illustrated in this manuscript are included with submission material for review.
