# Supplementary material for: Simple Bagged Movement Models for Telemetry Data
Source: Ecol Evol. 2025 Sep 7;15(9):e72060. doi: 10.1002/ece3.72060 (PMC12414728; doi:10.1002/ece3.72060)
Supplement: Supplementary file 1 — Appendices S1–S13: ece372060‐sup‐0001‐AppendicesS1‐S13.zip. [file ECE3-15-e72060-s001.zip › ece372060-sup-0001-AppendixS1.pdf]

Appendix 1

Andrew B. Whetten, Ph.D.

Contents

Section 1: General Overview . . . 1
Section 2: A 1D Animal Telemetry Data Problem . . . 1
Bagging Linear Regression . . . 2
Bagging ML Animal Movement Model . . . 5
Section 3: Overview of Part II . . . 9
Michigan King Rail Telemetry Data Example . . . 10
Mule Deer Telemetry Data in Kansas . . . 21

Section 1: General Overview

The primary purpose of this tutorial is to walk readers through a simple one-dimensional telemetry data example where we implement a bagging machine learning movement model. The first half of the tutorial provides code and thorough comments intended to support user understanding bagging, and how a prediction interval for animal location can be estimated from a bagging movement model. The latter half of the tutorial illustrates basic data preparation and model fitting for two telemetry datasets, which are used in Data Examples 2 and 3 in the main manuscript.

Readers will benefit from having an introductory familiarity with linear regression, supervised machine learning, bootstrap sampling, and telemetry data.

Section 2: A 1D Animal Telemetry Data Problem

We are going to manually create a simple telemetry dataset with 10 recorded locations. The variable x1 will be the time variable, and y1 will be the location in a 1D space.

```
# Dummy Data
  x1 <- 1:10
  y1 <- c(5,3,1,2,6,8,7,7,6,7)
  plot(x1, y1, xlab = "Time", ylab = "Location")
```

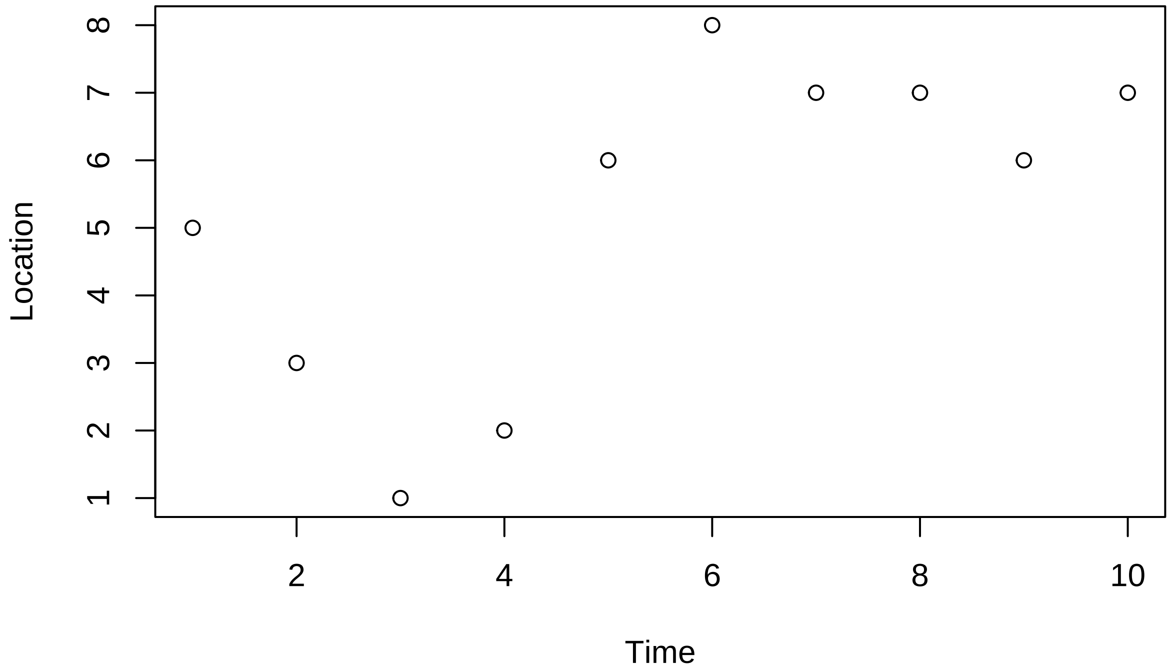


```
df_ex <- as.data.frame(cbind(x1, y1))

# Some "Test Data"
new_dat <- as.data.frame(cbind(seq(1,10, length.out=100)))
names(new_dat) <- "x1"
```

Bagging Linear Regression

Before we the most commonly used machine learning algorithms, such as KNN regression or regression trees, we will fit a simple linear regression and create a bagged linear regression model.

Below is our 1D bagging animal telemetry model using linear regression.

```
  # matrix to store test data predictions for each bootstrap sample
  # just doing B=1000
  mat_pred <- matrix(data=NA, nrow = 1000, ncol=100)
  for (i in 1:1000) {
    # sample from the times in x1 (which are just the numbers 1-10)
    # Note this is bagging since we are sampling 10 times from a dataset
    # with 10 observations
    samp_temp <- sample(1:10, size =10, replace=TRUE)
    # create a temporary data set with these bootstrap samples
    # this is the in-bag data
    df_ex_temp <- df_ex[samp_temp,]
    # fit a simple linear regression for the bagged data!
    lm_temp <- lm(data = df_ex_temp, y1~x1)
    # store predictions from this model for the test data
    mat_pred[i,]  <- predict(lm_temp, newdata = new_dat)
  }
plot(x1, y1, xlab = "Time", ylab = "Location")
lines(new_dat$x1, colMeans(mat_pred), col = "red")
```

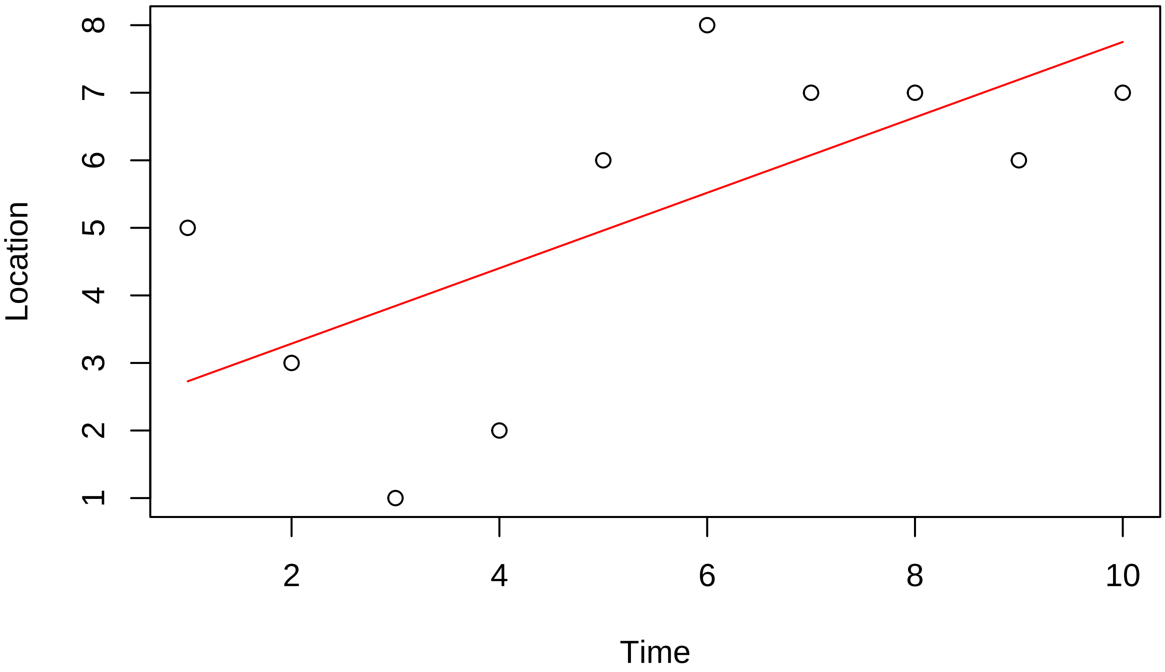

The above code creates a bagged linear regression model, but it only stores predictions of locations for the test data. (In the previous plot, readers should note that we fit a simple linear regression for location with respect to time.) The above code is only intended to be used to familiarize the reader with how bagging models work in their simplest form. The below code is a full bagging linear regression model that estimates confidence intervals for the expected value of location and prediction intervals for the location of an animal at any time.

```
# Storage matrices
# Note that we are fitting a bagged linear regression model using 1000
# bootstrapped lm models. This is why these matrices have 1000 rows.
# Need one for the test data predictions
mat_pred <- matrix(data=NA, nrow = 1000, ncol=100)
# You may want one for predictions on the training data
# this is nice for showing prediction intervals
mat_pred_train <- matrix(data=NA, nrow = 1000, ncol=10)
# Need to store predictions for OOB data! Super important for
# obtaining valid prediction intervals
mat_oob <- matrix(data=NA, nrow = 1000, ncol=10)
# Need to store residual/error for OOB data.
mat_oob_res <- matrix(data=NA, nrow = 1000, ncol=10)
# May want to store residuals for OOB as a vector
# Leaving this in here for now :)

var_vt <- as.numeric()
for (i in 1:1000) {
  # the time (training) data
  x1_temp <- x1
  train_dat <- as.data.frame(x1_temp)
  names(train_dat) <- "x1"
  # get the in-bag and oob samples
  samp_inbag <- sample(x1_temp, size =10, replace=TRUE)
  samp_oob <- x1_temp[x1_temp %in% samp_inbag ==FALSE]
  # in-bag data as df for bagged model
  df_ex_temp <- df_ex[samp_inbag,]
```

```r
  #oob data as df
  oob_dat <- as.data.frame(samp_oob)
  names(oob_dat) <- "x1"
  # in-bag model
  lm_temp <- lm(data = df_ex_temp, y1~x1)
  # get residuals for oob data
  oob_pred_temp <- predict(lm_temp, newdata = oob_dat)
  mat_oob[i, samp_oob] <- oob_pred_temp
  res_temp <- df_ex$y1[x1_temp %in% samp_inbag ==FALSE] - oob_pred_temp
  mat_oob_res[i, samp_oob] <- res_temp
  var_vt <- c(var_vt, var(res_temp))
  # predictions for time grid/test data times
  mat_pred_train[i,] <- predict(lm_temp, newdata = train_dat)
  mat_pred[i,]  <- predict(lm_temp, newdata = new_dat)
}
```

```r
# We can look at the distribution of the residual at some time of interest
# Some points the residual will look good (centered at zero with low variance)
# and some will not.
# For location 1
# hist(mat_oob_res[,1])
# For location 8
# hist(mat_oob_res[,8])

# We can looking at the variance in the residual over time.
# for (i in 1:10) {
#   print(var(mat_oob_res[,i], na.rm = TRUE))
# }

# Construct Prediction Intervals
mat_pred_int <- matrix(data = NA, nrow=10, ncol=2)
for (i in 1:10) {
  mat_pred_int[i,]<-quantile(mat_oob_res[,i],prob = c(0.025, 0.975), na.rm=TRUE)
}

mat_oob_res_vt <- as.vector(mat_oob_res)
pred_int_net <- quantile(mat_oob_res_vt, prob = c(0.025, 0.975), na.rm=TRUE)
# pred_int_net <- apply(mat_oob_res, 2, sd, na.rm = TRUE)

int <- t(apply(mat_pred,2, FUN = quantile, prob = c(0.025, 0.975)))

# using global residual
# Note: We have also overlaid confidence interval for the expected value
# of location as red lines.
plot(df_ex$x1, df_ex$y1, ylim=c(0,10))
lines(new_dat$x1, colMeans(mat_pred), col = "red")
lines(train_dat$x1,  colMeans(mat_pred_train)+ pred_int_net[1], col = "blue")
lines(train_dat$x1, colMeans(mat_pred_train)+pred_int_net[2], col = "green")
lines(new_dat$x1, int[,2], col ="purple")
lines(new_dat$x1, int[,1], col = "purple")
```

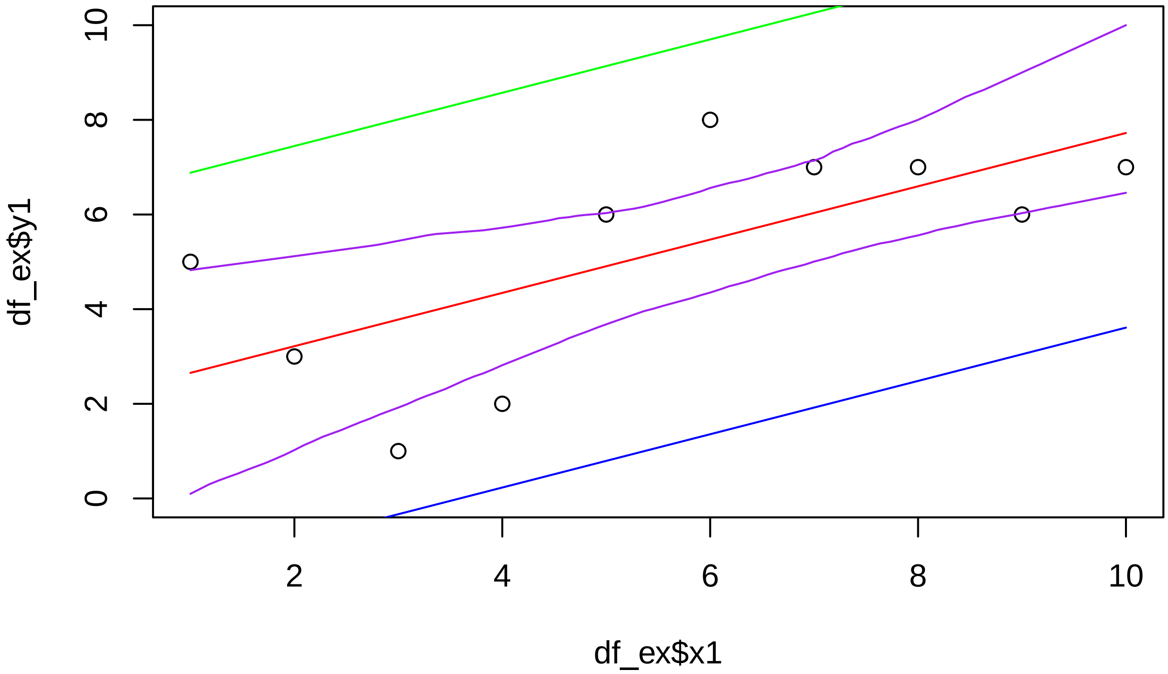


```
  # plot(df_ex$x1, df_ex$y1, ylim=c(0,10))
  # lines(new_dat$x1, colMeans(mat_pred), col = "red")
  # lines(train_dat$x1, colMeans(mat_pred_train) + 1.96*pred_int_net, col = "blue")
  # lines(train_dat$x1, colMeans(mat_pred_train) - 1.96*pred_int_net, col = "green")
  # lines(new_dat$x1, int[,2], col ="purple")
  # lines(new_dat$x1, int[,1], col = "purple")
```

Bagging ML Animal Movement Model

In reference to previous figures, readers familiar with animal movement should see that simple linear regression can be too restrictive to appropriately model animal movement in many scenarios. Simple linear regression tries to represent the relationship between time and location using a single slope and intercept parameter. Animal movement can be nonlinear, and, as such, there are more flexible models that we can consider bagging. K-nearest neighbor regression (KNN) is arguably one of the simpler supervised machine learning algorithms we can bag. There are many great resources available to familiarize readers with the use of KNN, and we encourage readers to reference them as needed.

```
  # Download some package for kNN regression
library(FNN)
library(kknn)
# Create needed storage matrices for model
mat_pred <- matrix(data=NA, nrow = 1000, ncol=100)
mat_pred_train <- matrix(data=NA, nrow = 1000, ncol=10)
mat_oob <- matrix(data=NA, nrow = 1000, ncol=10)
mat_oob_res <- matrix(data=NA, nrow = 1000, ncol=10)
var_vt <- as.numeric()

set.seed(2025)

for (i in 1:1000) {

    x1_temp <- 1:10
    train_dat <- as.data.frame(x1_temp)
```

```r
names(train_dat) <- "x1"
# get the in-bag and oob samples
samp_inbag <- sample(x1_temp, size =10, replace=TRUE)
samp_oob <- x1_temp[x1_temp %in% samp_inbag ==FALSE]
# in-bag data as df for bagged model
df_ex_temp <- df_ex[samp_inbag,]
#oob data as df
oob_dat <- as.data.frame(samp_oob)
names(oob_dat) <- "x1"
# in-bag model and predictions on OOB data
knn_temp_oob <- knn.reg(train=df_ex_temp[,1],
                    test= oob_dat,
                    y = df_ex_temp$y1,
                    k=2)
# knn_temp_oob <- kknn(df_ex_temp$y1~.,train = df_ex_temp, test=oob_dat,
# k = 2, distance = 2, kernel = "rectangular")
#
#
# prediction onto training data
# note this part is optional and can be commented out
knn_temp_train <- knn.reg(train=df_ex_temp[,1],
                    test= train_dat,
                    y = df_ex_temp$y1,
                    k=2)
# knn_temp_train <- kknn(df_ex_temp$y1~.,train = df_ex_temp, test=df_ex_temp,
# k = 2, distance = 2, kernel = "rectangular")

# prediction onto test data (aka a refined grid of time points)
knn_temp_pred <- knn.reg(train=df_ex_temp[,1],
                    test= new_dat,
                    y = df_ex_temp$y1,
                    k=2)
# knn_temp_pred <- kknn(df_ex_temp$y1~.,train = df_ex_temp, test=new_dat,
# k = 2, distance = 2, kernel = "rectangular")
# get residuals for oob data

oob_pred_temp <- knn_temp_oob$pred
mat_oob[i, samp_oob] <- oob_pred_temp
res_temp <- df_ex$y1[x1_temp %in% samp_inbag ==FALSE] - oob_pred_temp
mat_oob_res[i, samp_oob] <- res_temp
var_vt <- c(var_vt, var(res_temp))


# oob_pred_temp <- knn_temp_oob$fitted.values
# mat_oob[i, samp_oob] <- oob_pred_temp
# res_temp <- df_ex$y1[x1_temp %in% samp_inbag ==FALSE] - oob_pred_temp
# mat_oob_res[i, samp_oob] <- res_temp
# var_vt <- c(var_vt, var(res_temp))
# predictions for time grid/test data times
mat_pred_train[i,] <- knn_temp_train$pred
mat_pred[i,]  <- knn_temp_pred$pred

# mat_pred_train[i,] <- knn_temp_train$fitted.values
```

```r
    # mat_pred[i,]  <- knn_temp_pred$fitted.values
  }



# hist(mat_oob[,1])
# hist(mat_oob[,8])
#
# hist(mat_oob_res[,1])
# hist(mat_oob_res[,8])

for (i in 1:10) {
  print(sd(mat_oob_res[,i], na.rm = TRUE))
}
```

```
## [1] 0.9783954
## [1] 1.497635
## [1] 0.9739331
## [1] 1.868124
## [1] 2.401099
## [1] 0.912537
## [1] 0.5760716
## [1] 0.4677167
## [1] 0.1862663
## [1] 0.4648755
```

```r
mat_pred_int <- matrix(data = NA, nrow=10, ncol=2)
for (i in 1:10) {
  mat_pred_int[i,]<-quantile(mat_oob_res[,i],prob = c(0.025, 0.975), na.rm=TRUE)
}

mat_oob_res_vt <-as.vector(mat_oob_res)
pred_int_net <- quantile(mat_oob_res_vt,prob = c(0.025, 0.975), na.rm=TRUE)
pred_int_net <- apply(mat_oob_res,2,sd, na.rm = TRUE)
# pred_int_net <- apply(mat_oob_res,2,quantile, na.rm = TRUE,prob = c(0.025, 0.975))



int <- t(apply(mat_pred,2, FUN = quantile, prob = c(0.025, 0.975)))


plot(df_ex$x1, df_ex$y1, ylim=c(-2,12))
lines(new_dat$x1, colMeans(mat_pred), col = "red")
lines(train_dat$x1, colMeans(mat_pred_train) +2*pred_int_net*(sqrt(1+1/10)), col = "blue")
lines(train_dat$x1, colMeans(mat_pred_train) - 2*pred_int_net*(sqrt(1+1/10)), col = "green")
lines(new_dat$x1, int[,2], col ="purple")
lines(new_dat$x1, int[,1], col = "purple")
```

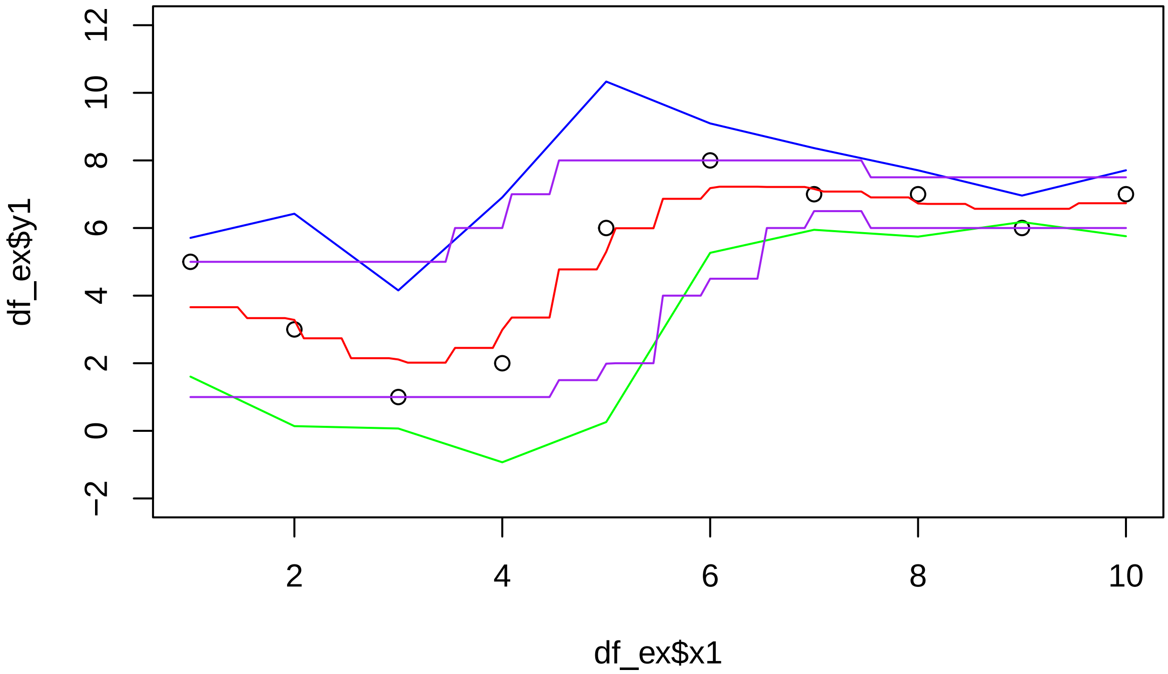


```
# using global residual
# plot(df_ex$x1, df_ex$y1, ylim=c(-2,12))
# lines(new_dat$x1, colMeans(mat_pred), col = "red")
# lines(train_dat$x1, colMeans(mat_pred_train) + pred_int_net[1], col = "blue")
# lines(train_dat$x1, colMeans(mat_pred_train) + pred_int_net[2], col = "green")
# lines(new_dat$x1, int[,2], col ="purple")
# lines(new_dat$x1, int[,1], col = "purple")


sd_knn_res <- sd(mat_oob_res_vt, na.rm = TRUE)

mat_paths <- matrix(data = NA, nrow=1000, ncol=100)

for (i in 1:1000) {
  # use expected value of the animal's path and the information from the
  # prediction interval for each location to generate potential paths
  # of the animal using knn regression
  time_temp <- df_ex$x1
  location_temp <- rnorm(n = length(df_ex$y1), mean = df_ex$y1, sd = pred_int_net)
  knn_temp <- knn.reg(train=time_temp,
                      test= new_dat,
                      y = location_temp,
                      k=2)
  mat_paths[i,] <- knn_temp$pred

}

# for (i in 1:1000) {
#   # use expected value of the animal's path and the information from the
#   # prediction interval for each location to generate potential paths
#   # of the animal using knn regression
#   time_temp <- df_ex$x1
```

```r
#   location_temp <- rnorm(n = length(df_ex$y1), mean = df_ex$y1, sd = sd_knn_res)
#   knn_temp <- knn.reg(train=time_temp,
#                                test= new_dat,
#                                y = location_temp,
#                                k=2)
#   mat_paths[i,] <- knn_temp$pred
#
# }

library(scales)
plot(x1, y1, ylim = c(0,12))
lines(new_dat$x1, colMeans(mat_paths), col ='red')
for (i in 1:1000) {
  lines(new_dat$x1, mat_paths[i,], col=alpha(rgb(0,0,0), 0.08))
}
points(x1, y1, ylim = c(0,12))
lines(new_dat$x1, colMeans(mat_paths), col ='red')
```

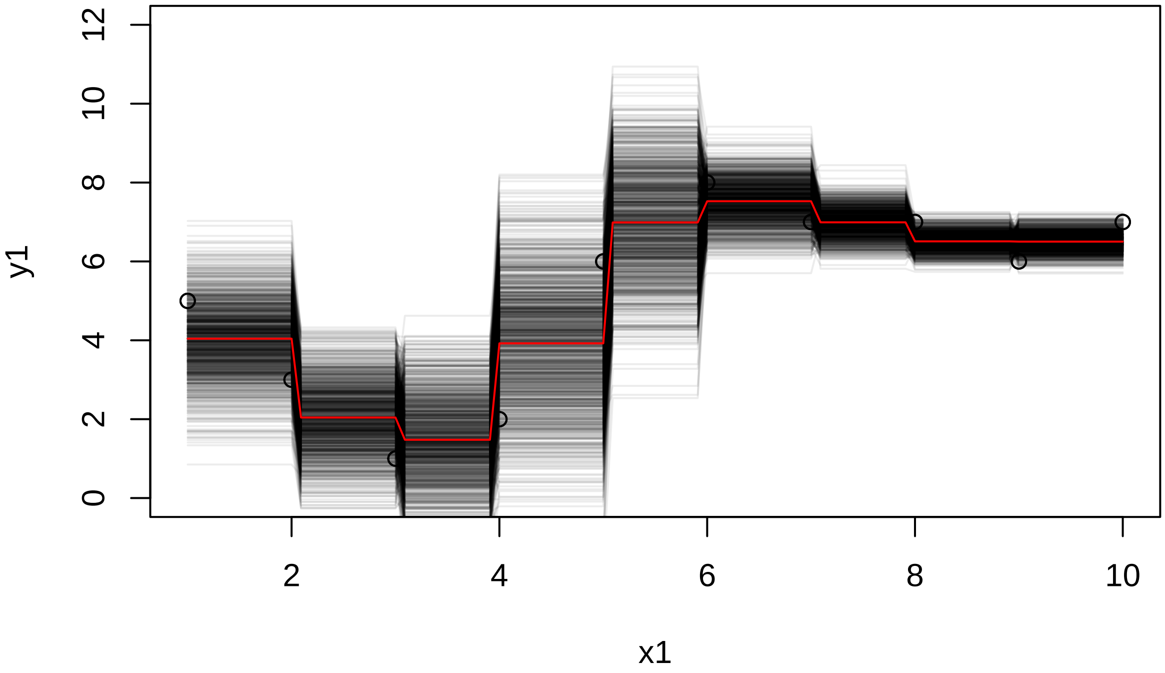

Section 3: Overview of Part II

Towards practical application of bagging animal movement models, we illustrate use of bagging machine learning animal movement models on two data examples. In these data examples, we illustrate data preparation and use of a bagging movement model function that can be used in practice to fit a bagged KNN animal movement model to telemetry data. The function, labeled as knn_bag_move(), has a few practical features worth noting. The function is setup to fit separate models to any number of animals. The computation times for each animal's model are outputted in real-time. The telemetry data for each animal must be stored as a separate object (data frame) in a list object. Although we have not found it necessary, the function can be setup to fit models using parallel computing which can decrease run times. If you are working with telemetry data with more than 20,000 locations per animal and perhaps >500 animals, contact Andrew Whetten for a parallelized version of the knn_bag_move() function.

Michigan King Rail Telemetry Data Example

This king rail telemetry data is a subset of two individuals from a convenience sample of 10 king rail captured and monitored for a single breeding season in 2020 or 2021. Recording locations were taken approximately once per day. We were interested in quantifying the proportion of time king rail spent within 15 meters of the wetland edge. There are a number of concerns related time spent near wetland edge such as localized habitat quality (e.g., higher concentrations of some invasive plant species), susceptibility to predation, and human interaction (e.g., wetland edges are often mowed in this area).

This data example illustrates one way to fit a bagging machine learning movement models in a 2D space and then use information derived from the model to estimate the proportion of time spent within a specified radius of a wetland edge.

```
setwd("/Users/andrewwhetten/Desktop/bagging_movement_models/King_Rail_Example")
# Read in telemetry data
rail <- read.csv("rail_data_MI.csv")
# Checking to see that there are two birds in this dataset
num_birds <-length(table(rail$bird.id))
# Deconstruct KIRA DF into list with 10 elements
list_rail_move <- vector(mode = "list", length = num_birds)
bird_ID <- names(table(rail$bird.id))
for (i in 1:length(list_rail_move)) {
  list_rail_move[[i]] <- rail[rail$bird.id == bird_ID[i],]
}
# Now we have a list with two elements...
# Each element has a single birds telemetry data stored as a separate dataframe
str(list_rail_move)
```

```
## List of 2
##  $ :'data.frame':    37 obs. of  7 variables:
##   ..$ record.id: int [1:37] 1723 1724 1725 1726 1727 1728 1729 1730 1731 1732 ...
##   ..$ bird.id  : num [1:37] 20.2 20.2 20.2 20.2 20.2 ...
##   ..$ latitude : num [1:37] 42 42 42 42 42 ...
##   ..$ longitude: num [1:37] -83.2 -83.2 -83.2 -83.2 -83.2 ...
##   ..$ Date     : int [1:37] 132 133 137 139 141 144 145 147 152 153 ...
##   ..$ year     : int [1:37] 2021 2021 2021 2021 2021 2021 2021 2021 2021 2021 ...
##   ..$ type     : chr [1:37] "kde" "kde" "kde" "kde" ...
##  $ :'data.frame':    31 obs. of  7 variables:
##   ..$ record.id: int [1:31] 1760 1761 1762 1763 1764 1765 1766 1767 1768 1769 ...
##   ..$ bird.id  : num [1:31] 332 332 332 332 332 ...
##   ..$ latitude : num [1:31] 41.5 41.5 41.5 41.5 41.5 ...
##   ..$ longitude: num [1:31] -83 -83 -83 -83 -83 ...
##   ..$ Date     : int [1:31] 142 143 145 147 151 153 155 157 160 163 ...
##   ..$ year     : int [1:31] 2020 2020 2020 2020 2020 2020 2020 2020 2020 2020 ...
##   ..$ type     : chr [1:31] "kde" "kde" "kde" "kde" ...
```

```
# head(list_rail_move[[1]])
# head(list_rail_move[[2]])

# Some other formatting.. nothing exciting
# (1) Call time/date column "num_time"... this is done because for other
# telemetry data, we will likely be converting a date to some form of numeric
# time.
#(2) Captilize Latitude and Longitude. If you are a clever/diligent programmer
# or are doing more than writing a tutorial, you can find a way to avoid this :)
```

```r
names(list_rail_move[[1]]) <- c("record.id", "ID", "Latitude", "Longitude",
                                "num_time", "year", "type")
names(list_rail_move[[2]]) <- c("record.id", "ID", "Latitude", "Longitude",
                                "num_time", "year", "type")
```

KKN Bagging Model Movement

```r
# kNN package
library(FNN)
library(geosphere)

# bootstrap sample of time grid
knn_bag_move <- function(list_move, bag_samps = 500, pred_time_incr =0.5,
                         rand_k = FALSE, k = 2, reweight = FALSE){

  # list_move  = list_rail_move

  list_return <- vector(length = length(list_move), mode = "list")
  list_in_bag <- vector(length = length(bag_samps), mode = "list")
  list_oob <- vector(length = length(bag_samps), mode = "list")

  for (j in 1:length(list_move)) {
    # track computation time by animal
    startTime <- Sys.time()
    # remove missing values
    del <- which(is.na(list_move[[j]]$Latitude))
    if(length(del) !=0){list_move[[j]] <- list_move[[j]][-del,]}

    num_time <- seq(floor(min(list_move[[j]]$num_time)),
                    ceiling(max(list_move[[j]]$num_time)),
                    by = pred_time_incr)
    # Create storage for bootstrap samples and estimated trajectory
    temp_mat_boot <- matrix(nrow = length(list_move[[j]]$num_time),
                            ncol = bag_samps)
    temp_mat_lat <- matrix(nrow = length(num_time), ncol = bag_samps)
    temp_mat_lon <- matrix(nrow = length(num_time), ncol = bag_samps)

    # list_store <- vector(length = 5, mode = "list")
    list_store <- list(boot_t = matrix(),
                       boot_lat = matrix(),
                       boot_lon = matrix(),
                       num_time = as.numeric(),
                       bird_ID = as.character(),
                       path_lat = matrix(),
                       path_lon = matrix()
                       )

    # list_store[[1]] <- temp_mat_boot
    # list_store[[2]] <- temp_mat_lat
    # list_store[[3]] <- temp_mat_lon
    # list_store[[4]] <- num_time
    # list_store[[5]] <- list_move[[j]]$ID
    # list_store[[6]] <- mat_paths_lat
    # list_store[[7]] <- mat_paths_lon
```

```
mat_oob_res_lat <- matrix(data = NA, ncol= dim(list_move[[j]])[1], nrow=bag_samps)
mat_oob_res_lon <- matrix(data = NA, ncol= dim(list_move[[j]])[1], nrow=bag_samps)
for (i in 1:bag_samps) {

  if(rand_k == TRUE){
    k <- sample(2:6, size=1)
  }


  # Bootstrap sample of time (i.e. the only predictor)

  if(reweight ==TRUE){

    dist_step_temp <-  distm(cbind(list_move[[j]]$Longitude,
                                   list_move[[j]]$Latitude),
                             cbind(list_move[[j]]$Longitude,
                                   list_move[[j]]$Latitude))
    # dist_step_temp <-  distm(list_move[[j]][,c(4,3)], list_move[[j]][,c(4,3)])
    dist_step_temp <- colMeans(dist_step_temp)

    frw_wts_temp <- dist_step_temp^0.5/sum(dist_step_temp^0.5)

    x1_temp <- 1:length(list_move[[j]]$num_time)
    samp_inbag <- sample(x1_temp,
                         size =length(list_move[[j]]$num_time),
                         replace=TRUE,
                         prob = frw_wts_temp)
    samp_oob <- x1_temp[x1_temp %in% samp_inbag ==FALSE]
  }


   if(reweight ==FALSE){
    x1_temp <- 1:length(list_move[[j]]$num_time)
    samp_inbag <- sample(x1_temp,
                         size =length(list_move[[j]]$num_time),
                         replace=TRUE)
    samp_oob <- x1_temp[x1_temp %in% samp_inbag ==FALSE]
  }


  # boot_time <- sample(1:length(list_move[[j]]$num_time),
  #                     size = length(list_move[[j]]$num_time),
  #                     replace = TRUE)

  # in-bag data as df for bagged model
  list_in_bag[[i]] <- list_move[[j]][samp_inbag,]
  # df_temp <- list_move[[j]][samp_inbag,]

  #oob data as df
  list_oob[[i]] <- as.data.frame(list_move[[j]]$num_time[samp_oob])
  # oob_dat <- as.data.frame(list_move[[j]]$num_time[samp_oob])
  names(list_oob[[i]]) <- "num_time"
```

```
  # kNN reg for latitude location using boot time to train and
  # num_time as the test data

  # in-bag model used to make OOB predictions
  # this is used to measure residuals for prediction intervals
  # knn_temp_oob_lat <- knn.reg(train=df_temp$num_time,
  #                         test= oob_dat,
  #                         y = df_temp$Latitude,
  #                         k=k)
  knn_temp_oob_lat <- knn.reg(train=list_in_bag[[i]]$num_time,
                          test= list_oob[[i]],
                          y = list_in_bag[[i]]$Latitude,
                          k=k)
  # in-bag model used to make predictions on new time grid (real test data)
  # These can be used to get a distribution for the expected movement path
  # knn_temp_pred_lat <- knn.reg(train=df_temp$num_time,
  #                     test= as.data.frame(num_time),
  #                     y = df_temp$Latitude,
  #                     k=2)
  knn_temp_pred_lat <- knn.reg(train=list_in_bag[[i]]$num_time,
                      test= as.data.frame(num_time),
                      y = list_in_bag[[i]]$Latitude,
                      k=2)



  # kNN reg for longitude location using boot time to train and
  # num_time as the test data
  # in-bag model used to make OOB predictions
  # this is used to measure residuals for prediction intervals
  # knn_temp_oob_lon <- knn.reg(train=df_temp$num_time,
  #                         test= oob_dat,
  #                         y = df_temp$Longitude,
  #                         k=k)
  knn_temp_oob_lon <- knn.reg(train=list_in_bag[[i]]$num_time,
                          test= list_oob[[i]],
                          y = list_in_bag[[i]]$Longitude,
                          k=k)

# in-bag model used to make predictions on new time grid (real test data)
  # These can be used to get a distribution for the expected movement path
  # knn_temp_pred_lon <- knn.reg(train=df_temp$num_time,
  #                     test= as.data.frame(num_time),
  #                     y = df_temp$Longitude,
  #                     k=k)
  knn_temp_pred_lon <- knn.reg(train=list_in_bag[[i]]$num_time,
                      test= as.data.frame(num_time),
                      y = list_in_bag[[i]]$Longitude,
                      k=k)

  # Store the prediction for the test data
  f.hat.lat <- knn_temp_pred_lat$pred
  f.hat.lon <- knn_temp_pred_lon$pred
```

```
    # store record of in-bag times
    temp_mat_boot[,i] <- samp_inbag
    # Store information for distributed of expected movement path
    temp_mat_lat[,i] <- f.hat.lat
    temp_mat_lon[,i] <- f.hat.lon

    # get residuals for oob data
    oob_pred_temp_lat <- knn_temp_oob_lat$pred
    oob_pred_temp_lon <- knn_temp_oob_lon$pred
    # mat_oob_lat[i, samp_oob] <- oob_pred_temp_lat
    # mat_oob_lon[i, samp_oob] <- oob_pred_temp_lon
    res_temp_lat <- list_move[[j]]$Latitude[x1_temp %in%
                                   samp_inbag ==FALSE] - oob_pred_temp_lat
    res_temp_lon <- list_move[[j]]$Longitude[x1_temp %in%
                                    samp_inbag ==FALSE] - oob_pred_temp_lon
    mat_oob_res_lat[i, samp_oob] <- res_temp_lat
    mat_oob_res_lon[i, samp_oob] <- res_temp_lon


    # var_vt <- c(var_vt, var(res_temp))
  }

  pred_int_net_lat <- apply(mat_oob_res_lat,1,sd, na.rm = TRUE)
  pred_int_net_lon <- apply(mat_oob_res_lon,1,sd, na.rm = TRUE)
   # mat_oob_res_vt_lat <-as.vector(mat_oob_res_lat)
   # mat_oob_res_vt_lon <-as.vector(mat_oob_res_lon)
   #
   # sd_knn_res_lat <- sd(mat_oob_res_vt_lat, na.rm = TRUE)
   # sd_knn_res_lon <- sd(mat_oob_res_vt_lon, na.rm = TRUE)

   mat_paths_lat <- matrix(data=NA, ncol= length(num_time), nrow=bag_samps)
   mat_paths_lon <- matrix(data=NA, ncol= length(num_time), nrow=bag_samps)
      for (i in 1:bag_samps) {
        # n=1
        # use expected value of the animal's path and the information from the
        # prediction interval for each location to generate potential paths
        # of the animal using knn regression
        time_temp <- list_in_bag[[i]]$num_time
        location_temp_lat <- rnorm(n = length(list_in_bag[[i]]$Latitude),
                             mean = list_in_bag[[i]]$Latitude, sd = pred_int_net_lat)
        location_temp_lon <- rnorm(n = length(list_in_bag[[i]]$Longitude),
                             mean = list_in_bag[[i]]$Longitude, sd = pred_int_net_lon)
        # location_temp_lat <- rnorm(n = length(df_temp$Latitude),
        #                        mean = df_temp$Latitude, sd = sd_knn_res_lat)
        # location_temp_lon <- rnorm(n = length(df_temp$Longitude),
        #                        mean = df_temp$Longitude, sd = sd_knn_res_lon)
        knn_temp_lat <- knn.reg(train=time_temp,
                            test= as.data.frame(num_time),
                            y = location_temp_lat,
                            k=k)
        knn_temp_lon <- knn.reg(train=time_temp,
                            test= as.data.frame(num_time),
                            y = location_temp_lon ,
```

```
                                    k=k)
          mat_paths_lat[i,] <- knn_temp_lat$pred
          mat_paths_lon[i,] <- knn_temp_lon$pred
        }

      # plot(num_time, colMeans(mat_paths_lat), col = "red", type="l")
      # for (t in 1:100) {
      #   lines(num_time, mat_paths_lat[t,], col = "yellow", type="l")
      # }

    list_store[[1]] <- temp_mat_boot
    list_store[[2]] <- temp_mat_lat
    list_store[[3]] <- temp_mat_lon
    list_store[[4]] <- num_time
    list_store[[5]] <- list_move[[j]]$ID[1]
    list_store[[6]] <- mat_paths_lat
    list_store[[7]] <- mat_paths_lon
    list_return[[j]] <- list_store

    endTime <- Sys.time()
    print(endTime - startTime)
  }

  return(list_return)
}



# Estimate movement models for all deer in the tutorial
# Note: The time to estimate a movement model for each deer
# is printed.


rail_traj_list_knn <- knn_bag_move(list_move  = list_rail_move,
                                   pred_time_incr = 0.25,
                                   reweight = FALSE)
```

```
## Time difference of 1.099801 secs
## Time difference of 0.9052072 secs
```

```
library(ggplot2)
library(gridExtra)
##################################
### Sample Visuals ###############


# generic template for plots
gg_temp <- ggplot() +
  theme(text = element_text(family="Times",size=18),
        plot.title = element_text(size = 18),
        axis.text.x=element_text(size=18),
        panel.background = element_rect(fill = "white", colour = "black"),
        panel.grid.major = element_blank(),
        panel.grid.major.y=element_blank(),
```

```
        legend.position = "none")



# Bird 1 KIRA
f.bar.1lat <- colMeans((rail_traj_list_knn[[1]][[6]]))
f.CI.1lat <- t(apply(rail_traj_list_knn[[1]][[6]],
                     2, FUN = quantile, prob = c(0.025, 0.975)))
gg_kira_1_lat <- gg_temp +
  geom_point(aes(x = num_time,
                 y = Latitude),
             data = list_rail_move[[1]]) +
  geom_line(aes(x=rail_traj_list_knn[[1]][[4]] ,
                y =f.bar.1lat),
            color= "firebrick4") +
  geom_ribbon(aes(x=rail_traj_list_knn[[1]][[4]] ,
                  ymin=f.CI.1lat[,1],
                  ymax=f.CI.1lat[,2]), alpha=0.3, fill= "firebrick4") +
  ylab("Latitude")  + xlab(" ")

f.bar.1lon <- colMeans((rail_traj_list_knn[[1]][[7]]))
f.CI.1lon <- t(apply(rail_traj_list_knn[[1]][[7]],
                     2, FUN = quantile, prob = c(0.025, 0.975)))
gg_kira_1_lon <- gg_temp +
  geom_point(aes(x = num_time,
                 y = Longitude),
             data = list_rail_move[[1]]) +
  geom_line(aes(x=rail_traj_list_knn[[1]][[4]] ,
                y =f.bar.1lon),
            color= "firebrick4") +
  geom_ribbon(aes(x=rail_traj_list_knn[[1]][[4]] ,
                  ymin=f.CI.1lon[,1],
                  ymax=f.CI.1lon[,2]), alpha=0.3, fill= "firebrick4") +
  ylab("Longitude")  + xlab(" ")

grid.arrange(gg_kira_1_lat, gg_kira_1_lon)
```

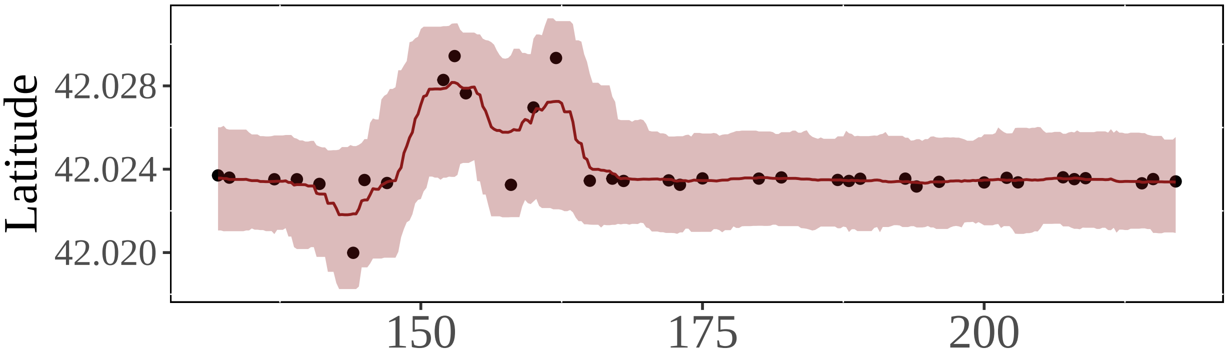

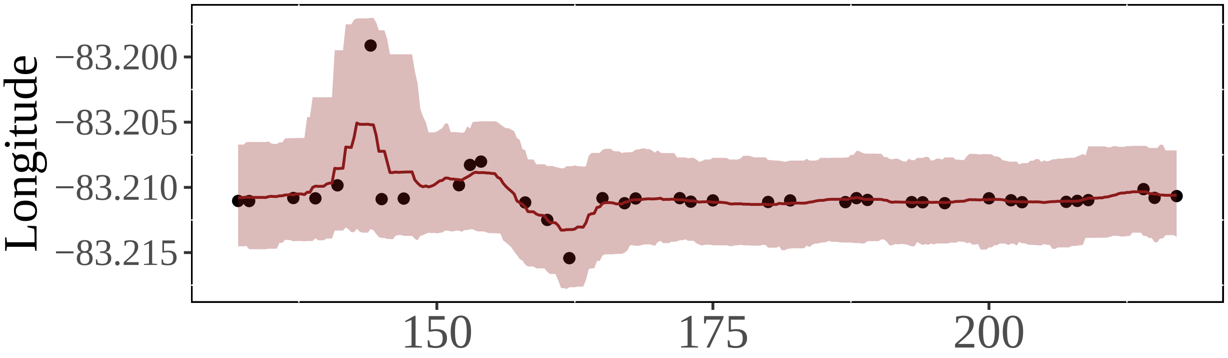


```r
setwd("/Users/andrewwhetten/Desktop/bagging_movement_models/King_Rail_Example/Levees")

# Discretize shapefiles to have 10,000 pts each
library(lwgeom)
```

```
## Linking to liblwgeom 3.0.0beta1 r16016, GEOS 3.11.0, PROJ 9.1.0
```

```r
library(geosphere)
library(FNN)
library(sf)
```

```
## Linking to GEOS 3.11.0, GDAL 3.5.3, PROJ 9.1.0; sf_use_s2() is TRUE

##
## Attaching package: 'sf'

## The following object is masked from 'package:lwgeom':
##
##     st_perimeter
```

```r
shape_wetland <- st_read(dsn = ".", layer = "Levees")
```

```
## Reading layer `Levees' from data source
##   `/Users/andrewwhetten/Desktop/bagging_movement_models/King_Rail_Example/Levees'
##   using driver `ESRI Shapefile'
## Simple feature collection with 30 features and 2 fields
## Geometry type: POLYGON
## Dimension:     XYZ
## Bounding box:  xmin: -83.25491 ymin: 41.40904 xmax: -82.91624 ymax: 42.03672
## z_range:       zmin: 0 zmax: 0
```

```
## Geodetic CRS:  WGS 84
```

```r
shape_wetland <- st_transform(shape_wetland, crs = '+proj=longlat +datum=WGS84')
shape_wetland_100k <- st_sample(st_cast(shape_wetland $geometry,"MULTILINESTRING"), 100000)
```

```
## although coordinates are longitude/latitude, st_sample assumes that they are
## planar
```

```r
shape_wetland_100k <- data.frame(st_coordinates(st_cast(shape_wetland_100k,"MULTIPOINT")))
# plot(shape_wetland_100k$X, shape_wetland_100k$Y, type = "l")

dist_wetland_edge <- function(bird_list, wetland_shp_df){

  # bird_list <- kira_traj_list_knn
  # wetland_shp_df <- shape_020_21_10k
  # i = 1
  # j = 1

  #dist_wetland_edge_list <- vector(length = length(bird_list), mode = "list")

  #for (i in 1:length(bird_list)) {
  dist_mat_temp <- matrix(data = NA, nrow = length(bird_list[[4]]) , ncol = 500)
  #str(bird_list[[1]])
  for (j in 1:500) {
    temp_df <- as.data.frame(cbind(bird_list[[3]][,j], bird_list[[2]][,j]))
    names(temp_df) <- c("X", "Y")
    traj_dim <- dim(temp_df)[1]
    temp_df_merge <- as.data.frame(rbind(temp_df, wetland_shp_df[,1:2]))
    ttl_dim <- dim(temp_df_merge)[1]
    knn_trial <- get.knnx(temp_df_merge[(traj_dim + 1):ttl_dim,],
                          k=1, query = temp_df_merge[1:traj_dim,])

    vt_temp <- (traj_dim + 1):ttl_dim

    nearest_wetland_edge_pts <- temp_df_merge[ vt_temp[knn_trial$nn.index],]
    head(temp_df)
    dist_mat_temp[,j] <- diag(distm(temp_df, nearest_wetland_edge_pts, fun = distHaversine))

    if(j%%1000 == 0){print(j)}
    #str(meep)
    #meep <- distm(coordinates(CP.pts), coordinates(NP.pts), fun = distHaversine)
    #plot(diag(meep))
  }
  #dist_wetland_edge_list[[i]] <- dist_mat_temp

  #}
  return(dist_mat_temp)
  #return(dist_wetland_edge_list)
}

list_dist_wetland_edge <- vector(mode = "list", length = num_birds)
for (i in 1:num_birds) {
  list_dist_wetland_edge[[i]] <- dist_wetland_edge(rail_traj_list_knn[[i]], shape_wetland_100k)
}
```

```r
# dist_wetedge_temp<- dist_wetland_edge(traj_list_knn[[1]], shape_wetland_100k)
# str(dist_wetedge_temp)


# Estimate Proportion of Time an Individual Spent within 15m of the wetland edge

prop_time_near_wtedge <- function(dist_wetedge, threshold = 15.0){
  #dist_wetedge <- dist_wetedge_020_21
  dist_wted <- as.numeric()
  for (i in 1:dim(dist_wetedge)[2]) {
    #i=1
    temp_prop <- sum(dist_wetedge[,i] < threshold)/dim(dist_wetedge)[1]
    dist_wted <- c(dist_wted, temp_prop)
    #tab_temp <- table(dist_wetedge[,1] < threshold)/dim(dist_wetedge)[1]
    #dist_wted <- c(dist_wted, as.numeric(tab_temp[2]))
  }
  return(dist_wted)
}

list_prop_wet_15 <- vector(mode = "list", length = num_birds)
for (i in 1:num_birds) {
  list_prop_wet_15[[i]] <- prop_time_near_wtedge(list_dist_wetland_edge[[i]])
}
str(list_prop_wet_15[1:2])
```

```
## List of 2
##  $ : num [1:500] 0.044 0 0 0.0938 0.1085 ...
##  $ : num [1:500] 0 0.085 0.0935 0.136 0.1728 ...
```

```r
boxplot(list_prop_wet_15[[1]])
```

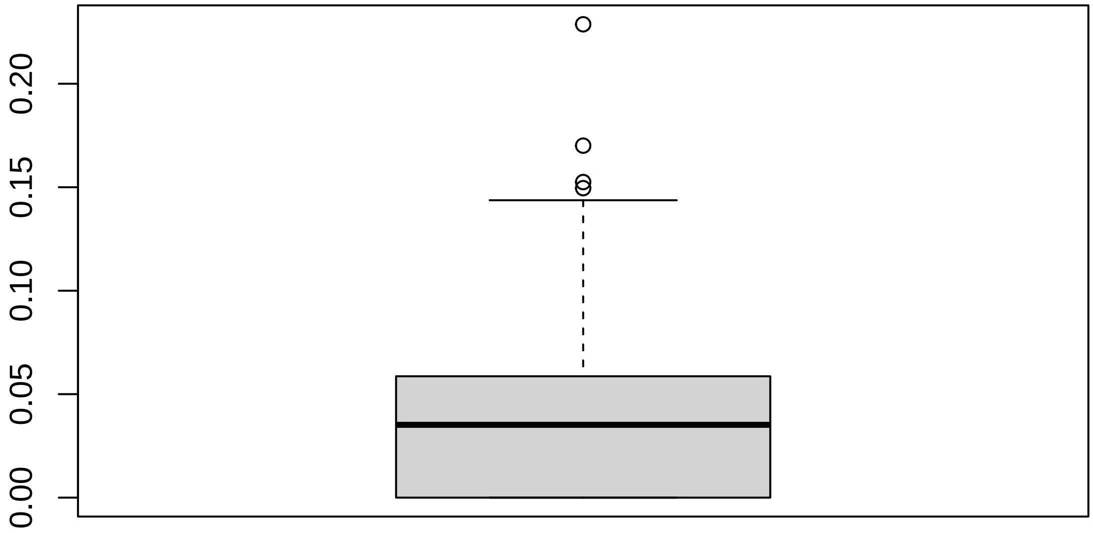


```r
# prop_time_15wet <- as.numeric()
# for (i in 1:num_birds) {
#   prop_time_15wet <- c(prop_time_15wet, mean(list_prop_wet_15[[i]]))
# }

f.bar.wt1 <- rowMeans(list_dist_wetland_edge[[1]])
```

```r
f.CI.wt1 <- t(apply(list_dist_wetland_edge[[1]],
                    1, FUN = quantile, prob = c(0.025, 0.975)))
gg_kira_1_dist <- gg_temp +
  geom_line(aes(x=rail_traj_list_knn[[1]][[4]] ,
                y =f.bar.wt1),
            color= "firebrick4") +
  geom_ribbon(aes(x=rail_traj_list_knn[[1]][[4]] ,
                  ymin=f.CI.wt1[,1],
                  ymax=f.CI.wt1[,2]), alpha=0.2, fill= "firebrick4") +
  ylab("Distance to Wetland Edge (m)")  + xlab("Time (Days)")

gg_kira_1_geo <- gg_temp +
  geom_point(aes(x=shape_wetland_100k$X,
                 y =shape_wetland_100k$Y),
             color= "black") +
  geom_path(aes(x=rowMeans((rail_traj_list_knn[[1]][[3]])),
                y=rowMeans((rail_traj_list_knn[[1]][[2]]))), col = "firebrick4", size = 1.5) +
  ylab("Latitude")  + xlab("Longitude") + ylim(42.017,42.037) + xlim(-83.225, -83.193)
```

```
## Warning: Using `size` aesthetic for lines was deprecated in ggplot2 3.4.0.
## i Please use `linewidth` instead.
## This warning is displayed once every 8 hours.
## Call `lifecycle::last_lifecycle_warnings()` to see where this warning was
## generated.
```

```r
gg_kira_1_geo <- gg_temp +
  geom_point(aes(x=shape_wetland_100k$X,
                 y =shape_wetland_100k$Y),
             color= "black") +
  geom_path(aes(x=rowMeans((rail_traj_list_knn[[1]][[3]])),
                y=rowMeans((rail_traj_list_knn[[1]][[2]]))), col = "firebrick4", size = 1.5) +
  ylab("Latitude")  + xlab("Longitude") + ylim(42.019,42.032) + xlim(-83.216, -83.203)

grid.arrange(gg_kira_1_lat, gg_kira_1_lon, gg_kira_1_dist, gg_kira_1_geo)
```

```
## Warning: Removed 87650 rows containing missing values or values outside the scale range
## (`geom_point()`).
```

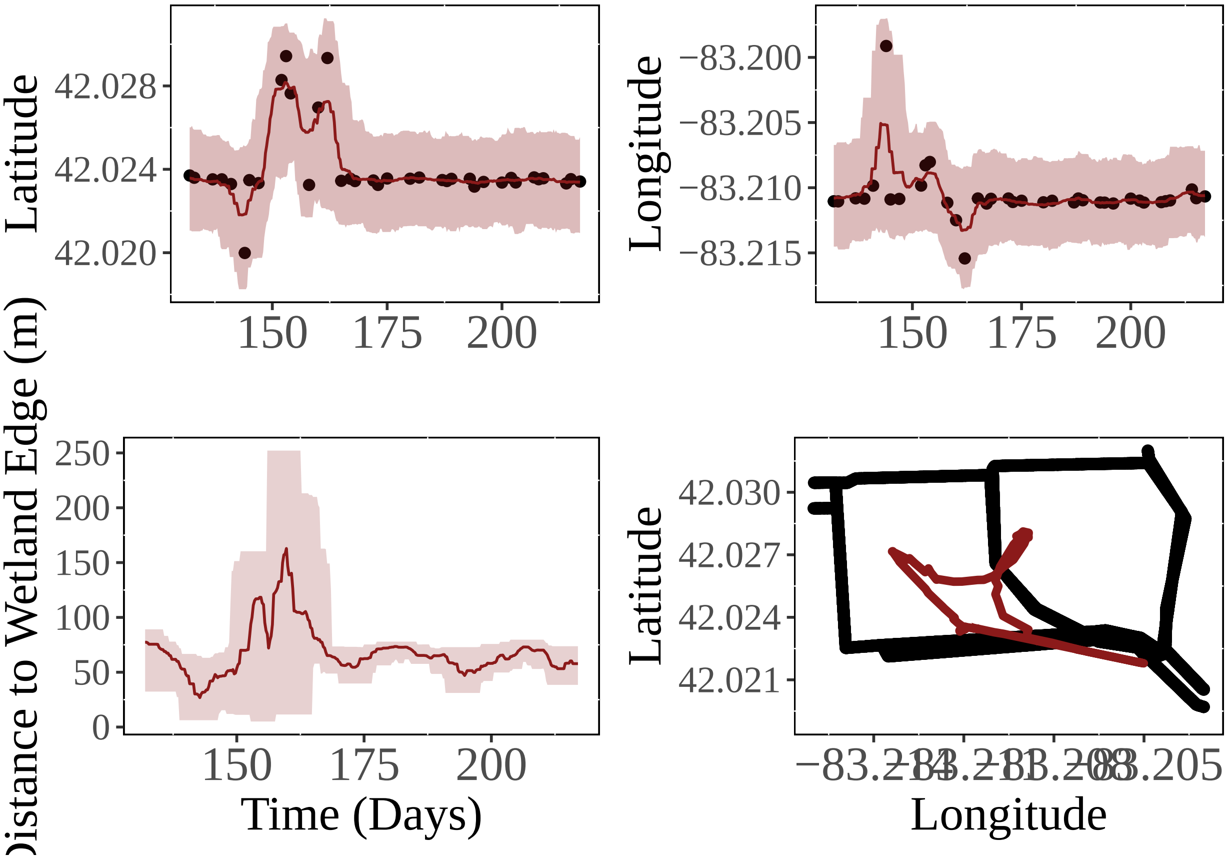

Mule Deer Telemetry Data in Kansas

In this section, we provide another illustration of bagging machine learning movement models for animal telemetry data. In the king rail data example, each bird had approximately 30-50 recorded locations. This data example illustrates the use of our approach on larger telemetry data. We use the telemetry data of two female mule deer monitored in Western Kansas in the same study area in 2020. Each deer has between 6000 to 8000 recorded locations. Recorded locations were taken approximately every hour.

```
# Nice package for working with Date-Time objects
library(lubridate)
```

```
##
## Attaching package: 'lubridate'

## The following objects are masked from 'package:base':
##
##     date, intersect, setdiff, union
```

```
library(readr)
```

```
##
## Attaching package: 'readr'

## The following object is masked from 'package:scales':
##
##     col_factor
```

```
setwd("/Users/andrewwhetten/Desktop/bagging_movement_models/Mule_Deer_Example/")
females_20 <- readr::read_csv(list.files(".", "^mule", full.names = TRUE))
```

```
## Rows: 14401 Columns: 8
```

```
## -- Column specification --------------------------------------------------------
## Delimiter: ","
## chr  (2): LMT_Date, Mort. Status
## dbl  (5): CollarID, Latitude, Longitude, Easting, Northing
## time (1): LMT_Time
##
## i Use `spec()` to retrieve the full column specification for this data.
## i Specify the column types or set `show_col_types = FALSE` to quiet this message.
```

```r
# # Function to read in multiple csv files
# do.call_rbind_read.csv <- function(path, pattern = "*.csv") {
#   # Set your own working directory
#   path = "/Users/andrewwhetten/Desktop/bagging_movement_models/Mule_Deer_Example/"
#   pattern = "*.csv"
#   files = list.files(path, pattern, full.names = TRUE)
#   females_20 <- do.call(rbind, lapply(files,
#                               function(x) read.csv(x,
#                               stringsAsFactors = FALSE,
#                               check.names = F)))
# }
#
# females_20 <- do.call_rbind_read.csv(
#   # select your own working directory or remove the line
#   # of code below
#   path = "/Users/andrewwhetten/Desktop/bagging_movement_models/Mule_Deer_Example/",
#                                     pattern = "*.csv")
# # Re-lable Columns names
# names(females_20) <- c("CollarID", "LMT_Date", "LMT_Time", "Latitude",
#                         "Longitude", "Mort. Status", "Easting", "Northing")
# head(females_20)

# Date-Time formatting
# females_20$Time <- as.numeric(hms(females_20$LMT_Time))
# females_20$Time <- as.character(females_20$LMT_Time)
# females_20$Time <- format(strptime(females_20$Time,
#                                  "%I:%M:%S %p"), "%H:%M:%S")
# females_20$Date <- mdy(females_20$LMT_Date)
# head(females_20)
females_20$t <- paste(females_20$LMT_Date, females_20$LMT_Time)
# females_20$t <- paste(females_20$Date, females_20$Time)
females_20$t <- mdy_hms(females_20$t)
names(females_20) <- c("ID", "LMT_Date", "LMT_Time", "Latitude",
                       "Longitude", "Mort. Status", "Easting", "Northing","t")
head(females_20$t)
```

```
## [1] "2021-02-28 23:00:39 UTC" "2021-02-28 22:00:08 UTC"
## [3] "2021-02-28 21:00:38 UTC" "2021-02-28 20:00:39 UTC"
## [5] "2021-02-28 19:00:08 UTC" "2021-02-28 18:00:09 UTC"
```

```r
# str(females_20)
# table(females_20$CollarID==30140)

# Break dataframe into list object with 1 element per animal
fe_20 <- vector(length = 2, mode = "list")
```

```r
fe_ID <- as.numeric(names(table(females_20$ID)))
for (i in 1:length(fe_20)) {
  sub <- which(females_20$ID ==fe_ID[i])
  fe_20[[i]] <- females_20[sub,]

}
str(fe_20)
```

```
## List of 2
##  $ : tibble [8,160 x 9] (S3: tbl_df/tbl/data.frame)
##   ..$ ID          : num [1:8160] 30174 30174 30174 30174 30174 ...
##   ..$ LMT_Date    : chr [1:8160] "2/28/2021" "2/28/2021" "2/28/2021" "2/28/2021" ...
##   ..$ LMT_Time    : 'hms' num [1:8160] 23:00:39 22:00:08 21:00:38 20:00:39 ...
##   .. ..- attr(*, "units")= chr "secs"
##   ..$ Latitude    : num [1:8160] 39.5 39.5 39.5 39.5 39.5 ...
##   ..$ Longitude   : num [1:8160] -100 -100 -100 -100 -100 ...
##   ..$ Mort. Status: chr [1:8160] "normal" "normal" "normal" "normal" ...
##   ..$ Easting     : num [1:8160] 14405256 14405257 14405301 14405303 14405278 ...
##   ..$ Northing    : num [1:8160] 4377548 4377549 4377577 4377576 4377732 ...
##   ..$ t           : POSIXct[1:8160], format: "2021-02-28 23:00:39" "2021-02-28 22:00:08" ...
##  $ : tibble [6,241 x 9] (S3: tbl_df/tbl/data.frame)
##   ..$ ID          : num [1:6241] 30175 30175 30175 30175 30175 ...
##   ..$ LMT_Date    : chr [1:6241] "12/6/2020" "12/6/2020" "12/6/2020" "12/6/2020" ...
##   ..$ LMT_Time    : 'hms' num [1:6241] 23:00:11 22:00:39 21:00:14 20:00:13 ...
##   .. ..- attr(*, "units")= chr "secs"
##   ..$ Latitude    : num [1:6241] 39.6 39.6 39.6 39.6 39.6 ...
##   ..$ Longitude   : num [1:6241] -100 -100 -100 -100 -100 ...
##   ..$ Mort. Status: chr [1:6241] "normal" "normal" "normal" "normal" ...
##   ..$ Easting     : num [1:6241] 14406335 14406337 14406352 14406341 14406338 ...
##   ..$ Northing    : num [1:6241] 4379220 4379222 4379223 4379218 4379221 ...
##   ..$ t           : POSIXct[1:6241], format: "2020-12-06 23:00:11" "2020-12-06 22:00:39" ...
```

```r
# Define numeric time domain
time.domain <- seq(as.POSIXct("2020-2-1 00:00:00",
                              format="%Y-%m-%d %H:%M:%OS",
                              tz ="UTC"),
                   as.POSIXct("2021-4-27 21:00:00",
                              format="%Y-%m-%d %H:%M:%OS",
                              tz="UTC"), by=1)
# Subset hourly time grid for each deer on numerical time scale
for (i in 1:length(fe_20)) {
  raw_l <- length(fe_20[[i]]$t)
  fe_20[[i]] <- fe_20[[i]][order(fe_20[[i]]$t),]
  dups <- which(duplicated(fe_20[[i]]$t))
  if(length(dups) == 0){
    # Which function indicates which seconds on the time domain have
    # a recorded location. We use this index as our variable for
    # numeric time.
    fe_20[[i]]$num_time <- which(time.domain %in% fe_20[[i]]$t)
    fe_20[[i]]$num_time  <- fe_20[[i]]$num_time / 3600
  }
  else {
    fe_20[[i]] <- fe_20[[i]][-dups,]
    print(paste(i, length(fe_20[[i]]$t) - raw_l))
```

```
    # Which function indicates which seconds on the time domain have
    # a recorded location. We use this index as our variable for
    # numeric time.
    fe_20[[i]]$num_time <- which(time.domain %in% fe_20[[i]]$t)
    fe_20[[i]]$num_time  <- fe_20[[i]]$num_time / 3600
  }
}
```

```
## [1] "1 -1"
```

```
md_traj_list_knn <- knn_bag_move(list_move  = fe_20)
```

```
## Time difference of 27.52014 secs
## Time difference of 19.33764 secs
```

```
f.bar.1lat <- colMeans((md_traj_list_knn[[1]][[6]]))
f.CI.1lat <- t(apply(md_traj_list_knn[[1]][[6]],
                     2, FUN = quantile, prob = c(0.025, 0.975)))
gg_md_1_lat <- gg_temp +
  geom_point(aes(x = num_time,
                 y = Latitude),
             data = fe_20[[1]]) +
  geom_line(aes(x=md_traj_list_knn[[1]][[4]] ,
                y =f.bar.1lat),
            color= "firebrick4") +
  geom_ribbon(aes(x=md_traj_list_knn[[1]][[4]] ,
                  ymin=f.CI.1lat[,1],
                  ymax=f.CI.1lat[,2]), alpha=0.3, fill= "firebrick4") +
  ylab("Latitude")  + xlab(" ")

f.bar.1lon <- colMeans((md_traj_list_knn[[1]][[7]]))
f.CI.1lon <- t(apply(md_traj_list_knn[[1]][[7]],
                     2, FUN = quantile, prob = c(0.025, 0.975)))
gg_md_1_lon <- gg_temp +
  geom_point(aes(x = num_time,
                 y = Longitude),
             data = fe_20[[1]]) +
  geom_line(aes(x=md_traj_list_knn[[1]][[4]] ,
                y =f.bar.1lon),
            color= "firebrick4") +
  geom_ribbon(aes(x=md_traj_list_knn[[1]][[4]] ,
                  ymin=f.CI.1lon[,1],
                  ymax=f.CI.1lon[,2]), alpha=0.3, fill= "firebrick4") +
  ylab("Longitude")  + xlab(" ")

# grid.arrange(gg_md_1_lat, gg_md_1_lon)
```
