# Supplementary material for: Simple Bagged Movement Models for Telemetry Data
Source: Ecol Evol. 2025 Sep 7;15(9):e72060. doi: 10.1002/ece3.72060 (PMC12414728; doi:10.1002/ece3.72060)
Supplement: Supplementary file 1 — Appendices S1–S13: ece372060‐sup‐0001‐AppendicesS1‐S13.zip. [file ECE3-15-e72060-s001.zip › ece372060-sup-0002-AppendixS2.pdf]

Appendix 2

Andrew B. Whetten, Ph.D.

Contents

Section 1: Introduction 1
Section 2: The importance of assessing frequentist properties of animal movement models 1
Section 3: Stepwise movement simulation study 2
    Generating true stepwise movement path 2
    K-nearest neighbor bagging movement model function 4
    Code for stepwise movement simulation study 8
    Code for visualization of results 13
Section 4: Drifting Circling Simulation Study 17
    Generating true drifting circles movement path 17
    Weighted K-nearest neighbor bagging movement model function 20
    Code for drifting circle simulation study 23
    Code for visualization of results 27
    Code for Visualization of Results 27
Section 5: Disclaimer about adapting bagging functions for applied use 31

Section 1: Introduction

This appendix provides documentation and R code pertaining to the simulation study. The code is intended to be compiled in the order that it is presented. In Section 2, we briefly provide motivation for the simulation study. In Sections 3-4, we provide code needed to reproduce simulation study results. In Section 5, we provide more details about adapting the example bagged k-nearest neighbor functions presented in this appendix for applied use.

Section 2: The importance of assessing frequentist properties of animal movement models

In our simulation study, we consider two movement patterns: abrupt step-wise movement and drifting circles movement. These movement patterns can be generated from continuous functions that represent the true movement path of a hypothetical animal. In Sections 3 and 4, we illustrate how these true movement paths can be generated. For any true movement path, there are important descriptors of movement that also have true values. As an example, for a true movement path, there is a true daily displacement describing the difference between the starting and ending location of an animal each day. In the applied setting, these descriptors of the true movement path (e.g., daily displacement) are quantities that we seek to estimate from telemetry data. These estimates can be obtained as model-based summary statistics (i.e., derived quantities) from an animal movement model.

It is important to assess the properties of estimates arising from animal movement models for quantities that have a true value. Bias (i.e., the difference between the expected value of our estimates and the true value) and coverage probability (i.e., the proportion of time confidence interval estimates covers the true value) are frequentist properties that improve our understanding of the quality of estimates from our model. Understanding bias and coverage probabilities of estimates from our model in realistic simulation studies can support appropriate model use.

Section 3: Stepwise movement simulation study

The code in this section allows readers to reproduce results for the stepwise movement simulation study. Further description of the simulation study can be found in the manuscript.

Generating true stepwise movement path

```
######################################################################
######  Stepwise movement ############################################
######################################################################
y_lat <- c(rep(1000, 10600), seq(1000, 5000, by = 10), rep(5000,10599))
y_lon <- c(rep(1000, 10600), seq(1000, 5000, by = 10), rep(5000,10599))

e_tlat <- rnorm(21600, mean = 0, sd = 12)
e_tlon <- rnorm(21600, mean = 0, sd = 12)
y_lat <- y_lat + e_tlat
y_lon <- y_lon + e_tlon

road_dat_y <- c(seq(from = 1000, to = 4000, length.out = 3000),
                rep(3500, length.out = 3000),
                seq(from=1000, to = 3500, length.out = 2500),
                rep(4000, length.out = 1000))
road_dat_x <- c(rep(2000, length.out = 3000),
                seq(from = 2000, to = 5000, length.out = 3000),
                rep(4000, length.out = 2500),
                seq(from=1000, to=2000, length.out = 1000))

road_dat <- cbind(road_dat_x, road_dat_y)

plot(y_lon, y_lat)
points(road_dat[,1], road_dat[,2])
```

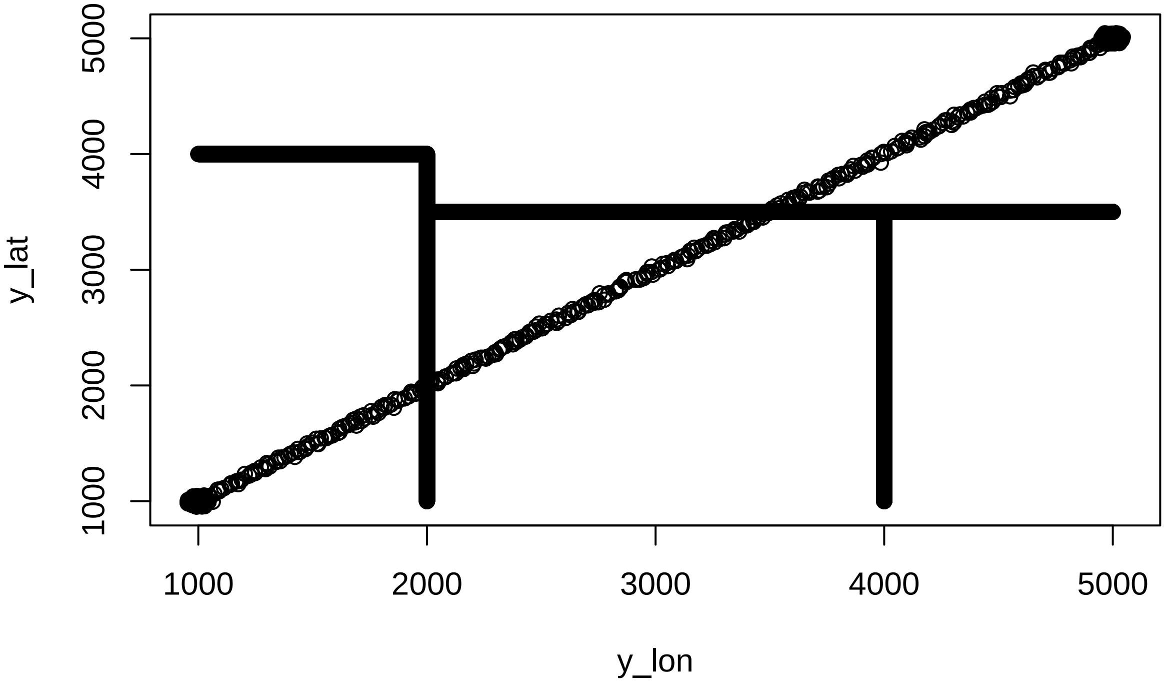

```r
##############################################################
#### True Daily Disp Traveled ##########################
##############################################################
dat_mat <- cbind(y_lon, y_lat)

daily_disp_subset <- seq(1,21600, by=24*60)
disp_locations <- dat_mat[daily_disp_subset,]

dist_mat <- as.matrix(dist(disp_locations))
#plot(dist_mat[1,])

true_dist_vt <- as.numeric()
for (i in 1:(length(disp_locations[,1])-1)) {
  true_dist_vt <- c(true_dist_vt, dist_mat[i, i+1])
}
plot(true_dist_vt)
```

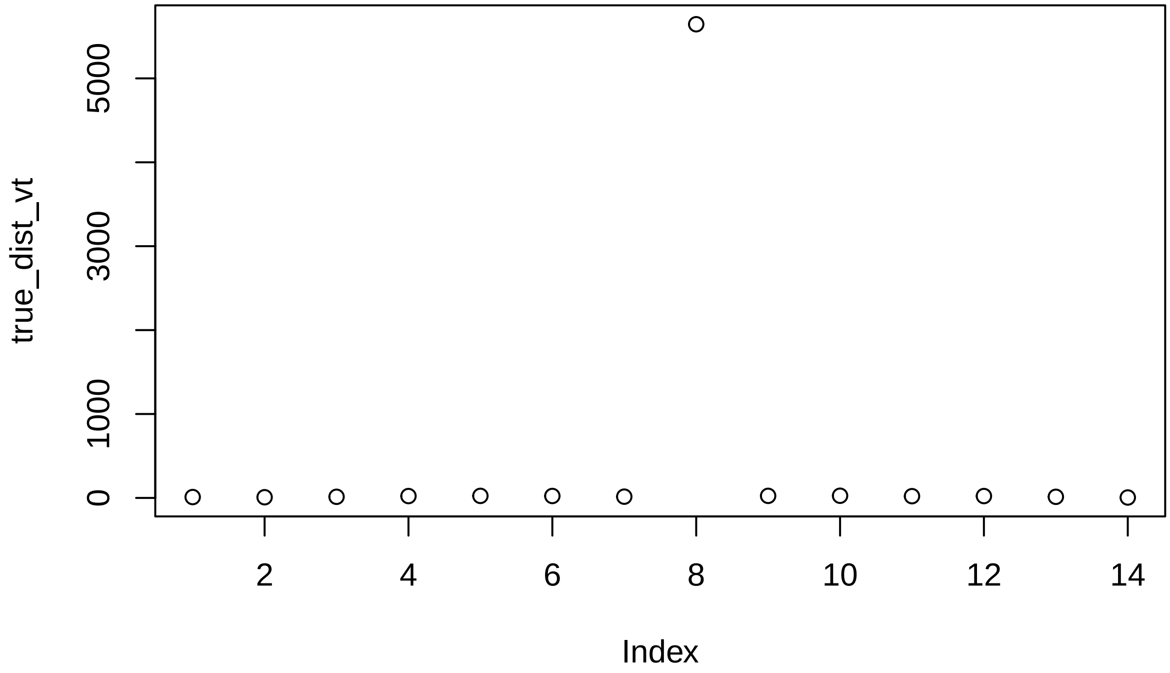


```r
##################################################################
#### True Proportion of Time Spent within 200m of roadway ######
##################################################################
library(FNN)
knn_prop_true <-knnx.dist(data = road_dat, query = dat_mat, k=1)
str(knn_prop_true)
```

```
##  num [1:21600, 1] 989 1004 1005 991 1001 ...
```

```r
plot(knn_prop_true)
lines(1:22000, rep(200, times=22000), col= "red")
```

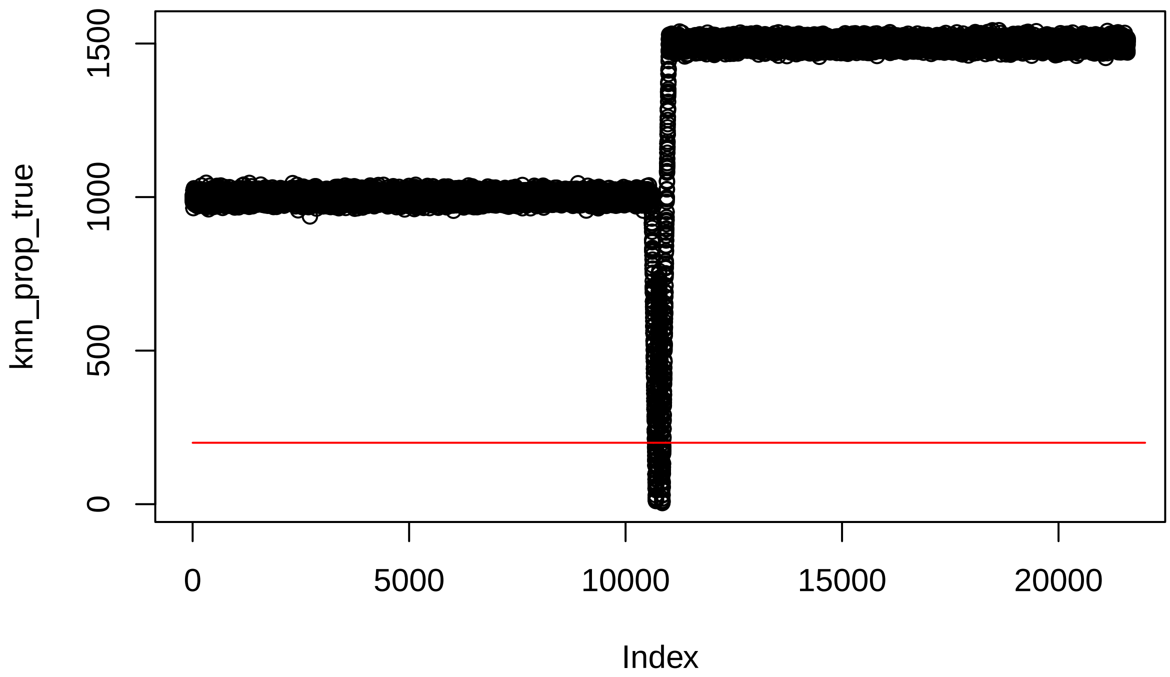


```
table(knn_prop_true <200)
```

```
##
## FALSE  TRUE
## 21518    82
```

```
true_prop_200 <-table(knn_prop_true <200)[2]/(table(knn_prop_true <200)[1] +
                                                 table(knn_prop_true <200)[2])
```

K-nearest neighbor bagging movement model function

```
library(EnvStats)
library(FNN)

# tel_dat = telemetry_dat
#  K=neighbors
# num_subsamples = num_subsamps

knn_bag_move <- function(tel_dat, bag_samps = 500, pred_time_incr =1.0,
                         K = 4, num_subsamples = 60){

  list_in_bag <- vector(length = length(bag_samps), mode = "list")
  list_oob <- vector(length = length(bag_samps), mode = "list")

  num_time <- seq(floor(min(tel_dat$num_time)),
                  ceiling(max(tel_dat$num_time)),
                  by = pred_time_incr)
  new_dat <- as.data.frame(cbind(num_time))
  mat_pred_lon <- matrix(data=NA, nrow = bag_samps, ncol=length(num_time))
  mat_pred_lat <- matrix(data=NA, nrow = bag_samps, ncol=length(num_time))
  mat_pred_train_lon <- matrix(data=NA, nrow = bag_samps, ncol=length(tel_dat$num_time))
  mat_pred_train_lat <- matrix(data=NA, nrow = bag_samps, ncol=length(tel_dat$num_time))
  mat_oob_lon <- matrix(data=NA, nrow = bag_samps, ncol=length(tel_dat$num_time))
```

```
mat_oob_lat <- matrix(data=NA, nrow = bag_samps, ncol=length(tel_dat$num_time))
mat_oob_res_lon <- matrix(data=NA, nrow = bag_samps, ncol=length(tel_dat$num_time))
mat_oob_res_lat <- matrix(data=NA, nrow = bag_samps, ncol=length(tel_dat$num_time))
var_vt_lon <- as.numeric()
var_vt_lat <- as.numeric()

temp_mat_boot <- matrix(nrow = length(tel_dat$num_time),
                        ncol = bag_samps)

list_store <- vector(length = 7, mode = "list")

for (i in 1:bag_samps) {

  time_temp <- tel_dat$num_time
  train_dat <- as.data.frame(time_temp)
  names(train_dat) <- "num_time"

  # get the in-bag and oob samples
  subsamp_temp <- sort(rep(1:num_subsamples,
                           length.out = length(tel_dat$num_time)))
  samp_inbag <- sample(1:num_subsamples, size = num_subsamples,
                       replace=TRUE)
  samp_oob <- subsamp_temp[subsamp_temp %in% samp_inbag ==FALSE]
  df_subsamp_temp <- as.data.frame(cbind(1:length(tel_dat$num_time),
                                         subsamp_temp))

  which(df_subsamp_temp$subsamp_temp %in% samp_inbag)

  index_vt_final <- as.numeric()
  for (j in 1:length(df_subsamp_temp$subsamp_temp)) {
    # j=7
    meep <- df_subsamp_temp$subsamp_temp[j]
    if(df_subsamp_temp$subsamp_temp[j] %in% samp_inbag == TRUE){
      num_occur_temp <-sum(samp_inbag == meep)
      index_vt_final <- c(index_vt_final, rep(j,
                                              times = num_occur_temp))
    }
  }
  table(index_vt_final)

  # in-bag data as df for bagged model

  # df_in_bag_temp <- tel_dat[index_vt_final,]
  # # df_ex_temp <- df_ex[samp_inbag,]
  # #oob data as df
  # oob_dat <- as.data.frame(tel_dat[df_subsamp_temp$V1[subsamp_temp %in%
  #                                                      samp_oob],3])
  # # oob_dat <- as.data.frame(samp_oob)
  # names(oob_dat) <- "num_time"

  # in-bag data as df for bagged model
    list_in_bag[[i]] <- tel_dat[index_vt_final,]
```

```r
#oob data as df
  list_oob[[i]] <- as.data.frame(tel_dat$num_time[-index_vt_final])

  names(list_oob[[i]]) <- "num_time"



# in-bag model
knn_temp_oob_lon <- knn.reg(train=list_in_bag[[i]]$num_time,
                            test= list_oob[[i]],
                            y = list_in_bag[[i]]$y_lon,
                            k=K)
knn_temp_oob_lat <- knn.reg(train=list_in_bag[[i]]$num_time,
                            test= list_oob[[i]],
                            y = list_in_bag[[i]]$y_lat,
                            k=K)
knn_temp_train_lon <- knn.reg(train=list_in_bag[[i]]$num_time,
                              test= train_dat,
                              y = list_in_bag[[i]]$y_lon,
                              k=K)
knn_temp_train_lat <- knn.reg(train=list_in_bag[[i]]$num_time,
                              test= train_dat,
                              y = list_in_bag[[i]]$y_lat,
                              k=K)
knn_temp_pred_lon <- knn.reg(train=list_in_bag[[i]]$num_time,
                             test= new_dat,
                             y = list_in_bag[[i]]$y_lon,
                             k=K)
knn_temp_pred_lat <- knn.reg(train=list_in_bag[[i]]$num_time,
                             test= new_dat,
                             y = list_in_bag[[i]]$y_lat,
                             k=K)

# get residuals for oob data
oob_pred_temp_lon <- knn_temp_oob_lon$pred
oob_pred_temp_lat <- knn_temp_oob_lat$pred
mat_oob_lon[i, samp_oob] <- oob_pred_temp_lon
mat_oob_lat[i, samp_oob] <- oob_pred_temp_lat

# Store record of in-bag times used
temp_mat_boot[,i] <- list_in_bag[[i]]$num_time

# df_subsamp_temp$V1[subsamp_temp %in% samp_inbag]
res_temp_lon <- tel_dat$y_lon[df_subsamp_temp$V1[subsamp_temp %in%
                                                   samp_oob]]
- oob_pred_temp_lon
res_temp_lat <- tel_dat$y_lat[df_subsamp_temp$V1[subsamp_temp %in%
                                                   samp_oob]]
- oob_pred_temp_lat

mat_oob_res_lon[i, samp_oob] <- res_temp_lon
mat_oob_res_lat[i, samp_oob] <- res_temp_lat
var_vt_lon <- c(var_vt_lon, var(res_temp_lon))
```

```
  var_vt_lat <- c(var_vt_lat, var(res_temp_lat))
  # predictions for time grid/test data times
  mat_pred_train_lon[i,] <- knn_temp_train_lon$pred
  mat_pred_train_lat[i,] <- knn_temp_train_lat$pred
  mat_pred_lon[i,]  <- knn_temp_pred_lon$pred#
  mat_pred_lat[i,]  <- knn_temp_pred_lat$pred#
  #
  # samp_temp <- sample(1:10, size =10, replace=TRUE)
  # knn_temp <- knn.reg(train=df_ex[samp_temp,1],
  #                     test= new_dat,
  #                     y = df_ex$y1[samp_temp],
  #                     k=2)
  # mat_pred[i,]  <- knn_temp$pred
}


pred_int_net_lat <- apply(mat_oob_res_lat,1,sd, na.rm = TRUE)
  pred_int_net_lon <- apply(mat_oob_res_lon,1,sd, na.rm = TRUE)

# mat_oob_res_vt_lat <-as.vector(mat_oob_res_lat)
# mat_oob_res_vt_lon <-as.vector(mat_oob_res_lon)
#
# sd_knn_res_lat <- sd(mat_oob_res_vt_lat, na.rm = TRUE)
# sd_knn_res_lon <- sd(mat_oob_res_vt_lon, na.rm = TRUE)

mat_paths_lat <- matrix(data=NA, ncol= length(num_time), nrow=bag_samps)
mat_paths_lon <- matrix(data=NA, ncol= length(num_time), nrow=bag_samps)

for (i in 1:bag_samps) {
  # n=3
  # use expected value of the animal's path and the information from the
  # prediction interval for each location to generate potential paths
  # of the animal using knn regression
  time_temp <- tel_dat$num_time

  location_temp_lat <- rnorm(n = length(tel_dat$y_lat),
                              mean = list_in_bag[[i]]$y_lat, sd = pred_int_net_lat)
  location_temp_lon <- rnorm(n = length(tel_dat$y_lat),
                               mean = list_in_bag[[i]]$y_lon, sd = pred_int_net_lon)
  # location_temp_lat <- rnorm(n = length(tel_dat$y_lat),
  #                            mean = tel_dat$y_lat, sd = sd_knn_res_lat)
  # location_temp_lon <- rnorm(n = length(tel_dat$y_lon),
  #                            mean = tel_dat$y_lon, sd = sd_knn_res_lon)
  knn_temp_lat <- knn.reg(train=time_temp,
                          test= as.data.frame(num_time),
                          y = location_temp_lat,
                          k=K)
  knn_temp_lon <- knn.reg(train=time_temp,
                          test= as.data.frame(num_time),
                          y = location_temp_lon ,
                          k=K)
  mat_paths_lat[i,] <- knn_temp_lat$pred
  mat_paths_lon[i,] <- knn_temp_lon$pred
```

```
  }

  list_store[[1]] <- temp_mat_boot
  list_store[[2]] <- mat_pred_lat
  list_store[[3]] <- mat_pred_lon
  list_store[[4]] <- num_time
  list_store[[5]] <- "fill with Animal ID"
  list_store[[6]] <- mat_paths_lat
  list_store[[7]] <- mat_paths_lon

  return(list_store)
}
```

Code for stepwise movement simulation study

```
#################################################################
#################################################################
#### For each scenario: generate dataset 500 times
#### and estimate derived quantities
#################################################################
#################################################################

library(spatstat.utils)
# library(foreach)
# library(doParallel)

# data = dat_mat
# samp_size = 300
# num_sims = 250
# rad = 200
# loc_err = "high"
# num_subsamps=60
# neighbors = 4

bagged_simulation <- function(data, samp_size = 300, num_sims = 500,
                              rad = 100, loc_err = "low",
                              num_subsamps=60, neighbors = 4){


  list_temp <- vector(mode = "list", length = 6)
  list_sim_sum <- vector(mode = "list", length = 6)

  # extract data dimensions
  dat_len <- dim(data)[1]
  tel_time <- 1:dat_len

  # Storage
  # Matrices for estimated animal location (lat/lon)
  mat_lon <- matrix(data = NA, nrow = num_sims, ncol = dat_len)
  mat_lat <- matrix(data = NA, nrow = num_sims, ncol = dat_len)
  # Matrice for estimated daily displacement
  mat_daily_disp <- matrix(data = NA, nrow = num_sims, ncol = dat_len/(24*60)-2)
  # Vector for CP  of daily displacement on disperal day
```

```
disp_covered <- as.character()
# Vector for proportion of time spent within r dist of object
prop_time_r <- as.numeric()
# Vector for CP of prop time
prop_covered <- as.character()

# numcores <- 5
# registerDoParallel(numcores)
startTime <- Sys.time()
# #c(2,4,6)
# # 2:41
# r <- foreach (j = 1:num_sims) %dopar% {
for (j in 1:num_sims) {


  # j=1
  tel_time_temp <- sort(c(tel_time[1],
                          sample(tel_time[2]:tel_time[dat_len-1],
                                 size = samp_size-2,
                                 prob = c(rep(1/21600, 10599),
                                          rep(2/21600, 401),
                                          rep(1/21600, 10598))), tel_time[dat_len]))
  telemetry_dat <- as.data.frame(dat_mat[tel_time_temp,])
  telemetry_dat$num_time <- tel_time_temp
  if(loc_err == "low"){
    # Add device location error with randomly selected distribution
    for (i in 1:length(tel_time_temp)) {
      # for (i in 1:300) {
      dist_choice <- sample(c("Normal", "Triangular", "Uniform"),
                            size =1)
      if(dist_choice == "Normal"){
        e_dlat <- rnorm(1, mean = 0, sd = 10)
        e_dlon <- rnorm(1, mean = 0, sd = 10)
        telemetry_dat$y_lon[i] <- telemetry_dat$y_lon[i] + e_dlat
        telemetry_dat$y_lat[i] <- telemetry_dat$y_lat[i] + e_dlon
      }
      if(dist_choice == "Triangular"){
        e_dlat <- rtri(1, min = -12, max=12, mode = 0)
        e_dlon <- rtri(1, min = -12, max=12, mode = 0)
        telemetry_dat$y_lon[i] <- telemetry_dat$y_lon[i] + e_dlat
        telemetry_dat$y_lat[i] <- telemetry_dat$y_lat[i] + e_dlon
        }
      if(dist_choice == "Uniform"){
        e_dlat <- runif(1, min = -15, max = 15)
        e_dlon <- runif(1, min = -15, max = 15)
        telemetry_dat$y_lon[i] <- telemetry_dat$y_lon[i] + e_dlat
        telemetry_dat$y_lat[i] <- telemetry_dat$y_lat[i] + e_dlon
      }
    }

  }
  if(loc_err == "high"){
    for (i in 1:length(tel_time_temp)) {
```

```r
    # for (i in 1:300) {
    dist_choice <- sample(c("Normal", "Triangular", "Uniform"), size =1)
    if(dist_choice == "Normal"){
      e_dlat <- rnorm(1, mean = 0, sd = 100)
      e_dlon <- rnorm(1, mean = 0, sd = 100)
      telemetry_dat$y_lon[i] <- telemetry_dat$y_lon[i] + e_dlat
      telemetry_dat$y_lat[i] <- telemetry_dat$y_lat[i] + e_dlon
    }
    if(dist_choice == "Triangular"){
      e_dlat <- rtri(1, min = -120, max=120, mode = 0)
      e_dlon <- rtri(1, min = -120, max=120, mode = 0)
      telemetry_dat$y_lon[i] <- telemetry_dat$y_lon[i] + e_dlat
      telemetry_dat$y_lat[i] <- telemetry_dat$y_lat[i] + e_dlon
    }
    if(dist_choice == "Uniform"){
      e_dlat <- runif(1, min = -150, max = 150)
      e_dlon <- runif(1, min = -150, max = 150)
      telemetry_dat$y_lon[i] <- telemetry_dat$y_lon[i] + e_dlat
      telemetry_dat$y_lat[i] <- telemetry_dat$y_lat[i] + e_dlon
    }
  }
}

move_mod_temp <- knn_bag_move(tel_dat = telemetry_dat,
                                   K=neighbors,
                              num_subsamples = num_subsamps)
# Location estimation
f.bar.1lat <- colMeans(move_mod_temp[[6]], na.rm = TRUE)
f.bar.1lon <- colMeans((move_mod_temp[[7]]), na.rm=TRUE)
f.CI.1lat <- t(apply(move_mod_temp[[6]],
                     2, FUN = quantile, prob = c(0.025, 0.975), na.rm=TRUE))
# Displacement Estimation
daily_disp_subset <- seq(1,dat_len, by=24*60)
disp_mat_dist <- matrix(nrow=500, ncol=length(daily_disp_subset)-2)

for (u in 1:500) {
  # u=1
  disp_locations <- cbind(move_mod_temp[[7]][u, daily_disp_subset],
                          move_mod_temp[[6]][u,daily_disp_subset])
  disp_mat <- as.matrix(dist(disp_locations))
  dist_vt <- as.numeric()

  for (v in 1:(length(disp_locations[,1])-2)) {
    dist_vt <- c(dist_vt, disp_mat[v, v+1])
  }
  disp_mat_dist[u,] <- dist_vt

}

f.bar.disp <- colMeans(disp_mat_dist)
f.CI.disp <- t(apply(disp_mat_dist,
                   2, FUN = quantile, prob = c(0.025, 0.975), na.rm=TRUE))
```

```r
disp_covered_temp <- check.in.range(true_dist_vt[8],
                                   f.CI.disp[8,],
                                   fatal = FALSE)

# Estimation of Proportion of time within r dist
knn_mat <- matrix(nrow = dim(move_mod_temp[[2]])[2],
                  ncol = dim(move_mod_temp[[2]])[1])
prop_wthn_200m <- as.numeric()

for (i in 1:dim(move_mod_temp[[7]])[1]) {
  # i=1
  mat_temp <- cbind(move_mod_temp[[7]][i,],
                    move_mod_temp[[6]][i,])
  # head(mat_temp)
  knn_temp <-knnx.dist(data = road_dat, query = mat_temp, k=1)

  prop_wthn_200m <- c(prop_wthn_200m,
                      as.numeric(table(knn_temp < rad)[2]/
                                   (table(knn_temp < rad)[2]+
                                      table(knn_temp < rad)[1])))
  knn_mat[,i] <- knn_temp
}

# hist(prop_wthn_200m, breaks=15)
# abline(v=table(knn_prop_true <200)[2]/(table(knn_prop_true <200)[1] +
#                                        table(knn_prop_true <200)[2]))
prop_time_temp <- mean(prop_wthn_200m, na.rm = TRUE)
cp_temp <-quantile(prop_wthn_200m, probs = c(0.025, 0.975), na.rm = TRUE)

prop_covered_temp <- check.in.range(true_prop_200,cp_temp,
                                   fatal = FALSE)


# Store Important Information for Simulation Summary
# CIs for Location
mat_lon[j,] <- f.bar.1lon
mat_lat[j,] <- f.bar.1lat
# CIs/Point estimates of daily displacement
mat_daily_disp[j,] <- f.bar.disp
# CP information for Daily Displacement on Extreme Movement Day
disp_covered <- c(disp_covered, disp_covered_temp)
# CIs/Point Estimates of Prop Time
prop_time_r <- c(prop_time_r, prop_time_temp)
# CP information for Prop Time
prop_covered <- c(prop_covered, prop_covered_temp)

# list_temp[[1]] <- mat_lat
# list_temp[[2]]  <- mat_lon
# list_temp[[3]]  <- mat_daily_disp
# list_temp[[4]]  <- disp_covered
# list_temp[[5]]  <- prop_time_r
list_temp[[6]]  <- prop_covered
if(j%%25 == 0){
```

```r
      endTime <- Sys.time()
      t_check <- endTime - startTime
      time_update <- paste0("Sim", " ", j, ": Elasped Time =", t_check)
      print(time_update)
      }

    else{print(j)}
  }


  # Store in cool list object
  list_sim_sum[[1]] <- mat_lat
  list_sim_sum[[2]]  <- mat_lon
  list_sim_sum[[3]]  <- mat_daily_disp
  list_sim_sum[[4]]  <- disp_covered
  list_sim_sum[[5]]  <- prop_time_r
  list_sim_sum[[6]]  <- prop_covered

  return(list_sim_sum)
}
```

```r
# Run Simulation function for various scenarios
set.seed(328)
set.seed(45)
sim_higherr_300 <- bagged_simulation(data = dat_mat, samp_size = 300,
                                     num_sims = 250, rad = 200,
                                     loc_err = "high",
                                     num_subsamps=60, neighbors = 4)

set.seed(4)
sim_higherr_100 <- bagged_simulation(data = dat_mat, samp_size = 100,
                                     num_sims = 500, rad = 200,
                                     loc_err = "high",
                                     num_subsamps=20, neighbors = 4)

set.seed(6509)
sim_lowerr_300 <- bagged_simulation(data = dat_mat, samp_size = 300,
                                    num_sims = 500, rad = 200,
                                    loc_err = "low",
                                    num_subsamps=60, neighbors = 4)

set.seed(201)
sim_lowerr_100 <- bagged_simulation(data = dat_mat, samp_size = 100,
                                     num_sims = 500, rad = 200,
                                     loc_err = "low",
                                     num_subsamps=20, neighbors = 4)

str(sim_higherr_300)

hist(sim_higherr_300[[3]][,8])
table(sim_higherr_300[[4]])
(true_dist_vt[8] - mean(sim_higherr_300[[3]][,8]))/true_dist_vt[8]

hist(sim_higherr_300[[5]])
```

```
(true_prop_200 - mean(sim_higherr_300[[5]]))/true_prop_200
table(sim_higherr_300[[6]])

hist(sim_higherr_100[[3]][,8])
table(sim_higherr_100[[4]])
(true_dist_vt[8] - mean(sim_higherr_100[[3]][,8]))/true_dist_vt[8]

hist(sim_higherr_100[[5]])
(true_prop_200 - mean(sim_higherr_100[[5]]))/true_prop_200
table(sim_higherr_100[[6]])



hist(sim_lowerr_300[[3]][,8])
table(sim_lowerr_300[[4]])
(true_dist_vt[8] - mean(sim_lowerr_300[[3]][,8]))/true_dist_vt[8]

hist(sim_lowerr_300[[5]])
(true_prop_200 - mean(sim_lowerr_300[[5]]))/true_prop_200
table(sim_lowerr_300[[6]])

hist(sim_lowerr_100[[3]][,8])
table(sim_lowerr_100[[4]])
(true_dist_vt[8] - mean(sim_lowerr_100[[3]][,8]))/true_dist_vt[8]

hist(sim_lowerr_100[[5]])
(true_prop_200 - mean(sim_lowerr_100[[5]]))/true_prop_200
table(sim_lowerr_100[[6]])
```

Code for visualization of results

```
# Simulation Summary Figure 3 Panels
str(sim_higherr_300)
library(ggplot2)
library(RColorBrewer)
library(gridExtra)
gg_temp <- ggplot() +
  theme(text = element_text(family="Times",size=20),
        plot.title = element_text(size = 20),
        axis.text.x=element_text(size=20),
        axis.text.y=element_text(size=20),
        panel.background = element_rect(fill = "white", colour = "black"),
        panel.grid.major = element_blank(),
        panel.grid.major.y=element_blank(),
        legend.position = "none")

# extract data dimensions
dat_len <- dim(dat_mat)[1]
tel_time <- 1:dat_len
tel_time_temp <- sort(c(tel_time[1],
                        sample(tel_time[2]:tel_time[dat_len-1],
                               size = 100-2,
                               prob = c(rep(1/21600, 10599),
```

```
                                        rep(2/21600, 401),
                                        rep(1/21600, 10598))), tel_time[dat_len]))
telemetry_dat <- as.data.frame(dat_mat[tel_time_temp,])
telemetry_dat$num_time <- tel_time_temp
  for (i in 1:length(tel_time_temp)) {
    # for (i in 1:300) {
    dist_choice <- sample(c("Normal", "Triangular", "Uniform"), size =1)
    if(dist_choice == "Normal"){
      e_dlat <- rnorm(1, mean = 0, sd = 100)
      e_dlon <- rnorm(1, mean = 0, sd = 100)
      telemetry_dat$y_lon[i] <- telemetry_dat$y_lon[i] + e_dlat
      telemetry_dat$y_lat[i] <- telemetry_dat$y_lat[i] + e_dlon
    }
    if(dist_choice == "Triangular"){
      e_dlat <- rtri(1, min = -120, max=120, mode = 0)
      e_dlon <- rtri(1, min = -120, max=120, mode = 0)
      telemetry_dat$y_lon[i] <- telemetry_dat$y_lon[i] + e_dlat
      telemetry_dat$y_lat[i] <- telemetry_dat$y_lat[i] + e_dlon
    }
    if(dist_choice == "Uniform"){
      e_dlat <- runif(1, min = -150, max = 150)
      e_dlon <- runif(1, min = -150, max = 150)
      telemetry_dat$y_lon[i] <- telemetry_dat$y_lon[i] + e_dlat
      telemetry_dat$y_lat[i] <- telemetry_dat$y_lat[i] + e_dlon
    }
  }



gg_map <- gg_temp +
  geom_point(data=telemetry_dat, aes(x=y_lon, y=y_lat),
             alpha =0.4, color = "darkorange", size = 3.5)+
  geom_line(data = as.data.frame(road_dat),
             aes(x=road_dat_x, y=road_dat_y), col = "lemonchiffon4", size = 4.0) +
  geom_line(data = as.data.frame(dat_mat), aes(x=y_lon, y=y_lat), color = "mediumblue", size=1.2) +
  ylab("Y (m)") + xlab("X (m)")


gg_disp8 <- gg_temp +
  geom_violin(aes(x= "High Loc Err:\n n=300", y=sim_higherr_300[[3]][,8]),
              fill = "turquoise", alpha=0.3) +
  geom_boxplot(aes(x= "High Loc Err:\n n=300", y=sim_higherr_300[[3]][,8]),
               width =0.4) +
  geom_violin(aes(x= "High Loc Err:\n n=100", y=sim_higherr_100[[3]][,8]),
              fill = "turquoise", alpha=0.3) +
  geom_boxplot(aes(x= "High Loc Err:\n n=100", y=sim_higherr_100[[3]][,8]),
               width =0.4) +
  geom_violin(aes(x= "Low Loc Err:\n n=300", y=sim_lowerr_300[[3]][,8]),
              fill = "turquoise", alpha=0.3) +
  geom_boxplot(aes(x= "Low Loc Err:\n n=300", y=sim_lowerr_300[[3]][,8]),
               width =0.4) +
  geom_violin(aes(x= "Low Loc Err:\n n=100", y=sim_lowerr_100[[3]][,8]),
              fill = "turquoise", alpha=0.3) +
```

```
  geom_boxplot(aes(x= "Low Loc Err:\n n=100", y=sim_lowerr_100[[3]][,8]),
               width =0.4) +
  geom_hline(yintercept = 5658.5, linetype = "dashed")+
  ylab("Daily Displacement on 8th Day (m)") + xlab(" ")

gg_p200 <-gg_temp +
  geom_violin(aes(x= "High Loc Err:\n n=300", y=sim_higherr_300[[5]]),
              fill = "goldenrod4", alpha=0.3) +
  geom_boxplot(aes(x= "High Loc Err:\n n=300", y=sim_higherr_300[[5]]),
               width =0.4) +
  geom_violin(aes(x= "High Loc Err:\n n=100", y=sim_higherr_100[[5]]),
              fill = "goldenrod4", alpha=0.3) +
  geom_boxplot(aes(x= "High Loc Err:\n n=100", y=sim_higherr_100[[5]]),
               width =0.4) +
  geom_violin(aes(x= "Low Loc Err:\n n=300", y=sim_lowerr_300[[5]]),
              fill = "goldenrod4", alpha=0.3) +
  geom_boxplot(aes(x= "Low Loc Err:\n n=300", y=sim_lowerr_300[[5]]),
               width =0.4) +
  geom_violin(aes(x= "Low Loc Err:\n n=100", y=sim_lowerr_100[[5]]),
              fill = "goldenrod4", alpha=0.3) +
  geom_boxplot(aes(x= "Low Loc Err:\n n=100", y=sim_lowerr_100[[5]]),
               width =0.4) +
  geom_hline(yintercept = 0.0037, linetype = "dashed")+
  ylab("Proportion of Time wthn 200m of Roadway") + xlab("") +ylim(0,0.015)

gg_lower <-grid.arrange(gg_disp8, gg_p200, ncol=2)

#setwd("")
#png("simulation_abrupt_step_f.png", width = 3200, height = 3600, res = 200)
grid.arrange(gg_map, gg_lower, ncol=1)
#dev.off()
```

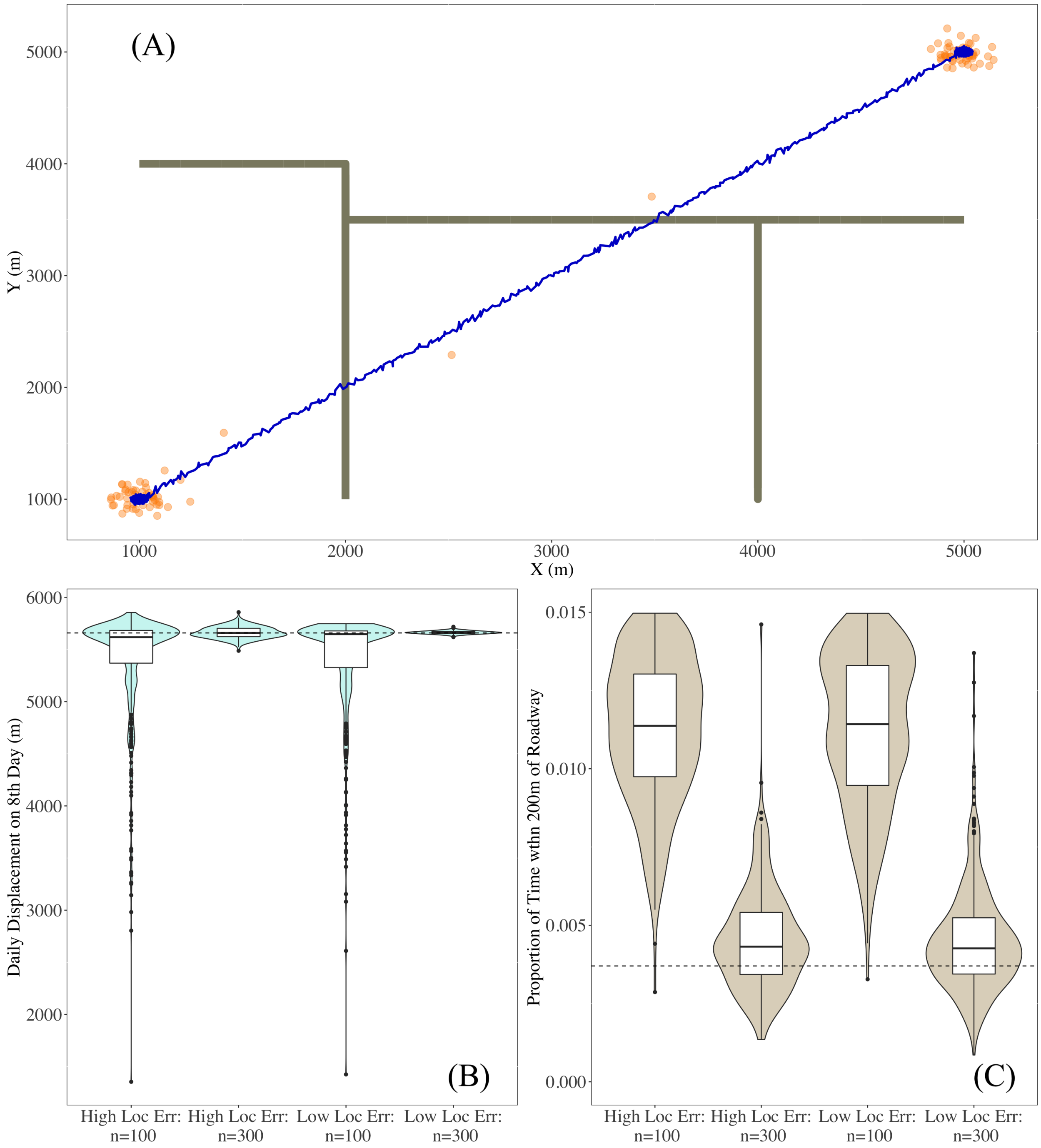
(A)
Y (m)
X (m)
1000
2000
3000
4000
5000
Daily Displacement on 8th Day (m)
(B)
Proportion of Time wthn 200m of Roadway
(C)
0.000
0.005
0.010
0.015
6000
High Loc Err: n=100
High Loc Err: n=300
Low Loc Err: n=100
Low Loc Err: n=300

Section 4: Drifting Circling Simulation Study

The code in this section allows readers to reproduce results for the drifting circles movement simulation study. Further description of the simulation study can be found in the manuscript.

Generating true drifting circles movement path

```
##########################################################################
##########################################################################
### Drifting Circles Simulation
##########################################################################
##########################################################################


#################################################################
##### Drifting Circle movement ##################################

# Wind turbine Locations
windmill_mat <- cbind(c(50,550, 540, 1200), c(350, 1200, 590, 600))

t <- 1:1500
diam_vt <- c(seq(100,300, length.out = 750),seq(300,100, length.out = 750))

y_lat <- diam_vt *sin(0.1*t) + t
y_lon <- diam_vt *cos(0.1*t) + t

e_tlat <- rnorm(1500, mean = 0, sd = 5)
e_tlon <- rnorm(1500, mean = 0, sd = 5)


############################################################
#### True Daily Disp Traveled ##########################
############################################################
dat_mat <- cbind(y_lon, y_lat)

daily_disp_subset <- seq(1,1500, by=60)
disp_locations <- dat_mat[daily_disp_subset,]

dist_mat <- as.matrix(dist(disp_locations))
plot(dist_mat[1,])
```

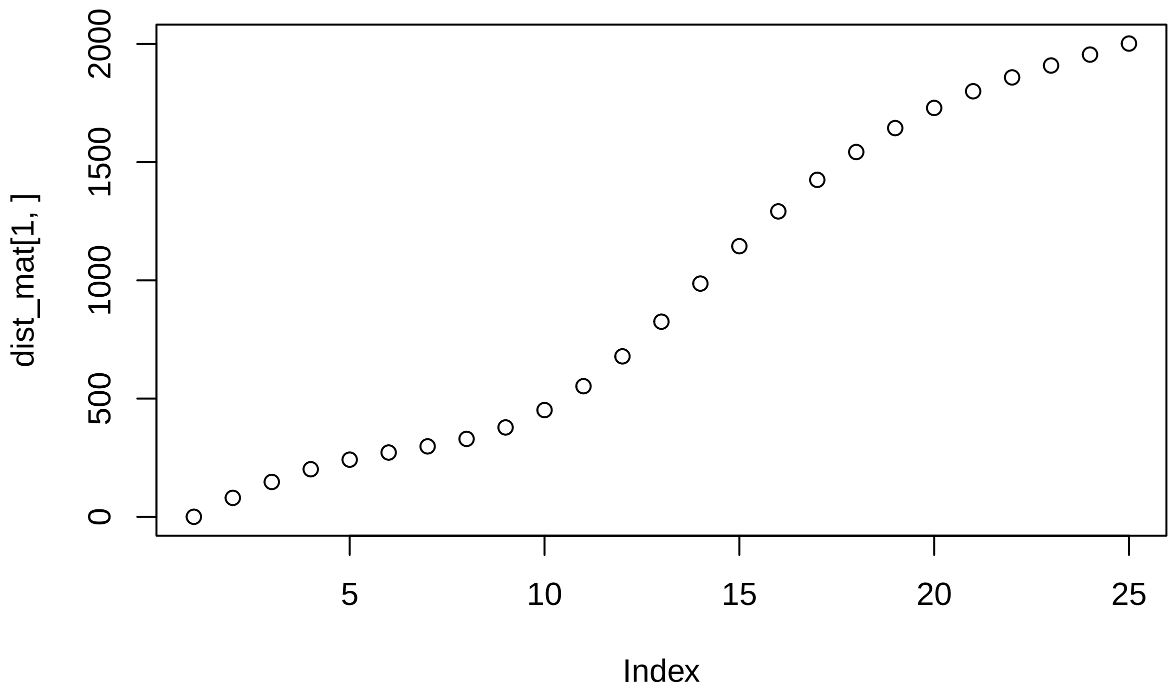


```
true_dist_vt <- as.numeric()
for (i in 1:(length(disp_locations[,1])-2)) {
  true_dist_vt <- c(true_dist_vt, dist_mat[i, i+1])
}
plot(true_dist_vt)
```

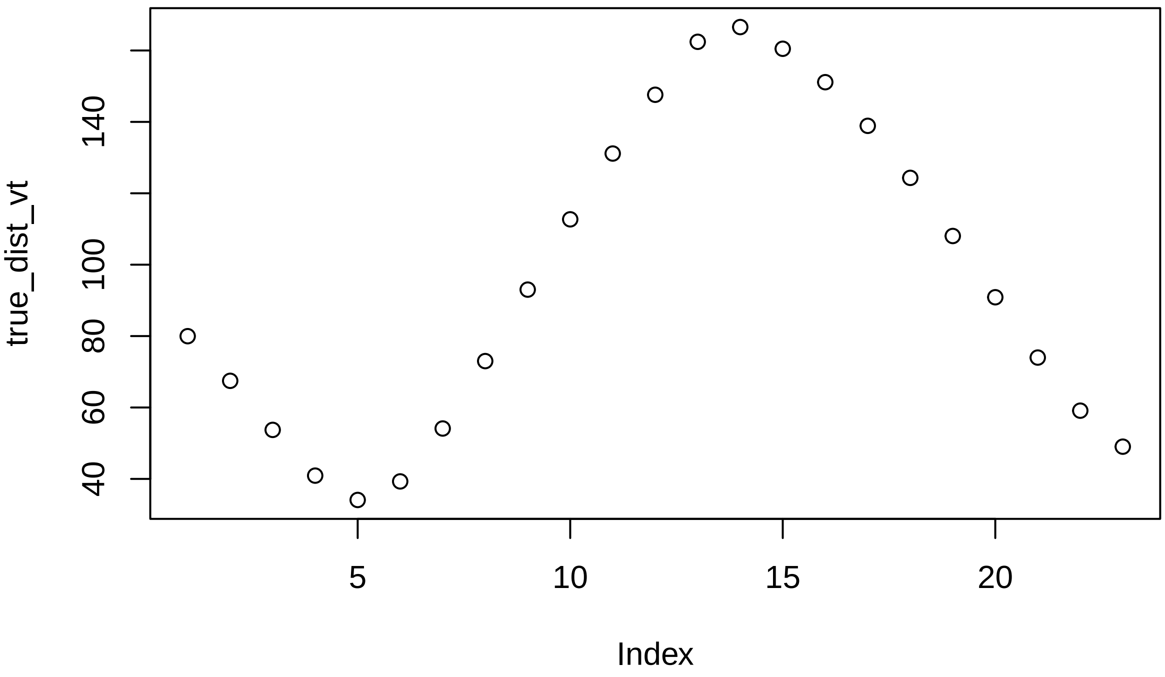


```
################################################################
#### True Proportion of Time Spent within 100m of Windmill ######
library(FNN)
knn_prop_true <-get.knnx(data = dat_mat, query = windmill_mat, k=1)
knn_prop_true <-get.knnx(data = windmill_mat, query = dat_mat, k=1)
```

```
knn_prop_true <-knnx.dist(data = windmill_mat, query = dat_mat, k=1)
str(knn_prop_true)
```

```
##  num [1:1500, 1] 343 332 321 310 300 ...
```

```
plot(knn_prop_true, ylim = c(0,1000))
lines(1:1500, rep(100, times=1500), col= "red")
```

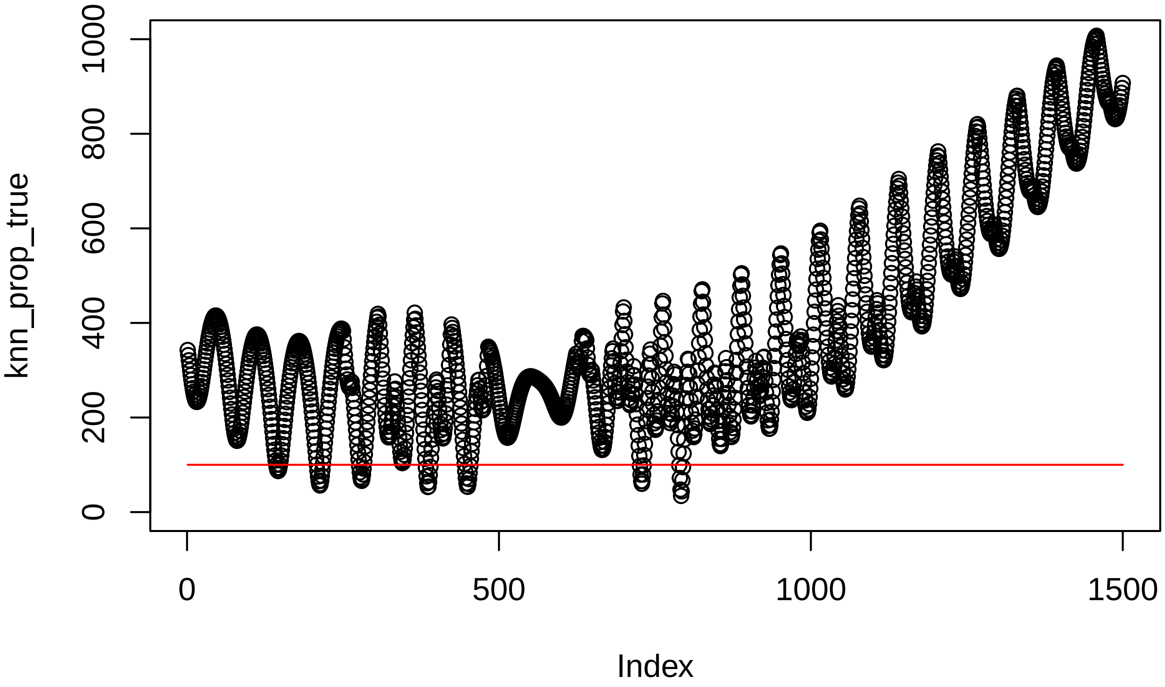


```
true_prop_100 <-  table(knn_prop_true <100)[2]/(table(knn_prop_true <100)[1]+
                                                  table(knn_prop_true <100)[2])


y_lat <- y_lat + e_tlat
y_lon <- y_lon + e_tlon

plot(y_lon, y_lat, type="l")
points(windmill_mat[,1], windmill_mat[,2], col="red")
```

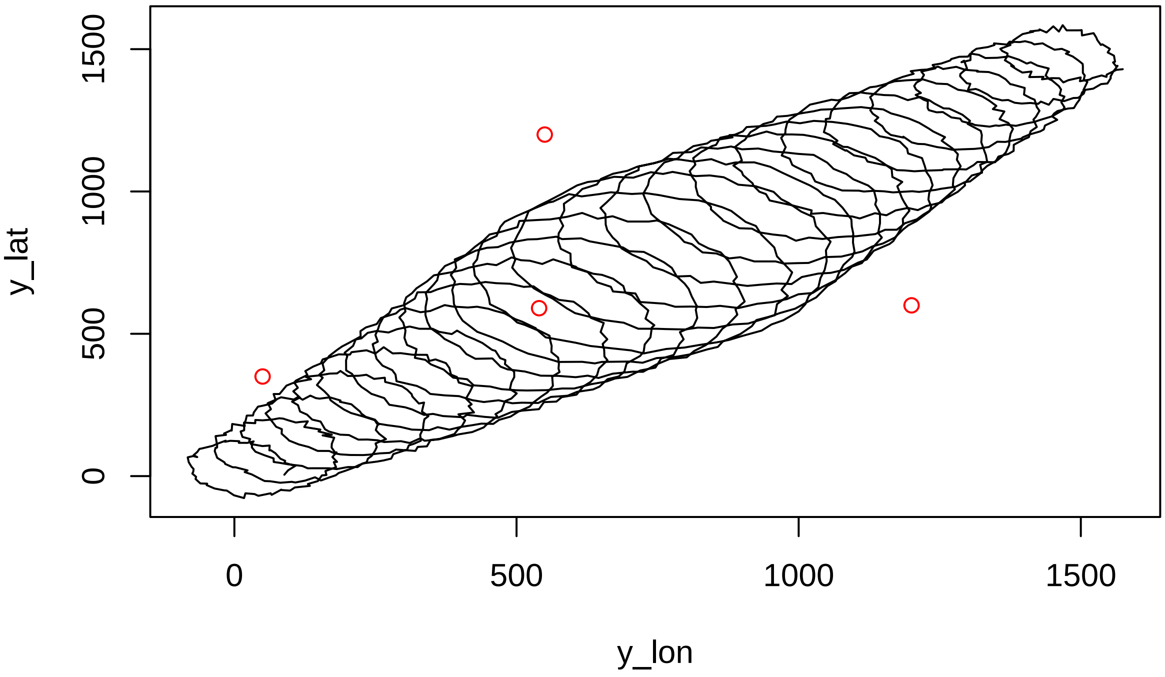

Weighted K-nearest neighbor bagging movement model function

```
library(kknn)
knn_bag_move <- function(tel_dat, bag_samps = 500, pred_time_incr =1.0,
                         K = 4, num_subsamples = 60){
  # tel_dat <- telemetry_dat
  num_time <- seq(floor(min(tel_dat$num_time)),
                  ceiling(max(tel_dat$num_time)),
                  by = pred_time_incr)
  new_dat <- as.data.frame(cbind(num_time))
  mat_pred_lon <- matrix(data=NA, nrow = bag_samps, ncol=length(num_time))
  mat_pred_lat <- matrix(data=NA, nrow = bag_samps, ncol=length(num_time))
  mat_pred_train_lon <- matrix(data=NA, nrow = bag_samps,
                               ncol=length(tel_dat$num_time))
  mat_pred_train_lat <- matrix(data=NA, nrow = bag_samps,
                               ncol=length(tel_dat$num_time))
  mat_oob_lon <- matrix(data=NA, nrow = bag_samps, ncol=length(tel_dat$num_time))
  mat_oob_lat <- matrix(data=NA, nrow = bag_samps, ncol=length(tel_dat$num_time))
  mat_oob_res_lon <- matrix(data=NA, nrow = bag_samps,
                            ncol=length(tel_dat$num_time))
  mat_oob_res_lat <- matrix(data=NA, nrow = bag_samps,
                            ncol=length(tel_dat$num_time))
  var_vt_lon <- as.numeric()
  var_vt_lat <- as.numeric()

  temp_mat_boot <- matrix(nrow = length(tel_dat$num_time),
                          ncol = bag_samps)

  list_store <- vector(length = 7, mode = "list")

  for (i in 1:bag_samps) {
```

```r
time_temp <- tel_dat$num_time
train_dat <- as.data.frame(time_temp)
names(train_dat) <- "num_time"

# get the in-bag and oob samples
subsamp_temp <- sort(rep(1:num_subsamples,
                         length.out = length(tel_dat$num_time)))
samp_inbag <- sample(1:num_subsamples, size = num_subsamples, replace=TRUE)
samp_oob <- subsamp_temp[subsamp_temp %in% samp_inbag ==FALSE]
df_subsamp_temp <- as.data.frame(cbind(1:length(tel_dat$num_time),
                                       subsamp_temp))

# which(df_subsamp_temp$subsamp_temp %in% samp_inbag)

index_vt_final <- as.numeric()
for (j in 1:length(df_subsamp_temp$subsamp_temp)) {
  # j=7
  meep <- df_subsamp_temp$subsamp_temp[j]
  if(df_subsamp_temp$subsamp_temp[j] %in% samp_inbag == TRUE){
    num_occur_temp <-sum(samp_inbag == meep)
    index_vt_final <- c(index_vt_final, rep(j, times = num_occur_temp))
  }
}
# table(index_vt_final)

# in-bag data as df for bagged model

df_in_bag_temp <- tel_dat[index_vt_final,]
# df_ex_temp <- df_ex[samp_inbag,]
#oob data as df
oob_dat <- as.data.frame(tel_dat[df_subsamp_temp$V1[subsamp_temp %in% samp_oob],])
# oob_dat <- as.data.frame(samp_oob)
# names(oob_dat) <- "num_time"
# in-bag model
knn_temp_oob_lon <- kknn(num_time~y_lon, train=df_in_bag_temp,
                         test= oob_dat, k=K, kernel = "gaussian")
knn_temp_oob_lat <- kknn(num_time~y_lat, train=df_in_bag_temp,
                         test= oob_dat, k=K, kernel = "gaussian")
knn_temp_train_lon <- kknn(num_time~y_lon, train=df_in_bag_temp,
                           test= df_in_bag_temp, k=K, kernel = "gaussian")
knn_temp_train_lat <- kknn(num_time~y_lat, train=df_in_bag_temp,
                           test= df_in_bag_temp, k=K, kernel = "gaussian")
knn_temp_pred_lon <- kknn(num_time~y_lon, train=df_in_bag_temp,
                          test= new_dat, k=K, kernel = "gaussian")
# plot(knn_temp_pred_lon$fitted.values)
knn_temp_pred_lat <- kknn(num_time~y_lat, train=df_in_bag_temp,
                          test= new_dat, k=K, kernel = "gaussian")

# get residuals for oob data
oob_pred_temp_lon <- knn_temp_oob_lon$fitted.values
oob_pred_temp_lat <- knn_temp_oob_lat$fitted.values
mat_oob_lon[i, samp_oob] <- oob_pred_temp_lon
mat_oob_lat[i, samp_oob] <- oob_pred_temp_lat
```

```
  # Store record of in-bag times used
  temp_mat_boot[,i] <- df_in_bag_temp$num_time

  # df_subsamp_temp$V1[subsamp_temp %in% samp_inbag]
  res_temp_lon <- tel_dat$y_lon[df_subsamp_temp$V1[subsamp_temp %in% samp_oob]] - oob_pred_temp_lon
  res_temp_lat <- tel_dat$y_lat[df_subsamp_temp$V1[subsamp_temp %in% samp_oob]] - oob_pred_temp_lat
  # res_temp_lon <- tel_dat$y_lon[x1_temp %in% samp_inbag ==FALSE] - oob_pred_temp_lon
  # res_temp_lat <- tel_dat$y_lat[x1_temp %in% samp_inbag ==FALSE] - oob_pred_temp_lat
  length(res_temp_lon)

  mat_oob_res_lon[i, samp_oob] <- res_temp_lon
  mat_oob_res_lat[i, samp_oob] <- res_temp_lat
  var_vt_lon <- c(var_vt_lon, var(res_temp_lon))
  var_vt_lat <- c(var_vt_lat, var(res_temp_lat))
  # predictions for time grid/test data times
  mat_pred_train_lon[i,] <- knn_temp_train_lon$fitted.values
  mat_pred_train_lat[i,] <- knn_temp_train_lat$fitted.values
  mat_pred_lon[i,]  <- knn_temp_pred_lon$fitted.values#
  mat_pred_lat[i,]  <- knn_temp_pred_lat$fitted.values#

  # print(i)
  #
  # samp_temp <- sample(1:10, size =10, replace=TRUE)
  # knn_temp <- knn.reg(train=df_ex[samp_temp,1],
  #                     test= new_dat,
  #                     y = df_ex$y1[samp_temp],
  #                     k=2)
  # mat_pred[i,]  <- knn_temp$pred
}

mat_oob_res_vt_lat <-as.vector(mat_oob_res_lat)
mat_oob_res_vt_lon <-as.vector(mat_oob_res_lon)

sd_knn_res_lat <- sd(mat_oob_res_vt_lat, na.rm = TRUE)
sd_knn_res_lon <- sd(mat_oob_res_vt_lon, na.rm = TRUE)

mat_paths_lat <- matrix(data=NA, ncol= length(num_time), nrow=bag_samps)
mat_paths_lon <- matrix(data=NA, ncol= length(num_time), nrow=bag_samps)

for (n in 1:bag_samps) {
  # n=1
  # use expected value of the animal's path and the information from the
  # prediction interval for each location to generate potential paths
  # of the animal using knn regression
  time_temp <- tel_dat$num_time
  df_numtime <- as.data.frame(cbind(1:1500,1:1500, num_time))
  names(df_numtime) <- c("location_temp_lon","location_temp_lat", "time_temp")
  location_temp_lat <- rnorm(n = length(tel_dat$y_lat),
                             mean = tel_dat$y_lat, sd = sd_knn_res_lat)
  location_temp_lon <- rnorm(n = length(tel_dat$y_lon),
                             mean = tel_dat$y_lon, sd = sd_knn_res_lon)
  knn_temp_lat <-  kknn(time_temp~location_temp_lat,
                        train=as.data.frame(cbind(time_temp, location_temp_lat)),
```

```
                          test= df_numtime, k=K, kernel = "gaussian")
    knn_temp_lon <- kknn(time_temp~location_temp_lon,
                         train=as.data.frame(cbind(time_temp, location_temp_lon)),
                         test= df_numtime, k=K, kernel = "gaussian")
    mat_paths_lat[n,] <- knn_temp_lat$fitted.values
    mat_paths_lon[n,] <- knn_temp_lon$fitted.values
  }

  list_store[[1]] <- temp_mat_boot
  list_store[[2]] <- mat_pred_lat
  list_store[[3]] <- mat_pred_lon
  list_store[[4]] <- num_time
  list_store[[5]] <- "fill with Animal ID"
  list_store[[6]] <- mat_paths_lat
  list_store[[7]] <- mat_paths_lon

  return(list_store)
}
```

Code for drifting circle simulation study

```
bagged_simulation <- function(data, samp_size = 300, num_sims = 500,
                              rad = 100, loc_err = "low",
                              num_subsamps=60, neighbors = 4){

  # data = dat_mat
  # samp_size = 150
  # num_sims = 5
  # rad = 100
  # loc_err = "high"
  # num_subsamps=10
  # neighbors = 4

  list_temp <- vector(mode = "list", length = 6)
  list_sim_sum <- vector(mode = "list", length = 6)

  # extract data dimensions
  dat_len <- dim(data)[1]
  tel_time <- 1:dat_len

  # Storage
  # Matrices for estimated animal location (lat/lon)
  mat_lon <- matrix(data = NA, nrow = num_sims, ncol = dat_len)
  mat_lat <- matrix(data = NA, nrow = num_sims, ncol = dat_len)
  # Matrice for estimated daily displacement
  mat_daily_disp <- matrix(data = NA, nrow = num_sims, ncol = dat_len/(60)-2)
  # Vector for CP  of daily displacement on disperal day
  # disp_covered <- as.character()
  disp_covered_mat <- matrix(nrow = num_sims, ncol = (dat_len/60) - 2)
  # Vector for proportion of time spent within r dist of object
  prop_time_r <- as.numeric()
  # Vector for CP of prop time
  prop_covered <- as.character()
```

```r
# numcores <- 5
# registerDoParallel(numcores)
startTime <- Sys.time()
# #c(2,4,6)
# # 2:41
# r <- foreach (j = 1:num_sims) %dopar% {
for (j in 1:num_sims) {


  # j=1
  tel_time_temp <- sort(c(tel_time[1],
                        sample(tel_time[2]:tel_time[dat_len-1],
                               size = samp_size-2), tel_time[dat_len]))
  telemetry_dat <- as.data.frame(dat_mat[tel_time_temp,])
  telemetry_dat$num_time <- tel_time_temp
  if(loc_err == "low"){
    # Add device location error with randomly selected distribution
    for (i in 1:length(tel_time_temp)) {
      # for (i in 1:300) {
      dist_choice <- sample(c("Normal", "Triangular", "Uniform"), size =1)
      if(dist_choice == "Normal"){
        e_dlat <- rnorm(1, mean = 0, sd = 10)
        e_dlon <- rnorm(1, mean = 0, sd = 10)
        telemetry_dat$y_lon[i] <- telemetry_dat$y_lon[i] + e_dlat
        telemetry_dat$y_lat[i] <- telemetry_dat$y_lat[i] + e_dlon
      }
      if(dist_choice == "Triangular"){
        e_dlat <- rtri(1, min = -12, max=12, mode = 0)
        e_dlon <- rtri(1, min = -12, max=12, mode = 0)
        telemetry_dat$y_lon[i] <- telemetry_dat$y_lon[i] + e_dlat
        telemetry_dat$y_lat[i] <- telemetry_dat$y_lat[i] + e_dlon
      }
      if(dist_choice == "Uniform"){
        e_dlat <- runif(1, min = -15, max = 15)
        e_dlon <- runif(1, min = -15, max = 15)
        telemetry_dat$y_lon[i] <- telemetry_dat$y_lon[i] + e_dlat
        telemetry_dat$y_lat[i] <- telemetry_dat$y_lat[i] + e_dlon
      }
    }

  }
  if(loc_err == "high"){
    for (i in 1:length(tel_time_temp)) {
      # for (i in 1:300) {
      dist_choice <- sample(c("Normal", "Triangular", "Uniform"), size =1)
      if(dist_choice == "Normal"){
        e_dlat <- rnorm(1, mean = 0, sd = 100)
        e_dlon <- rnorm(1, mean = 0, sd = 100)
        telemetry_dat$y_lon[i] <- telemetry_dat$y_lon[i] + e_dlat
        telemetry_dat$y_lat[i] <- telemetry_dat$y_lat[i] + e_dlon
      }
      if(dist_choice == "Triangular"){
        e_dlat <- rtri(1, min = -12, max=120, mode = 0)
```

```
        e_dlon <- rtri(1, min = -12, max=120, mode = 0)
        telemetry_dat$y_lon[i] <- telemetry_dat$y_lon[i] + e_dlat
        telemetry_dat$y_lat[i] <- telemetry_dat$y_lat[i] + e_dlon
      }
      if(dist_choice == "Uniform"){
        e_dlat <- runif(1, min = -150, max = 150)
        e_dlon <- runif(1, min = -150, max = 150)
        telemetry_dat$y_lon[i] <- telemetry_dat$y_lon[i] + e_dlat
        telemetry_dat$y_lat[i] <- telemetry_dat$y_lat[i] + e_dlon
      }
    }
  }
  # print(j)
  # # telemetry_dat$num_time <- as.numeric(telemetry_dat$num_time)
  # print(telemetry_dat)
  move_mod_temp <- knn_bag_move(tel_dat = telemetry_dat,
                                K=neighbors, num_subsamples = num_subsamps)
  # Location estimation
  f.bar.1lat <- colMeans((move_mod_temp[[6]]))
  f.bar.1lon <- colMeans((move_mod_temp[[7]]))
  f.CI.1lat <- t(apply(move_mod_temp[[6]],
                       2, FUN = quantile, prob = c(0.025, 0.975)))
  # Displacement Estimation
  daily_disp_subset <- seq(1,dat_len, by=60)
  disp_mat_dist <- matrix(nrow=500, ncol=length(daily_disp_subset)-2)

  for (u in 1:500) {
    # u=1
    disp_locations <- cbind(move_mod_temp[[7]][u, daily_disp_subset],
                            move_mod_temp[[6]][u,daily_disp_subset])
    disp_mat <- as.matrix(dist(disp_locations))
    dist_vt <- as.numeric()

    for (v in 1:(length(disp_locations[,1])-2)) {
      dist_vt <- c(dist_vt, disp_mat[v, v+1])
    }
    disp_mat_dist[u,] <- dist_vt

  }

  f.bar.disp <- colMeans(disp_mat_dist)
  f.CI.disp <- t(apply(disp_mat_dist,
                       2, FUN = quantile, prob = c(0.025, 0.975)))

  disp_covered_vt <- as.character()
  for (b in 1:length(f.bar.disp)) {
    disp_covered_temp <- check.in.range(true_dist_vt[b],
                                        f.CI.disp[b,],
                                        fatal = FALSE)
    disp_covered_vt <- c(disp_covered_vt, disp_covered_temp)
  }

  # print(j)
```

```r
  # Estimation of Proportion of time within r dist
  knn_mat <- matrix(nrow = dim(move_mod_temp[[2]])[2],
                    ncol = dim(move_mod_temp[[2]])[1])
  prop_wthn_100m <- as.numeric()

  for (i in 1:dim(move_mod_temp[[7]])[1]) {
    # i=1
    mat_temp <- cbind(move_mod_temp[[7]][i,],
                      move_mod_temp[[6]][i,])
    # head(mat_temp)
    knn_temp <-knnx.dist(data = windmill_mat, query = mat_temp, k=1)

    prop_wthn_100m <- c(prop_wthn_100m,
                        as.numeric(table(knn_temp < rad)[2]/
                                     (table(knn_temp < rad)[2]+
                                        table(knn_temp < rad)[1])))
    knn_mat[,i] <- knn_temp
  }

                                    table(knn_prop_true <200)[2]))
  prop_time_temp <- mean(prop_wthn_100m, na.rm = TRUE)
  cp_temp <-quantile(prop_wthn_100m, probs = c(0.025, 0.975), na.rm = TRUE)

  prop_covered_temp <- check.in.range(true_prop_100,cp_temp,
                                      fatal = FALSE)


  # Store Important Information for Simulation Summary
  # CIs for Location
  mat_lon[j,] <- f.bar.1lon
  mat_lat[j,] <- f.bar.1lat
  # CIs/Point estimates of daily displacement
  mat_daily_disp[j,] <- f.bar.disp
  # CP information for Daily Displacement on Extreme Movement Day
  # disp_covered <- c(disp_covered, disp_covered_temp)
  disp_covered_mat[j,] <- disp_covered_vt
  # CIs/Point Estimates of Prop Time
  prop_time_r <- c(prop_time_r, prop_time_temp)
  # CP information for Prop Time
  prop_covered <- c(prop_covered, prop_covered_temp)

  list_temp[[6]]  <- prop_covered
  if(j%%10 == 0){
    endTime <- Sys.time()
    t_check <- endTime - startTime
    time_update <- paste0("Sim", " ", j, ": Elasped Time =", t_check)
    print(time_update)}
  # else{print(j)}
}


# Store in cool list object
list_sim_sum[[1]] <- mat_lat
```

```
  list_sim_sum[[2]]  <- mat_lon
  list_sim_sum[[3]]  <- mat_daily_disp
  list_sim_sum[[4]]  <- disp_covered_mat
  list_sim_sum[[5]]  <- prop_time_r
  list_sim_sum[[6]]  <- prop_covered

  return(list_sim_sum)
}
```

Code for visualization of results

```
set.seed(173)
sim_higherr_150 <- bagged_simulation(data = dat_mat, samp_size = 150,
                                     num_sims = 500, rad = 100,
                                     loc_err = "high",
                                     num_subsamps=50, neighbors = 4)

set.seed(4061)
sim_lowerr_150 <- bagged_simulation(data = dat_mat, samp_size = 150,
                                    num_sims = 500, rad = 100,
                                    loc_err = "low",
                                    num_subsamps=50, neighbors = 4)

set.seed(3770)
sim_higherr_30 <- bagged_simulation(data = dat_mat, samp_size = 30,
                                     num_sims = 500, rad = 100,
                                     loc_err = "high",
                                     num_subsamps=30, neighbors = 4)

set.seed(465)
sim_lowerr_30 <- bagged_simulation(data = dat_mat, samp_size = 30,
                                    num_sims = 500, rad = 100,
                                    loc_err = "low",
                                    num_subsamps=30, neighbors = 4)
```

Code for Visualization of Results

```
gg_temp <- ggplot() +
  theme(text = element_text(family="Times",size=20),
        plot.title = element_text(size = 20),
        axis.text.x=element_text(size=20),
        axis.text.y=element_text(size=20),
        panel.background = element_rect(fill = "white", colour = "black"),
        panel.grid.major = element_blank(),
        panel.grid.major.y=element_blank(),
        legend.position = "none")

# extract data dimensions
dat_len <- dim(dat_mat)[1]
tel_time <- 1:dat_len
tel_time_temp <- sort(c(tel_time[1],
                        sample(tel_time[2]:tel_time[dat_len-1],
                               size = 30-2), tel_time[dat_len]))
```

```
telemetry_dat <- as.data.frame(dat_mat[tel_time_temp,])
telemetry_dat$num_time <- tel_time_temp
for (i in 1:length(tel_time_temp)) {
  # for (i in 1:300) {
  dist_choice <- sample(c("Normal", "Triangular", "Uniform"), size =1)
  if(dist_choice == "Normal"){
    e_dlat <- rnorm(1, mean = 0, sd = 100)
    e_dlon <- rnorm(1, mean = 0, sd = 100)
    telemetry_dat$y_lon[i] <- telemetry_dat$y_lon[i] + e_dlat
    telemetry_dat$y_lat[i] <- telemetry_dat$y_lat[i] + e_dlon
  }
  if(dist_choice == "Triangular"){
    e_dlat <- rtri(1, min = -120, max=120, mode = 0)
    e_dlon <- rtri(1, min = -120, max=120, mode = 0)
    telemetry_dat$y_lon[i] <- telemetry_dat$y_lon[i] + e_dlat
    telemetry_dat$y_lat[i] <- telemetry_dat$y_lat[i] + e_dlon
  }
  if(dist_choice == "Uniform"){
    e_dlat <- runif(1, min = -150, max = 150)
    e_dlon <- runif(1, min = -150, max = 150)
    telemetry_dat$y_lon[i] <- telemetry_dat$y_lon[i] + e_dlat
    telemetry_dat$y_lat[i] <- telemetry_dat$y_lat[i] + e_dlon
  }
}



gg_map <- gg_temp +
  geom_point(data=telemetry_dat, aes(x=y_lon, y=y_lat),
             alpha =0.4, color = "darkorange", size = 6)+
  geom_point(data = as.data.frame(windmill_mat),
            aes(x=V1, y=V2), col = "lemonchiffon4", size = 9.0, shape=8) +
  geom_path(data = as.data.frame(dat_mat), aes(x=y_lon, y=y_lat),
            color = "mediumblue", size=1.2) +
  ylab("Y (m)") + xlab("X (m)")


gg_disp <- gg_temp +
  geom_violin(aes(x= "High Loc Err:\n n=150", y=sim_higherr_150[[3]]),
              fill = "turquoise", alpha=0.3) +
  geom_boxplot(aes(x= "High Loc Err:\n n=150", y=sim_higherr_150[[3]]),
               width =0.4) +
  geom_violin(aes(x= "High Loc Err:\n n=30", y=sim_higherr_30[[3]]),
              fill = "turquoise", alpha=0.3) +
  geom_boxplot(aes(x= "High Loc Err:\n n=30", y=sim_higherr_30[[3]]),
               width =0.4) +
  geom_violin(aes(x= "Low Loc Err:\n n=150", y=sim_lowerr_150[[3]]),
              fill = "turquoise", alpha=0.3) +
  geom_boxplot(aes(x= "Low Loc Err:\n n=150", y=sim_lowerr_150[[3]]),
               width =0.4) +
  geom_violin(aes(x= "Low Loc Err:\n n=30", y=sim_lowerr_30[[3]]),
              fill = "turquoise", alpha=0.3) +
  geom_boxplot(aes(x= "Low Loc Err:\n n=30", y=sim_lowerr_30[[3]]), width =0.4) +
```

```r
  geom_hline(yintercept = 96.16755, linetype = "dashed")+
  ylab("Average Minutely Displacement (m)") + xlab(" ")

gg_p100 <- gg_temp +
  geom_violin(aes(x= "High Loc Err:\n n=150", y=sim_higherr_150[[5]]),
              fill = "goldenrod4", alpha=0.3) +
  geom_boxplot(aes(x= "High Loc Err:\n n=150", y=sim_higherr_150[[5]]),
               width =0.4) +
  geom_violin(aes(x= "High Loc Err:\n n=30", y=sim_higherr_30[[5]]),
              fill = "goldenrod4", alpha=0.3) +
  geom_boxplot(aes(x= "High Loc Err:\n n=30", y=sim_higherr_30[[5]]),
               width =0.4) +
  geom_violin(aes(x= "Low Loc Err:\n n=150", y=sim_lowerr_150[[5]]),
              fill = "goldenrod4", alpha=0.3) +
  geom_boxplot(aes(x= "Low Loc Err:\n n=150", y=sim_lowerr_150[[5]]),
               width =0.4) +
  geom_violin(aes(x= "Low Loc Err:\n n=30", y=sim_lowerr_30[[5]]),
              fill = "goldenrod4", alpha=0.3) +
  geom_boxplot(aes(x= "Low Loc Err:\n n=30", y=sim_lowerr_30[[5]]),
               width =0.4) +
  geom_hline(yintercept = 0.0353, linetype = "dashed")+
  ylab("Proportion of Time wthn 100m of Windwills") + xlab("")

gg_lower <-grid.arrange(gg_disp, gg_p100, ncol=2)

#setwd("/...")
#png("simulation_drift_circ.png", width = 3200, height = 3600, res = 200)
grid.arrange(gg_map, gg_lower, ncol=1)
#dev.off()
```

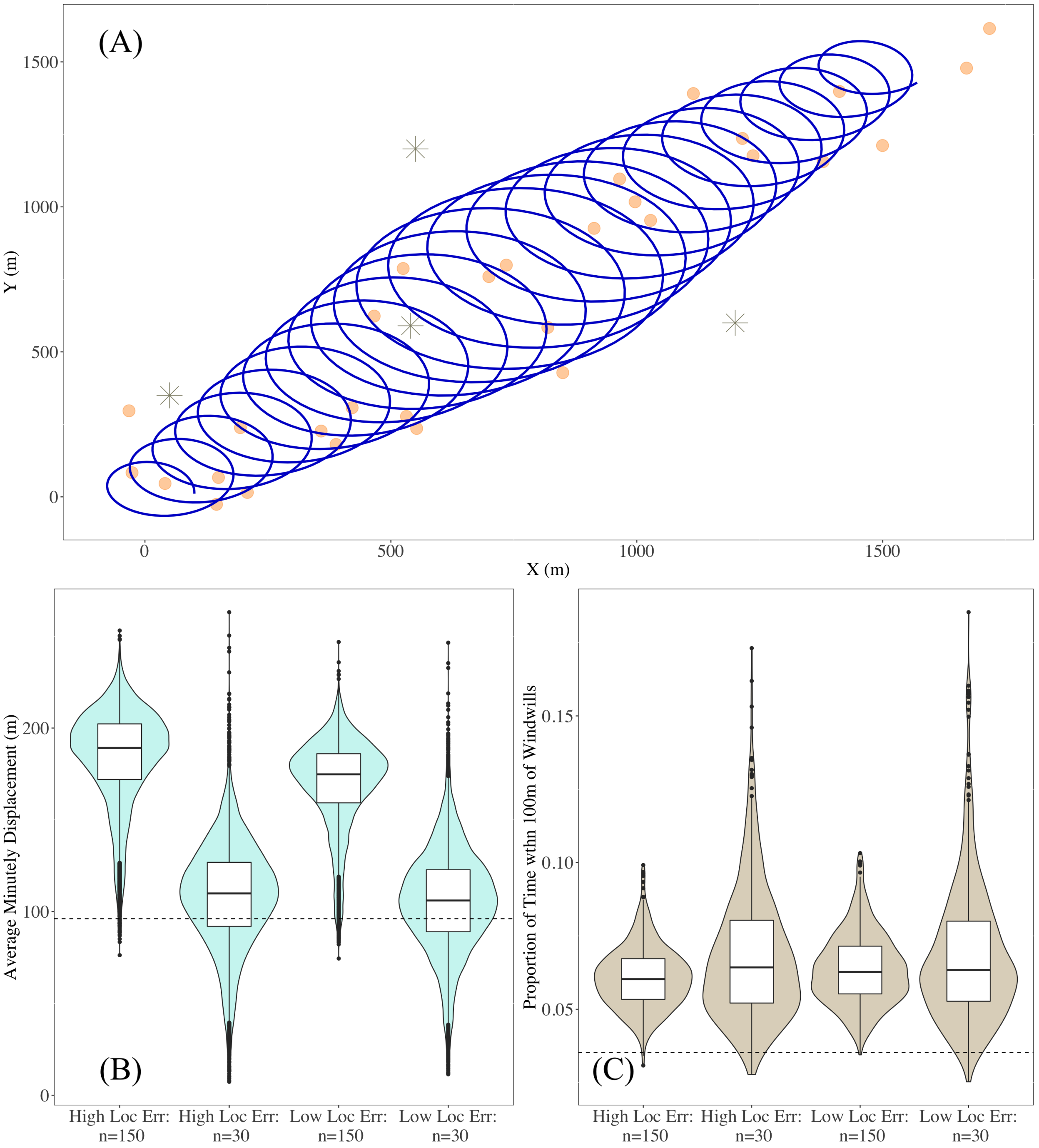
(A)
Y (m)
X (m)
(B)
Average Minutely Displacement (m)
(C)
Proportion of Time wthn 100m of Windwills
High Loc Err: n=150
High Loc Err: n=30
Low Loc Err: n=150
Low Loc Err: n=30

Section 5: Disclaimer about adapting bagging functions for applied use

Generally, many telemetry datasets have similar structure. There is a column representing time (usually as a date) and two to three columns representing location (e.g., longitude, latitude, and perhaps altitude). The bagged k-nearest neighbor functions used in this appendix can be used for real telemetry data with two columns for animal location. However, there may be multiple formatting hurdles that may need to be addressed in order to successfully translate code shown here to applied use.

Date-time formatting can vary greatly between various sources of telemetry data. We recommended converting date-time formatting to some form of numeric time (where there is a $t = 0$ and units of t could be seconds, minutes or hours). There are various ways to accomplish this task. We have found that package such as the `lubridate` R-package can help expedite this process. There may be other data cleaning tasks required to successfully translate code used from this section to applied use. We encourage readers to peruse Appendix 1 for some examples of how this is accomplished (e.g., the king rail and mule deer telemetry data examples).
